# Y(OTf)_3_-Salazin-Catalyzed Asymmetric Aldol Condensation

**DOI:** 10.3390/molecules29091963

**Published:** 2024-04-25

**Authors:** Chengzhuo Wang, Ning Chen, Zhanhui Yang, Jiaxi Xu

**Affiliations:** State Key Laboratory of Chemical Resource Engineering, Department of Organic Chemistry, College of Chemistry, Beijing University of Chemical Technology, Beijing 100029, China; 2020130044@buct.edu.cn (C.W.); chenning@buct.edu.cn (N.C.); zhyang@buct.edu.cn (Z.Y.)

**Keywords:** aldol condensation, aromatic aldehyde, asymmetric catalysis, aziridine, hydroxyketone, ketone, tridentate ligand, yttrium triflate

## Abstract

The chiral aziridine-containing vicinal iminophenol tridentate ligands (named salazins) are a class of readily prepared chiral ligands from enantiopure aziridines and salicylaldehydes. Their scandium and yttrium triflate complexes show excellent reactivity and enantioselectivities in the catalytic asymmetric aldol condensation of electron-deficient aromatic aldehydes and ketones, including acetone and cycloalkanones. The stereoselectivity is rationalized to the strong π–stacking interaction between aromatic aldehydes and the vicinal iminophenol group in the chiral ligands.

## 1. Introduction

**Scheme 1:**
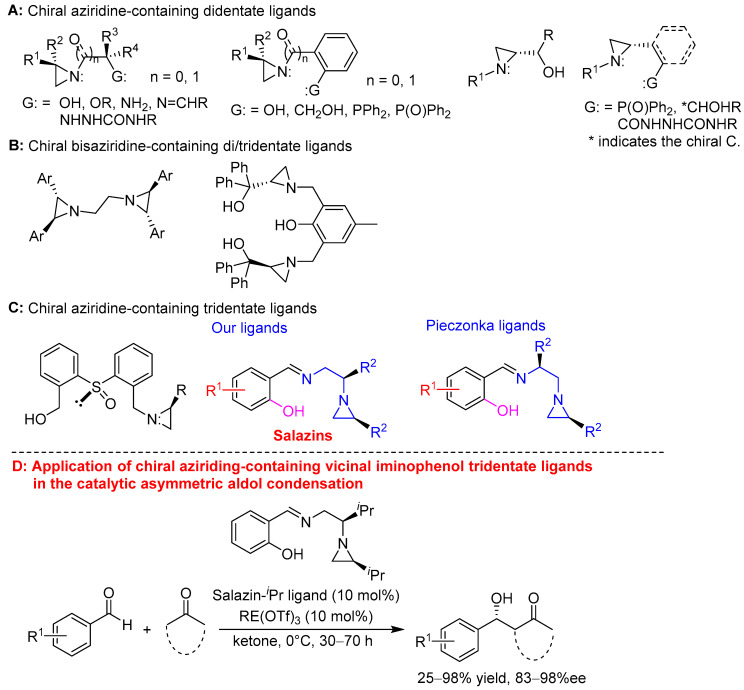
Aziridine-containing chiral ligands and application of chiral aziridine-containing vicinal iminophenol tridentate ligands in the catalytic asymmetric aldol condensation.

Aziridine-containing chiral ligands are an important class of chiral ligands in asymmetric catalysis and have been applied in various asymmetric transformations [1,2] because enantiopure aziridines are easily prepared chiral resources from commercially available vicinal amino alcohols [3,4]. Most developed aziridine-containing chiral ligands are chiral bidentate ligands, including chiral aziridine-containing alcohols [5,6,7], ethers [8,9,10], phenols [11], amines [12], imines [13], semicarbazides [14,15], phosphines [16,17], phosphine oxides [18] (Scheme 1A), and aziridines, which are actually bis-aziridine ligands [19,20,21,22] (Scheme 1B. alternatively, multidentate phenol-derived bis-aziridine-2-methanol chiral ligand has also developed [5] (Scheme 1B)). However, only limited aziridine-containing chiral tridentate ligands have been designed and synthesized, for instance, 2-[2-(aziridine-1-methyl)benzenesulfinyl]benzyl alcohols [23,24,25] and two classes of aziridine-containing vicinal iminophenol tridentate ligands [13,26] (Scheme 1C). Although various chiral aziridine-containing ligands have been prepared and applied in several asymmetric catalysis, their applications are still limited to date due to their complicated and multiple synthetic steps. To enrich the aziridine-containing chiral ligands, recently, our research group synthesized a series of chiral aziridine-containing vicinal iminophenol tridentate ligands in one step from readily prepared enantiopure aziridines and salicylaldehydes. The regiospecific cleavage of the more substituted C–N bond of aziridines was enabled by an iminium-mediated self-ring opening reaction with the complete inversion of configuration, because nucleophilic ring-opening is an electronic effect-controlled process (Scheme 1C, middle ones) [26,27]. In short, our tridentate ligands were named salazins because they were prepared directly from salicylaldehydes and aziridines. They exhibited good to excellent enantioselectivity in the Zn(OTf)_2_-catalyzed asymmetric aldol condensation [26].

Lewis acids generally play an important role in their unique catalytic reactivity and selectivity in catalytic organic reactions. In comparison with traditional Lewis acids of transition metal ions, rare-earth metal ions generally show strong Lewis acidity and oxophilicity. The rare-earth metal triflates are more appealing from a synthetic point of view in acid-/Lewis acid-catalyzed organic reactions. In the past three decades, rare-earth metal triflates, including scandium triflate, yttrium triflate, and lanthanide (La–Lu) triflates, etc., have been widely utilized in the organic chemistry [28,29]. China has abundant rare-earth metal resources. It is in demand to develop their application in catalytic organic reactions, especially catalytic asymmetric organic reactions.

## 2. Results and Discussion

When we first prepared our chiral aziridine-containing vicinal iminophenol tridentate ligands (salazins), they were evaluated with a catalytic asymmetric aldol condensation of aromatic aldehydes and acetone in the presence of Zn(OTf)_2_, affording β-hydroxyketones in good to excellent yields and enantioselectivities [26]. Because the rare-earth metal ions generally show stronger Lewis acidity and oxophilicity than transition metal ions [28], we rationalized that rare-earth metal triflates would take advantage in the Lewis acid-catalyzed aldol condensation. To develop the further application of our chiral tridentate ligands and rare-earth metals, we conducted a rare-earth metal triflate chiral tridentate ligand complex-catalyzed asymmetric aldol condensation. The reaction of 4-nitrobenzaldehyde (**1a**) and acetone was selected as a model reaction to optimize reaction conditions. The solvent screening was first carried out with the Sc(OTf)_3_-salazin-Bn complex as the catalyst (Table 1). The ligand salazin-Bn was prepared from salicylaldehyde and (*S*)-benzylaziridine. When toluene was used as solvent, the aldol adduct **3a** was obtained in only a 3% yield with 38% ee (Table 1, entry 1). The adduct **3a** was obtained in an excellent yield of 98%, but moderate 53% ee, in EtOH (Table 1, entry 2). When the solvent was changed to DCM and THF, the adduct was generated in 10% and 20% yields with 60% ee and 49% ee, respectively (Table 1, entries 3 and 4). Furthermore, an excellent yield of 96% and good enantioselectivity (87% ee) were obtained when the reaction was performed in acetone (Table 1, entry 5). The results indicated that acetone, in which 87% ee was obtained, was the best choice as the solvent for the asymmetric reaction.

For convenience, the chiral ligands and rare-earth metal triflates were further optimized in acetone with the model reaction at room temperature (15 °C) (Table 2, entries 1–14). The reaction of 4-nitrobenzaldehyde (**1a**) and acetone under the catalysis of Sc(OTf)_3_ and ligand salazin-Bn generated adduct **3a** in a 96% yield with 81% ee (Table 2, entry 1). Under the catalysis of Sc(OTf)_3_ with ligands salazin-*^t^*Bu-Bn and salazin-Br-Bn, the adduct **3a** was obtained in a 96% yield with 70% ee and a 55% yield with 78% ee, respectively (Table 2, entries 2 and 3). The reaction gave adduct **3a** in a 91% yield with 86% ee and an 89% yield with 87% ee, respectively, under the catalysis of Sc(OTf)_3_ with ligands salazin-NO_2_-*^i^*Pr and salazin-Br-*^i^*Pr (Table 2, entries 4 and 5). However, under the catalysis of Sc(OTf)_3_ with ligand salazin-I-*^i^*Pr, both the yield (60%) and enantioselectivity (80% ee) of the adduct **3a** decreased obviously (Table 2, entry 6). The reaction catalyzed by Sc(OTf)_3_ with unsubstituted ligand salazin-*^i^*Pr produced adduct **3a** in 98% with 88% ee, showing the best results (Table 2, entry 7). TLC monitoring the reaction mixture indicated that the aldehyde **1a** disappeared completely for 26 h, revealing that the ligand salazin-*^i^*Pr was more efficient. Thus, further screening of different rare-earth metal triflates was conducted with ligand salazin-*^i^*Pr, and the reaction was stirred at 15 °C for 26 h. The Y(OTf)_3_-catalyzed reaction gave rise to the same results as the Sc(OTf)_3_-catalyzed one (Table 2, entry 8). Other rare-earth metal triflates, including La(OTf)_3_, Ce(OTf)_3_, Eu(OTf)_3_, Gd(OTf)_3_, and Lu(OTf)_3_, were also attempted. Although excellent yields (91–98%) and enantioselectivities (83–87% ee) were observed, all of them are lower than those in the Sc(OTf)_3_ and Y(OTf)_3_-catalyzed reactions (Table 2, entries 9–13). In comparison with the transition metal catalyst Zn(OTf)_2_, the model reaction was conducted under the catalysis of Zn(OTf)_2_ and salazin-*^i^*Pr, giving adduct **3a** in a 90% yield and 70% ee (Table 2, entry 14). Both the yield and enantioselectivity were lower than those in the tested rare-earth metal triflate-catalyzed reactions. Finally, the reaction temperature was optimized, as well (Table 2, entries 15–20). The reactions under the catalysis of Sc(OTf)_3_ and ligand salazin-*^i^*Pr generated adduct **3a** in the same excellent yield of 98%, but different good to excellent enantioselectivities, 85% ee at 40 °C, 88% ee at 20 °C, and 98% ee at 0 °C (Table 2, entries 15–17). Similarly, the reactions under the catalysis of Y(OTf)_3_ and ligand salazin-*^i^*Pr gave adduct **3a** in excellent yields of 96–98%, but different good to excellent enantioselectivities, 89% ee at 40 °C, 88% ee at 20 °C, and 98% ee at 0 °C (Table 2, entries 18–20). All the reaction times were determined by the results of TLC monitoring the reaction mixtures. The results reveal that the reaction rates increase along with the elevation of the reaction temperature. The results illustrate that both catalytic combinations of Sc(OTf)_3_ and Y(OTf)_3_ with ligand salazin-*^i^*Pr at 0 °C are the best choice. However, considering that the metal scandium is more expensive than the metal yttrium, the catalytic system of Y(OTf)_3_ and ligand salazin-*^i^*Pr was selected as the optimal combination in the asymmetric aldol condensation of aldehydes and ketones at 0 °C.

**Scheme 2:**
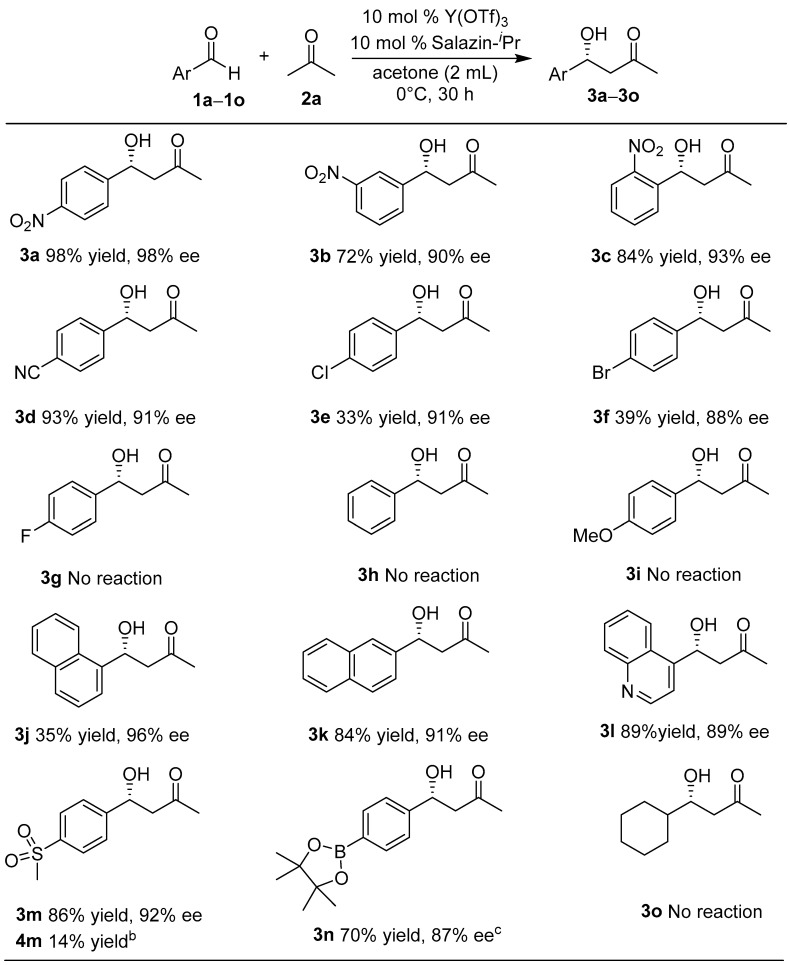
Catalytic asymmetric aldol condensation under the catalysis of Y(OTf)_3_ ^a^. ^a^ Reactions were conducted on a 0.2 mmol scale of aldehyde in 2 mL of acetone. The yields were obtained using chromatography on silica gel. ^b^ Dehydrated side product 4-(4-methanesulfonylphenyl)but-3-en-2-one (**4m**) was obtained in a 14% yield. ^c^ Reaction was conducted for 70 h.

With the optimal conditions in hand, the scope of aldehydes **1** was examined (Scheme 2). Similar to 4-nitrobenzaldehyde (**1a**), strong electron-deficient 3-nitro and 2-nitrobenzaldehydes (**1b** and **1c**) generated the corresponding adducts **3b** and **3c** in good yields of 72% and 84%, with excellent enantioselectivities of 90% ee and 93% ee. Both yields and enantioselectivities are lower than those of 4-nitrobenzaldehyde (**1a**). 4-Cyanobenzaldehydes (**1d**) led to the desired adduct **3d** in an excellent yield (93%) and enantioselectivity (91% ee). Differently, weak electron-deficient 4-chloro- and 4-bromobenzaldehydes (**1e** and **1f**) gave rise to the expected adducts **3e** and **3f** in low yields of 33% and 39%, but good to excellent enantioselectivities of 91% ee and 88% ee, respectively. However, weak electron-deficient 4-fluorobenzaldehyde (**1g**), benzaldehyde (**1g**), and electron-rich 4-methoxybenzaldehyde (**1i**) did not undergo the asymmetric reactions under the same reaction conditions, showing the electronic effect-dependent substrate selectivity possibly due to weak π-stacking interaction between these aromatic aldehydes and the benzene ring in the ligand backbone, similar to that in the catalytic asymmetric aziridination of chalcones [30]. Although the fluorine element has the strongest electronegativity, it presents weak electron-withdrawing ability in fluoroarenes, such as 4-fluorobenzaldehyde and 4-fluorobenzoic acid, because it exists in the same period with carbon in the periodic table. Its 2p orbital can overlap very well with the 2p orbital of the carbon atom in the arene ring, exhibiting strong electron-donating conjugative effect, which neutralizes its strong electron-withdrawing inductive effect. As a result, the fluoro group presents as a weak electron-withdrawing group. It can be seen from that the Hammett constant of F is smaller than those of Cl and Br, and the acidity of 4-fluorobenzoic acid is weaker than those of 4-chloro and 4-bromobenzoic acids. Furthermore, both bicyclic fused naphthalene-1-carbaldehyde (**1j**) and naphthalene-2-carbaldehyde (**1k**) proceeded well the reaction, affording the corresponding adducts **3j** in a 35% yield with 96% ee and **3k** in an 84% yield with 91% ee, respectively. In addition, bicyclic fused quinoline-4-carbaldehyde (**1l**) also worked well, giving the desired adduct **3l** in an 89% yield with 89% ee. All bicyclic fused arene and heteroarenecarbaldehydes showed excellent reactivities and stereoselectivities, possibly due to the existence of strong π-stacking interaction between these (hetero)arenecarbaldehydes and the benzene ring in the ligand backbone. To further extend the scope of aldehydes, two functionalized benzaldehydes, 4-methanesulfonylbenzaldehyde (**1m**) and 4-(4,4,5,5-tetramethyl-1,3,2-dioxaborolan-2-yl)benzaldehyde (**1n**), were examined, affording the expected adducts **3m** in an 86% yield and 92% ee, with a dehydrated side product 4-(4-methanesulfonylphenyl)but-3-en-2-one (**4m**) 14% yield and **3n** in a 70% yield and 87% ee, respectively. The aldehyde **1n** required long reaction time of 70 h. The product **3n** cannot be obtained under basic catalyzed asymmetric conditions due to the existence of base sensitive methanesulfonyl group. It is a useful synthetic building block in various organic transformations. To test the scope of aldehydes, aliphatic cyclohexanecarbaldehyde (**1o**) was attempted, but no reaction occurred due to the absence of the π-stacking interaction between the aldehyde and the benzene ring in the ligand backbone.

**Scheme 3:**
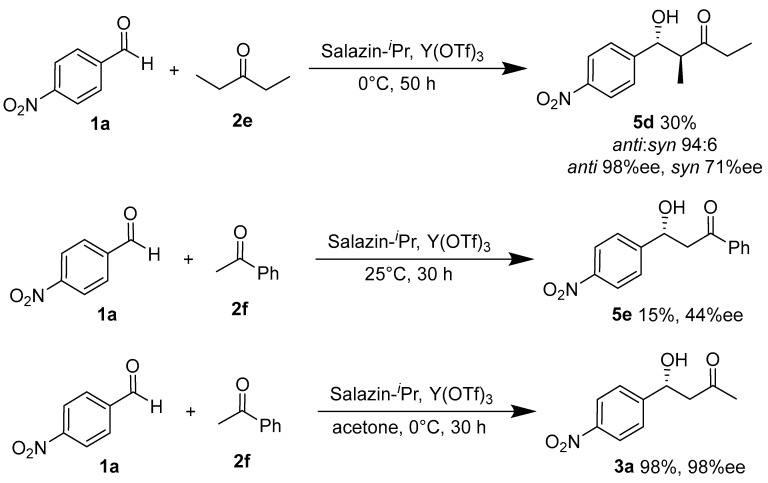
Catalytic asymmetric aldol condensations of 4-benzaldehyde with pentan-3-one and acetophenone.

To investigate the scope of ketones, several cycloalkanones **2b**–**2d** were evaluated in the reaction with 4-nitrobenzaldehyde (**1a**) under the catalysis of Y(OTf)_3_ and ligand salazin-*^i^*Pr (Table 3). The results indicate that cyclobutanone (**2b**) exhibits poor reactivity (25% yield) and diastereoselectivity (*anti*:*syn* 53:47) but moderate to good enantioselectivities (83% ee and 69% ee) for the diastereomeric products *anti*-**5a** and *syn*-**5a**, respectively (Table 3, entry 1). However, the reactions of cyclopentanone (**2c**) and cyclohexanone (**2d**) show good to excellent diastereoselectivities (the ratio of *anti*:*syn* diastereomers 83:17 and 95:6, respectively) and enantioselectivities (96% ee and 91% ee) for the *anti*-diastereomeric products *anti*-**5b** and *anti*-**5c**, respectively (Table 3, entries 2 and 3). The stereostructures of *anti*- and *syn*-diastereomers were identified in comparison with the reported NMR data [31,32,33]. Linear pentan-3-one (**2e**) was also subjected to the catalytic asymmetric reaction with 4-nitrobenzaldehyde (**1a**) at 0 °C for 50 h, affording the desired product **5d** in a low yield of 30% due to steric hindrance, with a diastereomeric ratio of *anti*:*syn* = 94:6 and enantioselectivities of 98% ee and 71% ee for *anti*-**5d** and *syn*-**5d**, respectively. The results indicate that *anti*-diastereomeric products generally show higher enantioselectivities than the corresponding *syn*-diastereomeric ones. Aromatic acetophenone (**2f**) was evaluated in the asymmetric catalysis, with aldehyde **1a,** as well. The asymmetric reaction was conducted at room temperature (at 25 °C) for 30 h because the melting point of acetophenone is 20 °C. The expected product **5e** was obtained in only a 15% yield with 44% ee. The asymmetric catalytic reaction of **1a** and **2f** was also performed in acetone as a solvent at 0 °C for 30 h. However, only aldol adduct **3a** was obtained in a 98% yield and 98% ee. The results reveal that acetone is more active than acetophenone in the asymmetric catalytic aldol condensation with 4-nitrobenzaldehyde (**1a**) (Scheme 3).

**Scheme 4:**
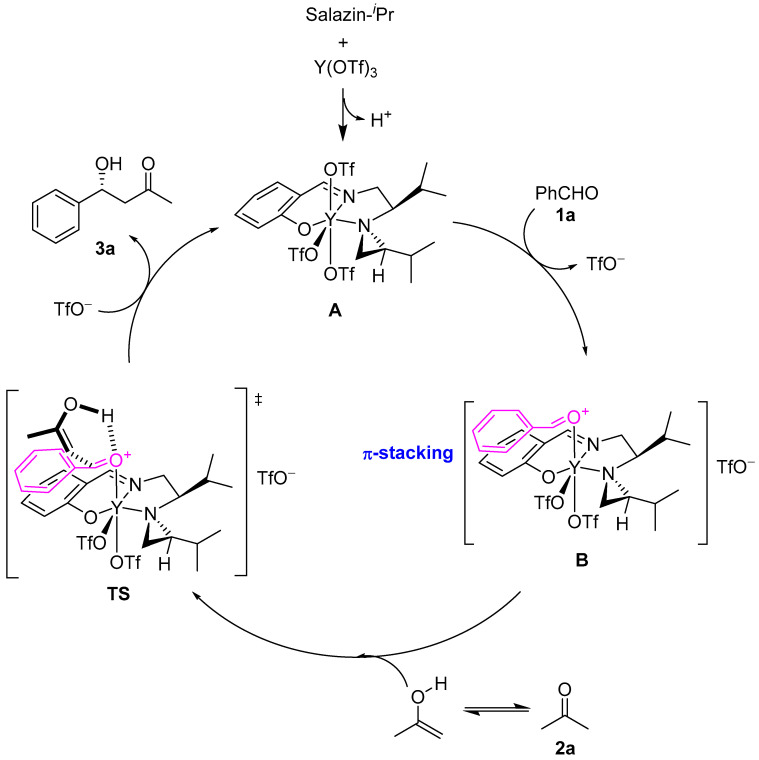
Proposed mechanism for the asymmetric catalytic aldol condensation of benzaldehyde and acetone under the catalysis of Y(OTf)_3_-tridentate ligand complex. ^‡^ Indicates that the structure is transition state.

On the basis of the reported coordination of Y(OTf)_3_ [29] and the absolute configuration of adducts **3**, the plausible mechanism of the catalytic asymmetric reaction is proposed as follows. Tridentate ligand salazin-*^i^*Pr first coordinates with Y(OTf)_3_ to form the complex **A**. Benzaldehyde (**1a**) coordinates with the central metal Y from the top side of the catalyst by exchange with triflate, generating a new complex **B**. The enolic form of acetone is generated under the acid catalysis and attacks the coordinated benzaldehyde in the complex **B** from the top side of benzaldehyde through a hydrogen bond-bound six-membered ring transition state **TS**. After the nucleophilic attack, adduct **3a** is generated and undergoes an exchange with triflate to regenerate the complex **A** for the next catalytic cycle through the release of product **3a** (Scheme 4). Similarly, benzaldehyde (**1a**) can coordinate with the central metal Y from the bottom side, as well. In this case, the enolic form of acetone attacks the coordinated benzaldehyde (**1a**) from its bottom side, affording adduct **3a** through a similar six-membered ring transition state under the backbone of the tridentate ligand. For the both top and bottom coordination directions of benzaldehyde (**1a**), it should be located on the left side due to the steric hindrance of the isopropyl group on the right side and favorable π-stacking interaction with the benzene ring in the chiral ligand backbone. The π-stacking interaction is verified by the fact that both aliphatic cyclohexanecarbaldehyde and non-electron-deficient benzaldehydes did not take place in the catalytic asymmetric aldol condensation due to the absence of or weak π-stacking interaction. Because the benzene ring in the chiral ligand backbone is an electron-rich aromatic ring due to its strong electron-donating hydroxyl group, it would exert strong interaction with electron-deficient aromatic aldehydes, forming favorable transition states in the catalytic cycle.

The π-stacking interaction exerts a crucial influence in promoting organic reactions [34] and controlling stereoselectivities in asymmetric organic reactions [35,36,37]. In the current asymmetric reaction, it also plays an important role in controlling the enantioselectivities and diastereoselectivities.

## 3. Materials and Methods

### 3.1. Materials and Instruments

Unless otherwise noted, all materials were purchased from commercial suppliers. ^1^H NMR spectra were recorded on a Bruker 400 NMR spectrometer (Billerica, MA, USA), usually with TMS as an internal standard. Chemical shifts are recorded in ppm relative to tetramethylsilane and with the solvent resonance as the internal standard. Data are reported as follows: chemical shift, multiplicity—singlet (s), doublet (d), triplet (t), quartet (q), double doublet (dd), multiplet (m), and broad (br)—coupling constants (Hz), and integration. ^13^C NMR data were collected on the same instrument (101 MHz), with complete proton decoupling. Unless otherwise noted, all the solvents were purified using usual methods prior to use. Column chromatography was performed on silica gel (normal phase, 200–300 mesh) from Anhui Liangchen Silicon Material Co., Ltd. (Lu’an, China). Petroleum ether (PE, b.p. 60–90 °C) and ethyl acetate (EA) were used as eluent. Reactions were monitored using thin-layer chromatography (TLC) on GF254 silica gel plates (0.2 mm) from Anhui Liangchen Silicon Material Co., Ltd. The plates were visualized using UV light. Specific rotations were measured on an Anton Paar MCP500 polarimeter (Singapore) and reported as follows: [α]_D_^T^ (c: g/100 mL, in solvent). The enantiomeric excesses were determined using chiral HPLC analysis using an Agilent 1260 LC instrument (Santa Clara, CA, USA) with Daicel Chiralcel OD-H, AD-H, OJ-H, or AS-H column (Hyderabad, India) with a mixture of isopropyl alcohol and hexane as eluents.

The chiral aziridine-containing vicinal iminophenol tridentate ligands (salazins) were prepared from enantiopure aziridines and salicylaldehydes by referring our previously reported procedure [26].

### 3.2. General Procedure for the Asymmetric Catalytic Aldol Condensation of Aromatic Aldehydes ***1*** and Ketones ***2***

The chiral tridentate salazin ligand (0.02 mmol) and rare-earth metal triflate (0.02 mmol) were stirred in 2 mL of ketone **2** at 40 °C for an hour. After the reaction mixture was cooled to room temperature, aldehyde **1** (0.2 mmol) was added into the mixture. The mixture was stirred at 0 °C for 26 h. The reaction mixture was quenched with 1 mL of saturated aqueous ammonium chloride solution and then extracted with DCM (3 × 5 mL). The combined organic layer was dried over anhydrous Na_2_SO_4_. After the evaporation of the solvent under reduced pressure, the crude residue was purified using column chromatography on silica gel using petroleum ether/ethyl acetate (3:1, *v*/*v*) as eluent to afford the pure aldol adduct **3** or **5**. The enantiomeric excess was determined using chiral HPLC analysis with a mixture of *^i^*PrOH and hexane as eluent.

*(R)-4-Hydroxy-4-(4-nitrophenyl)butan-2-one* (**3a**). Colorless crystals, 41 mg, yield 98%; m.p. 58–60 °C Lit. [26] m.p. 58–60 °C; ${\left[\alpha \right]}_{D}^{25}$ = +63.1 (c 0.50, CHCl_3_), Lit. [26] ${\left[\alpha \right]}_{D}^{25}$ = +37.0 (c 0.54, CHCl_3_); R*_f_* = 0.35 (PE/EA = 3:1, *v*/*v*), ee 98%, HPLC analysis: chiralpak AS-H (*i*-PrOH/hexane = 25:75, *v*/*v*, 1.0 mL/min, 260 nm) major *t*_R_ = 17.10 min and minor *t*_R_ = 24.08 min. ^1^H NMR (400 MHz, CDCl_3_) δ 8.20 (d, *J* = 8.8 Hz, 2H), 7.54 (d, *J* = 8.8 Hz 2H), 5.30–5.24 (m, 1H), 3.68 (d, *J* = 3.0 Hz, 1H), 2.87 (s, 1H), 2.86 (d, *J* = 2.7 Hz, 1H), 2.23 (s, 3H). ^13^C NMR (101 MHz, CHCl_3_) δ 208.5, 150.0, 147.23, 126.4, 123.7, 68.8, 51.5, 30.7.

*(R)-4-Hydroxy-4-(3-nitrophenyl)butan-2-one* (**3b**). Colorless oil, 30 mg, yield 72%, ${\left[\alpha \right]}_{D}^{25}$ = +65.8 (c 0.54, CHCl_3_), Lit. [26] ${\left[\alpha \right]}_{D}^{25}$ = +65.8 (c 0.60, CHCl_3_); R*_f_* = 0.33 (PE/EA = 3:1, *v*/*v*), ee 90%. HPLC analysis: chiralpak AD-H (*i*-PrOH/hexane = 10:90, *v*/*v,* 1.0 mL/min, 260 nm) major *t*_R_ = 14.76 min and minor *t*_R_ = 15.51 min. ^1^H NMR (400 MHz, CDCl_3_) δ 8.24 (s, 1H), 8.13 (d, *J* = 8.2 Hz, 1H), 7.72 (d, *J* = 7.7 Hz, 1H), 7.53 (t, *J* = 7.9 Hz, 1H), 5.30–5.23 (m, 1H), 3.72 (d, *J* = 3.1 Hz, 1H), 2.90 (s, 1H), 2.89 (s, 1H), 2.23 (s, 3H). ^13^C NMR (101 MHz, CDCl_3_) δ 208.5, 148.3, 144.9, 131.8, 129.4, 122.5, 120.7, 68.7, 51.5, 30.7.

*(R)-4-Hydroxy-4-(2-nitrophenyl)butan-2-one* (**3c**). Yellow oil, 35 mg, yield 84%; ${\left[\alpha \right]}_{D}^{25}$ = −157.6 (c 0.30, CHCl_3_), Lit. [26] ${\left[\alpha \right]}_{D}^{25}$ = −142.6 (c 0.78, CHCl_3_); R*_f_* = 0.31 (PE/EA = 3:1, *v*/*v*), ee 93%. HPLC analysis: chiralpak AD-H (*i*-PrOH/hexane = 5:95, *v*/*v*, 1.0 mL/min, 260 nm) major *t*_R_ = 22.13 min and minor *t*_R_ = 23.18 min. ^1^H NMR (400 MHz, CDCl_3_) δ 7.96 (dd, *J* = 8.2, 1.3 Hz, 1H), 7.90 (dd, *J* = 8.0, 1.4 Hz, 1H), 7.67 (td, *J* = 7.6, 1.3 Hz, 1H), 7.44 (ddd, *J* = 8.5, 7.4, 1.5 Hz, 1H), 5.68 (dt, *J* = 9.5, 2.4 Hz, 1H), 3.76 (d, *J* = 3.0 Hz, 1H), 3.13 (dd, *J* = 17.8, 2.1 Hz, 1H), 2.73 (dd, *J* = 17.8, 9.4 Hz, 1H), 2.24 (s, 3H). ^13^C NMR (101 MHz, CDCl_3_) δ 208.8, 147.1, 138.4, 133.8, 128.3, 128.2, 124.4, 65.6, 51.0, 30.4.

*(R)-4-Hydroxy-4-(4-cyanophenyl)butan-2-one* (**3d**). Yellow oil, 35 mg, yield 93%; ${\left[\alpha \right]}_{D}^{25}$ = +74.3 (c 0.48, CHCl_3_), Lit. [26] ${\left[\alpha \right]}_{D}^{25}$ = +81.1 (c 0.62, CHCl_3_); R*_f_* = 0.33 (PE/EA = 3:1, *v*/*v*), ee 91%. HPLC analysis: chiralpak AS-H (*i*-PrOH/hexane = 30:70, *v*/*v*, 1.0 mL/min, 214 nm) major *t*_R_ = 12.17 min and minor *t*_R_ = 22.29 min. ^1^H NMR (400 MHz, CDCl_3_) δ 7.64 (d, *J* = 8.2 Hz, 2H), 7.48 (d, *J* = 8.3 Hz, 2H), 5.21 (td, *J* = 6.2, 3.2 Hz, 1H), 3.65 (dt, *J* = 3.4, 1.6 Hz, 1H), 2.84 (d, *J* = 6.2 Hz, 2H), 2.21 (s, 3H). ^13^C NMR (101 MHz, CDCl_3_) δ 208.4, 148.0, 132.3, 126.3, 118.7, 111.2, 69.0, 51.5, 30.7.

*(R)-4-(4-Chlorophenyl)-4-hydroxybutan-2-one* (**3e**). Colorless crystals, 13 mg, yield 33%; m.p. 52–54 °C, Lit. [26] m.p. 52–54 °C; ${\left[\alpha \right]}_{D}^{25}$ = +70.5 (c 0.50, CHCl_3_), Lit. [26] ${\left[\alpha \right]}_{D}^{25}$ = +62.0 (c 0.48, CHCl_3_); R*_f_* = 0.33 (PE/EA = 3:1, *v*/*v*), ee 91%. HPLC analysis: chiralpak AS-H (*i*-PrOH/hexane = 15:85, *v*/*v*, 1.0 mL/min, 214 nm) major *t*_R_ = 11.36 min and minor *t*_R_ = 14.18 min. ^1^H NMR (400 MHz, CDCl_3_) δ 7.29 (d, *J* = 8.7 Hz, 2H), 7.26 (d, *J* = 8.7 Hz, 2H), 5.09 (dt, *J* = 9.1, 3.0 Hz, 1H), 3.64–3.59 (m, 1H), 2.83 (dd, *J* = 17.5, 8.9 Hz, 1H), 2.75 (dd, *J* = 17.5, 3.6 Hz, 1H), 2.17 (s, 3H). ^13^C NMR (101 MHz, CDCl_3_) δ 208.7, 141.3, 133.1, 128.5, 126.9, 69.0, 51.7, 30.6.

*(R)-4-(4-Bromophenyl)-4-hydroxybutan-2-one* (**3f**). Colorless crystals, 19 mg, yield 39%; m.p. 57–59 °C, Lit. [26] m.p. 57–59 °C; ${\left[\alpha \right]}_{D}^{25}$ = +54.5 (c 0.51, CHCl_3_), Lit. [26] ${\left[\alpha \right]}_{D}^{25}$ = +42.2 (c 0.46, CHCl_3_), R*_f_* = 0.40 (PE/EA = 3:1, *v*/*v*), ee 88%. HPLC analysis: chiralpak AS-H (*i*-PrOH/hexane = 20:80, *v*/*v*, 1.0 mL/min, 214 nm) major *t*_R_ = 9.33 min and minor *t*_R_ = 11.66 min. ^1^H NMR (400 MHz, CDCl_3_) δ 7.46 (d, *J* = 8.4 Hz, 2H), 7.22 (d, *J* = 8.5 Hz, 2H), 5.09 (dq, *J* = 8.8, 2.8 Hz, 1H), 3.51–3.45 (m, 1H), 2.87–2.74 (m, 2H), 2.18 (s, 3H). ^13^C NMR (101 MHz, CDCl_3_) δ 208.7, 141.3, 133.1, 128.5, 126.9, 69.0, 51.7, 30.6.

*(R)-4-Hydroxy-4-(naphthalen-1-yl)butan-2-one* (**3j**). Colorless crystals, 15 mg, yield 35%; m.p. 99–101 °C; [α]^24^_D_ = +63.0 (c 0.54, CHCl_3_), Lit. [38] ${\left[\alpha \right]}_{D}^{25}$ = +74.3 (c 0.48, CHCl_3_), R*_f_* = 0.30 (PE/EA = 3:1, *v*/*v*), ee 96%. HPLC analysis: chiralpak AS-H (*i*-PrOH/hexane = 15:85, *v*/*v*, 1.0 mL/min, 210 nm) major *t*_R_ = 16.91 min and minor *t*_R_ = 16.22 min. ^1^H NMR (400 MHz, CDCl_3_) δ 8.00–7.96 (m, 1H), 7.87–7.83 (m, 1H), 7.76 (dt, *J* = 8.3, 1.1 Hz, 1H), 7.66 (dt, *J* = 7.1, 1.0 Hz, 1H), 7.52–7.42 (m, *J* = 11.6, 8.2, 7.0, 3.3 Hz, 3H), 5.92 (td, *J* = 6.1, 2.9 Hz, 1H), 3.48 (d, *J* = 3.3 Hz, 1H), 2.95 (d, *J* = 6.1 Hz, 2H), 2.19 (s, 3H). ^13^C NMR (101 MHz, CDCl_3_) δ 209.1, 138.2, 133.7, 129.8, 128.9, 128.0, 126.1, 125.5, 122.9, 122.7, 66.6, 51.3, 30.7.

*(R)-4-Hydroxy-4-(naphthalen-2-yl)butan-2-one* (**3k**). Yellow oil, 36 mg, yield 84%, ${\left[\alpha \right]}_{D}^{24}$ = +44.7 (c 0.40, CHCl_3_), R*_f_* = 0.60 (PE/EA = 2:1, *v*/*v*), ee 91%, Lit. [26] ${\left[\alpha \right]}_{D}^{25}$ = +39.8 (c 0.45, CHCl_3_). HPLC analysis: chiralpak AS-H (*i*-PrOH/hexane = 15:85, *v*/*v*, 1.0 mL/min, 210 nm) major *t*_R_ = 15.00 min and minor *t*_R_ = 16.91 min. ^1^H NMR (400 MHz, CDCl_3_) δ 7.83–7.72 (m, 4H), 7.47–7.37 (m, 3H), 5.25 (dd, *J* = 9.2, 3.3 Hz, 1H), 3.61 (br s, 1H), 2.89 (dd, *J* = 17.4, 9.2 Hz, 1H), 2.79 (dd, *J* = 17.3, 3.4 Hz, 1H), 2.12 (s, 3H). ^13^C NMR (101 MHz, CDCl_3_) δ 208.9, 140.1, 133.1, 132.8, 128.2, 127.9, 127.5, 126.1, 125.8, 124.2, 123.7, 69.8, 51.8, 30.6.

*(R)-4-Hydroxy-4-(quinolin-4-yl)butan-2-one* (**3l**). Colorless crystals, 38 mg, yield 89%; m.p. 62–64 °C, ${\left[\alpha \right]}_{D}^{25}$ = +168.3 (c 0.75, CHCl_3_), Lit. [39] [α]^20^_D_ = +37.2 (c 0.1, CHCl_3_); R*_f_* = 0.20 (PE/EA = 1:1, *v*/*v*), ee 89%. HPLC analysis: chiralpak OD-H (*i*-PrOH/hexane = 20:80, *v*/*v*, 1.0 mL/min, 260 nm) minor *t*_R_ = 10.08 min and major *t*_R_ = 10.80 min. ^1^H NMR (400 MHz, CDCl_3_) δ 8.80 (d, *J* = 4.5 Hz, 1H), 8.08 (d, *J* = 7.9 Hz, 1H), 7.90 (d, *J* = 8.4 Hz, 1H), 7.66 (ddd, *J* = 8.4, 6.8, 1.4 Hz, 1H), 7.62 (dd, *J* = 4.5, 0.8 Hz, 1H), 7.52 (ddd, *J* = 8.3, 6.9, 1.3 Hz, 1H), 5.97 (t, *J* = 6.0 Hz, 1H), 4.71 (br s, 1H), 2.92 (d, *J* = 6.0 Hz, 2H), 2.23 (s, 3H). ^13^C NMR (101 MHz, CDCl_3_) δ 207.9, 150.2, 148.8, 147.8, 130.1, 129.1, 126.7, 124.9, 122.5, 117.5, 65.4, 51.1, 30.8.

*(R)-4-Hydroxy-4-(4-(methylsulfonyl)phenyl)butan-2-one* (**3m**). Colorless crystals, 41 mg, yield 86%, m.p. 113–115 °C, ${\left[\alpha \right]}_{D}^{25}$ = +57.2 (c 0.37, CHCl_3_), R*_f_* = 0.15 (PE/EA = 1:1, *v*/*v*), ee 92%. HPLC analysis: chiralpak AS-H (*i*-PrOH/hexane = 40:60, *v*/*v*, 1.0 mL/min, 214 nm) major *t*_R_ = 22.36 min and minor *t*_R_ = 35.43 min. ^1^H NMR (400 MHz, CDCl_3_) δ 7.87 (d, *J* = 8.4 Hz, 2H), 7.57 (d, *J* = 8.3 Hz, 2H), 5.24 (dd, *J* = 7.4, 5.0 Hz, 1H), 3.81 (s, 1H), 3.04 (s, 3H), 2.86 (d, *J* = 2.9 Hz, 1H), 2.85 (s, 1H), 2.21 (s, 3H). ^13^C NMR (101 MHz, CDCl_3_) δ 208.4, 149.1, 139.4, 127.5, 126.5, 68.9, 51.5, 44.4, 30.6. HRMS (ESI): *m*/*z* calcd for C_11_H_14_NaO_4_S^+^ [M+Na]^+^: 265.0505, found: 265.0513.

*(E)-4-(4-(Methylsulfonyl)phenyl)but-3-en-2-one* (**4m**). Colorless crystals, 6 mg, yield 14%, m.p. 125–126 °C, Lit. [40] m.p. 124–125 °C. R*_f_* = 0.40 (PE/EA = 3:1, *v*/*v*). ^1^H NMR (400 MHz, CDCl_3_) δ 8.00–7.95 (m, 2H), 7.76–7.71 (m, 2H), 7.53 (d, *J* = 16.3 Hz, 1H), 6.82 (d, *J* = 16.3 Hz, 1H), 3.08 (s, 3H), 2.42 (s, 3H). ^13^C NMR (101 MHz, CDCl_3_) δ 197.6, 141.6, 140.5, 139.8, 130.0, 128.8, 128.1, 44.4, 28.0.

*(R)-4-Hydroxy-4-(4-(4,4,5,5-tetramethyl-1,3,2-dioxaborolan-2-yl)phenyl)butan-2-one* (**3n**). Colorless crystals, 41 mg, yield 70%, m.p. 58–60 °C, ${\left[\alpha \right]}_{D}^{25}$ = +52.2 (c 0.37, CHCl_3_), R*_f_* = 0.40 (PE/EA = 3:1, *v*/*v*), ee 87%. HPLC analysis: chiralpak AD-H (*i*-PrOH/hexane = 10:90, *v*/*v*, 1.0 mL/min, 230 nm) major *t*_R_ = 7.18 min and minor *t*_R_ = 8.24 min. ^1^H NMR (400 MHz, CDCl_3_) δ 7.79 (d, *J* = 8.1 Hz, 2H), 7.35 (d, *J* = 7.9 Hz, 2H), 5.16 (dt, *J* = 8.9, 3.1 Hz, 1H), 3.36 (d, *J* = 3.1 Hz, 1H), 2.86 (dd, *J* = 17.5, 8.9 Hz, 1H), 2.79 (dd, *J* = 17.5, 3.5 Hz, 1H), 2.18 (s, 3H), 1.34 (s, 12H). ^13^C NMR (101 MHz, CDCl_3_) δ 208.9, 145.8, 135.0 (2C), 124.8, 83.8, 69.8, 51.9, 30.7, 24.8. HRMS (ESI): *m*/*z* calcd for C_16_H_23_BNaO_4_^+^ [M+Na]^+^: 313.1582, found: 313.1585.

*(S/R)-2-((R/S)-Hydroxy(4-nitrophenyl)methyl)cyclobutan-1-one* (**5a**). Mixture of *anti*- and *syn*-diastereomers, yellow solid, 11 mg, yield 25%; m.p. 98–101 °C, Lit. [41] m.p. of *syn*-diastereomer 101–103 °C, m.p. of *anti*-diastereomer 97–99 °C; ${\left[\alpha \right]}_{D}^{25}$ = +19.0 (c 0.27, CHCl_3_); R*_f_* = 0.25 (PE/EA =3:1, *v*/*v*), dr *anti*:*syn* = 53:47, ee of *anti* 83%, ee of *syn* 69%. HPLC analysis: chiralpak AS-H (*i*-PrOH/hexane = 10:90, *v*/*v*, 1.0 mL/min, 260 nm) *syn*-diastereomer: minor *t*_R_ = 20.96 and major *t*_R_ = 24.21 min. *anti*-diastereomer: major *t*_R_ = 28.10 min and minor *t*_R_ = 52.43 min. ^1^H NMR (400 MHz, CDCl_3_) δ 8.29–8.18 (m, 2H), 7.61–7.48 (m, 2H), 5.34–4.94 (m, 1H), 3.67–3.57 (m, 1H), 3.19–3.08 (m, 1H), 3.04–2.95 (m, 1H), 2.88–2.84 (m, 1H), 2.26–2.06 (m, 1H), 1.99–1.88 (m, 1H). ^13^C NMR (101 MHz, CDCl_3_) δ 209.2, 208.5, 149.9, 148.3, 127.0, 126.4, 123.8, 123.8, 73.0, 68.9, 66.0, 51.5, 45.2, 30.7, 14.1.

*(S)-2-(Hydroxy(4-nitrophenyl)methyl)cyclopentan-1-one* (**5b**). Mixture of *anti*- and *syn*-diastereomers, yellow crystals, 38 mg, yield 81%; m.p. 89–91 °C, Lit. [42] m.p. 89–91 °C; ${\left[\alpha \right]}_{D}^{25}$ = +124.0 (c 0.38, CHCl_3_), Lit. [43] ${\left[\alpha \right]}_{D}^{25}$ = +98 (c 1.5, CHCl_3_); R*_f_* = 0.28 (PE/EA = 3:1, *v*/*v*), dr *anti*:*syn* = 83:17, ee of *anti* 96%. HPLC analysis: chiralpak AS-H (*i*-PrOH/hexane = 10:90, *v*/*v*, 1.0 mL/min, 210 nm) *syn*-diastereomer: *t*_R_ = 36.39 min (enantiomers are inseparable); *anti*-diastereomer minor *t*_R_ = 41.78 min and major *t*_R_ = 59.270 min. ^1^H NMR (400 MHz, CDCl_3_) δ 8.23–8.18 (m, 2H), 7.54 (d, *J* = 8.4 Hz, 2H, *anti*), 7.54 (d, *J* = 9.2 Hz, 2H, *syn*), 5.42 (d, *J* = 2.8 Hz, 1H, *syn*), 4.86 (d, *J* = 9.2 Hz, 1H, *anti*), 4.77 (br s, 1H, *anti*), 4.38–4.33 (m, 1H, *syn*), 2.48–2.35 (m, 2H), 2.38–2.17 (m, 1H), 2.07–1.97 (m, 1H), 1.73–1.68 (m, 2H), 1.58–1.48 (m, 1H). ^13^C NMR (101 MHz, CDCl_3_) δ 222.2, 148.6, 147.7, 127.3, 123.6, 74.3, 55.0, 38.5, 26.8, 20.3.

*(S)-2-((R)-Hydroxy(4-nitrophenyl)methyl)cyclohexan-1-one* (**5c**). Colorless crystals, 47 mg, yield 94%; m.p. 129–130 °C, Lit. [42] m.p. 129–130 °C; ${\left[\alpha \right]}_{D}^{25}$ = +11.1 (c 0.17, CHCl_3_), Lit. [42] ${\left[\alpha \right]}_{D}^{25}$ = +12.8 (c 1.85, CHCl_3_); R*_f_* = 0.30 (PE/EA = 3:1, *v*/*v*), dr *anti*:*syn* = 94:6, ee of *anti* 91%, ee of *syn* 5%. HPLC analysis: chiralpak AS-H (*i*-PrOH/hexane = 15:85, *v*/*v*, 1.0 mL/min, 210 nm) *syn*-diastereomer: major *t*_R_ = 14.62 min and minor *t*_R_ = 15.57 min; *anti*-diastereomer: minor *t*_R_ = 16.92 min and major *t*_R_ = 22.93 min. ^1^H NMR (400 MHz, CDCl_3_) δ 8.20 (d, *J* = 8.7 Hz, 2H), 7.51 (d, *J* = 8.7 Hz, 2H), 4.91 (dd, *J* = 8.3, 2.2 Hz, 1H), 4.11 (d, *J* = 3.1 Hz, 1H), 2.67–2.58 (m, 1H), 2.53–2.46 (m, 1H), 2.37 (td, 13.2, 6.0 Hz, 1H), 2.16–2.08 (m, 1H), 1.87–1.79 (m, 1H), 1.73–1.65 (m, 1H), 1.63–1.52 (m, 2H), 1.40 (td, *J* = 13.2, 4.0 Hz, 1H). ^13^C NMR (101 MHz, CDCl_3_) δ 214.7, 148.4, 147.5, 127.8, 123.5, 73.9, 57.1, 42.6, 30.7, 27.6, 24.6.

*(1R,2S)-1-Hydroxy-2-methyl-1-(4-nitrophenyl)pentan-3-one* (**5d**). Colorless oil, 14 mg, yield 30%; ${\left[\alpha \right]}_{D}^{25}$ = +27.3 (c 0.08, CHCl_3_), Lit. [43] ${\left[\alpha \right]}_{D}^{25}$ = +26 (c 1.0, CHCl_3_),R*_f_* = 0.53 (PE/EA = 3:1, *v*/*v*), dr *anti*:*syn* = 86:14, ee of *anti* 98%, ee of *syn* 71%. HPLC analysis: chiralpak OJ-H (*i*-PrOH/hexane = 10:90, *v*/*v*, 1.0 mL/min, 260 nm) *syn*-diastereomer major *t*_R_ = 14.92 min and minor *t*_R_ = 16.25 min; *anti*-diastereomer major *t*_R_ = 13.44 min and minor *t*_R_ = 17.29 min. ^1^H NMR (400 MHz, CDCl_3_) δ 8.21 (d, *J* = 8.8 Hz, 2H), 7.52 (d, *J* = 8.6 Hz, 2H), 5.32–4.81 (m, 1H), 3.58 (br s, 1H), 2.99–2.79 (m, 1H), 2.61 (dq, *J* = 18.2, 7.2 Hz, 1H), 2.46 (dq, *J* = 18.1, 7.2 Hz, 1H), 1.07 (d, *J* = 7.2 Hz, 3H), 1.04 (d, *J* = 7.2 Hz, 3H). ^13^C NMR (101 MHz, CDCl_3_) δ 216.2, 149.1, 127.3, 126.8, 124.3, 72.0, 51.4, 35.1, 9.9, 7.5.

*(R)-3-Hydroxy-3-(4-nitrophenyl)-1-phenylpropan-1-one* (**5e**). Colorless crystals, 8 mg, yield 15%; m.p. 112–113 °C, Lit. [44] m.p. 113–114 °C; [α]^24^_D_ = +52.3 (c 1.0, CHCl_3_), Lit. [45] ${\left[\alpha \right]}_{D}^{25}$ = +13.7 (c 0.06, CHCl_3_), R*_f_* = 0.52 (PE/EA = 3:1, *v*/*v*), ee 44%. HPLC analysis: chiralpak AD-H (*i*-PrOH/hexane = 10:90, *v*/*v*, 1.0 mL/min, 254 nm), minor *t*_R_ = 31.19 min and major *t*_R_ = 39.00 min. ^1^H NMR (400 MHz, CDCl_3_) δ 8.21 (d, *J* = 8.8 Hz, 2H), 7.94 (d, *J* = 7.1 Hz, 2H), 7.61 (d, *J* = 8.7 Hz, 3H), 7.48 (t, *J* = 7.8 Hz, 2H), 5.46 (dt, *J* = 8.4, 3.4 Hz, 1H), 3.91 (d, *J* = 3.1 Hz, 1H), 3.41 (dd, *J* = 17.9, 3.6 Hz, 1H), 3.35 (dd, *J* = 17.9, 8.4 Hz, 1H). ^13^C NMR (101 MHz, CDCl_3_) δ 199.4, 150.2, 147.3, 136.1, 134.0, 128.8, 128.1, 126.5, 123.7, 69.1, 46.9.

## 4. Conclusions

The chiral aziridine-containing vicinal iminophenol tridentate ligands (salazins) are a class of easily synthesized chiral ligands from readily available prepared enantiopure aziridines and salicylaldehydes. Both of their scandium and yttrium triflate complexes show excellent enantioselectivity in the catalytic asymmetric aldol condensation of electron-deficient aromatic aldehydes and ketones and both excellent diastereo- and enantioselectivity in the reactions with cycloalkanones. The stereoselectivities are attributed to the strong π–stacking interaction between aromatic aldehydes and the vicinal iminophenol group in the chiral ligands.

## Figures and Tables

**Table 1:** Catalytic asymmetric aldol condensation in different solvents ^a^.

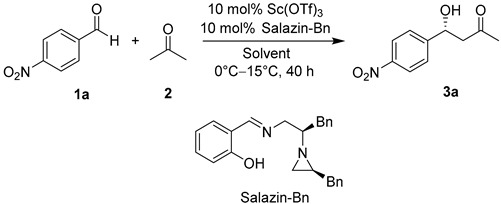			
Entry	Solvent	Yield/%	ee/%
1	PhMe	3	38
2	EtOH	98	53
3	DCM	10	60
4	THF	20	49
5	acetone	96	87

^a^ All the reactions were carried out with 4-nitrobenzaldehyde (31 mg, 0.2 mmol, 1 eq.) and acetone (12 mg, 0.2 mmol, 1 eq.) in 2 mL of solvent in a 10 mL reaction tube under the catalysis of chiral ligand salazin-Bn (7.4 mg, 0.02 mmol, 0.1 eq.) and Sc(OTf)_3_ (9.8 mg, 0.02 mol, 0.1 eq.) under stirring for 40 h. The yields were obtained using chromatography on silica gel.

**Table 2:** Catalytic asymmetric aldol condensation under the catalysis of different ligands and rare-earth metal triflates at different reaction temperatures ^a^.

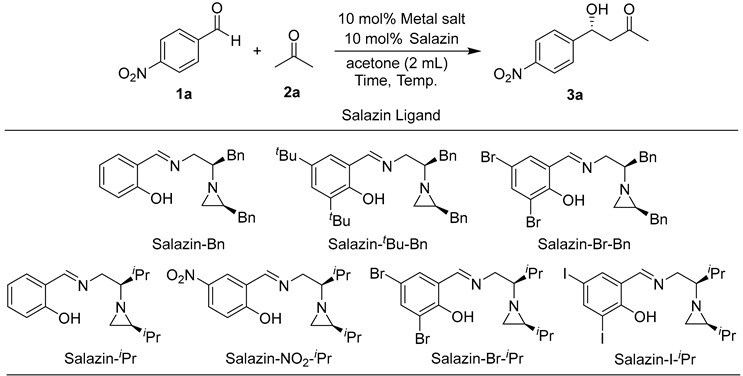						
Entry	Metal Salt	Ligand	Temp./°C	Time/h	Yield/%	ee/%
1	Sc(OTf)_3_	Salazin-Bn	15	40	96	81
2	Sc(OTf)_3_	Salazin-*^t^*Bu-Bn	15	40	96	70
3	Sc(OTf)_3_	Salazin-Br-Bn	15	40	55	78
4	Sc(OTf)_3_	Salazin-NO_2_-*^i^*Pr	15	40	91	86
5	Sc(OTf)_3_	Salazin-Br-*^i^*Pr	15	40	89	87
6	Sc(OTf)_3_	Salazin-I-*^i^*Pr	15	40	60	80
**7**	**Sc(OTf)_3_**	**Salazin-*^i^*Pr**	**15**	**26**	**98**	**88**
**8**	**Y(OTf)_3_**	**Salazin-*^i^*Pr**	**15**	**26**	**98**	**88**
9	La(OTf)_3_	Salazin-*^i^*Pr	15	26	93	83
10	Ce(OTf)_3_	Salazin-*^i^*Pr	15	26	91	86
11	Eu(OTf)_3_	Salazin-*^i^*Pr	15	26	98	85
12	Gd(OTf)_3_	Salazin-*^i^*Pr	15	26	98	87
13	Lu(OTf)_3_	Salazin-*^i^*Pr	15	26	96	87
14	Zn(OTf)_2_	Salazin-*^i^*Pr	15	40	90	70
15	Sc(OTf)_3_	Salazin-*^i^*Pr	40	20	98	85
16	Sc(OTf)_3_	Salazin-*^i^*Pr	20	26	98	88
**17**	**Sc(OTf)_3_**	**Salazin-*^i^*Pr**	**0**	**30**	**98**	**98**
18	Y(OTf)_3_	Salazin-*^i^*Pr	40	20	96	89
19	Y(OTf)_3_	Salazin-*^i^*Pr	20	26	98	88
**20**	**Y(OTf)_3_**	**Salazin-*^i^*Pr**	**0**	**30**	**98**	**98**

^a^ Reactions were conducted on a 0.2 mmol scale of aldehyde in 2 mL of acetone. The yields were obtained using chromatography on silica gel.

**Table 3:** Catalytic asymmetric aldol condensation of cycloalkanones under the catalysis of Y(OTf)_3_ ^a^.

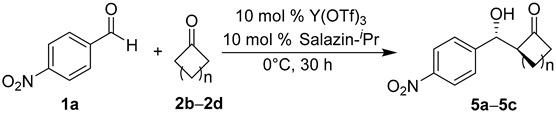						
Entry	Product 5	n	Yield/%	*anti*:*syn*	ee of *anti*/%	ee of *syn*/%
1	**5a**	1	25	53:47	83	69
2	**5b**	2	81	83:17	96	- ^b^
3	**5c**	3	94	94:6	91	5

^a^ Reactions were conducted on a 0.2 mmol scale of 4-nitrobenzaldehyde (**1a**) in 2 mL of cycloalkanone (**2b**–**2d**). The yields were obtained using chromatography on silica gel. ^b^ The *syn*-diastereomer *syn*-**5b** cannot be separated in all of our available chiral columns.

## Data Availability

Data are contained within the article and Supplementary Materials.

## Supplementary Materials

The following supporting information can be downloaded at https://www.mdpi.com/article/10.3390/molecules29091963/s1. Copies of ^1^H-NMR and ^13^C-NMR spectra, HPLC profiles of compounds **3** and **5,** and HRMS spectra of unknown products **3** are included in the Supplementary Materials.

## References

[1] Schmidt F., Keller F., Vedrenne E., Aggarwal V.K. (2009). Stereocontrolled Synthesis of β-Amino Alcohols from Lithiated Aziridines and Boronic Esters. Angew. Chem. Int. Ed. Engl..

[2] Tanner D. (1993). Stereocontrolled synthesis via chiral aziridines. Pure Appl. Chem..

[3] Zhu M., Hu L.B., Chen N., Du D.-M., Xu J.X. (2008). Synthesis of NH-aziridines from vicinal amino alcohols via the Wenker reaction: Scope and limitation. Lett. Org. Chem..

[4] Li X.Y., Chen N., Xu J.X. (2010). An improved and mild Wenker synthesis of aziridines. Synthesis.

[5] Jarzyński S., Utecht G., Leśniak S., Rachwalski M. (2017). Highly enantioselective asymmetric reactions involving zinc ions promoted by chiral aziridine alcohols. Tetrahedron Asymmetry.

[6] Jarzyński S., Leśniak S., Pieczonka A.M., Rachwalski M. (2015). *N*-Trityl-aziridinyl alcohols as highly efficient chiral catalysts in asymmetric additions of organozinc species to aldehydes. Tetrahedron Asymmetry.

[7] Rachwalski M., Jarzyński S., Jasiński M., Leśniak S. (2013). Mandelic acid derived α-aziridinyl alcohols as highly efficient ligands for asymmetric additions of zinc organyls to aldehydes. Tetrahedron Asymmetry.

[8] Pieczonka A.M., Leśniak S., Jarzyński S., Rachwalski M. (2015). Aziridinylethers as highly enantioselective ligands for the asymmetric addition of organozinc species to carbonyl compounds. Tetrahedron Asymmetry.

[9] Pieczonka A.M., Jarzyński S., Wujkowska Z., Leśniak S., Rachwalski M. (2015). Zinc(II) mediated asymmetric aldol condensation catalyzed by chiral aziridine ligands. Tetrahedron Lett..

[10] Jarzyński S., Rachwalski M., Pieczonka A.M., Wujkowska Z., Leśniak S. (2015). Highly efficient conjugate additions of diethylzinc to enones promoted by chiral aziridine alcohols and aziridine ethers. Tetrahedron Asymmetry.

[11] Rachwalski M., Jarzyński S., Leśniak S. (2013). Aziridine ring-containing chiral ligands as highly efficient catalysts in asymmetric synthesis. Tetrahedron Asymmetry.

[12] Adam E.M., Pieczonka M., Rachwalski M., Leśniak S. (2017). Synthesis of chiral 1-(2-aminoalkyl)aziridines via the self-opening reaction of aziridine. ARKIVOC.

[13] Pieczonka A.M., Marciniak L., Rachwalski M., Leśniak S. (2020). Enantiodivergent aldol condensation in the presence of aziridine/acid/water systems. Symmetry.

[14] Pieczonka A.M., Leśniak S., Rachwalski M. (2014). Direct asymmetric aldol condensation catalyzed by aziridine semicarbazide zinc(II) complexes. Tetrahedron Lett..

[15] Leśniak S., Pieczonka A.M., Jarzyński S., Justyna K., Rachwalski M. (2013). Synthesis and evaluation of the catalytic properties of semicarbazides derived from *N*-triphenylmethyl-aziridine-2-carbohydrazides. Tetrahedron Asymmetry.

[16] Buchcic-Szychowska A., Zawisza A., Le’sniak S., Rachwalski M. (2022). Highly efficient asymmetric Morita–Baylis–Hillman reaction promoted by chiral aziridine-phosphines. Catalysts.

[17] Buchcic-Szychowska A., Lésniak S., Rachwalski M. (2022). Chiral aziridine phosphines as highly effective promoters of asymmetric Rauhut–Currier reaction. Symmetry.

[18] Buchcic A., Zawisza A., Lésniak S., Adamczyk J., Pieczonka A.M., Rachwalski M. (2019). Enantioselective Mannich reaction promoted by chiral phosphinoyl-aziridines. Catalysts.

[19] Tanner D., Johansson F., Harden A., Andersson P.G. (1998). A comparative study of C_2_-symmetric bis(aziridine) ligands in some transition metal-mediated asymmetric transformations. Tetrahedron.

[20] Tanner D., Harden A., Johansson F., Wyatt P., Andersson P.G., Zhang S.Y., Zhao S.H., Ciglic M.I., Haugg M., Trabesinger-Rüf N. (1996). Asymmetric catalysis via chiral aziridines. Acta Chem. Scand..

[21] Andersson P.G., Harden A., Tanner D., Norrby P.-O. (1995). Studies of Allylic Substitution Catalysed by a Palladium Complex of a C_2_-Symmetric Bis(aziridine): Preparation and NMR Spectroscopic Investigation of a Chiral π-Allyl Species. Chem.-Eur. J..

[22] Tanner D., Andersson P.G., Harden A., Somfai P. (1994). C_2_-symmetric bis(aziridines): A new class of chiral ligands for transition metal-mediated asymmetric synthesis. Tetrahedron Lett..

[23] Rachwalski M., Leśniak S., Kiełbasiński P. (2010). Highly enantioselective addition of phenylethynylzinc to aldehydes using aziridine-functionalized tridentate sulfinyl ligands. Tetrahedron Asymmetry.

[24] Rachwalski M., Leśniak S., Kiełbasiński P. (2010). Highly enantioselective conjugate addition of diethylzinc to enones using aziridine-functionalized tridentate sulfinyl ligands. Tetrahedron Asymmetry.

[25] Leśniak S., Rachwalski M., Sznajder E., Kiełbasiński P. (2009). New highly efficient aziridine-functionalized tridentate sulfinyl catalysts for enantioselective diethylzinc addition to carbonyl compounds. Tetrahedron Asymmetry.

[26] Chen X.P., Lin C., Du H.G., Xu J.X. (2019). Efficient direct synthesis of aziridine-containing chiral tridentate ligands by the iminium-mediated self-ring opening reaction of enantiopure aziridines and salicylaldehydes. Adv. Synth. Catal..

[27] Ma L.G., Xu J.X. (2004). Nucleophilic ring opening reaction of unsymmetric aziridines and its regioselectivity. Prog. Chem. (Huaxue Jinzhan).

[28] Wu Y.-H., Zhang L.-Y., Wang N.-X., Xing Y.L. (2020). Recent advances in the rare-earth metal triflates catalyzed organic reactions. Catal. Rev..

[29] Feng X.M., Wang Z., Liu X.L. (2017). Chiral Lewis acid rare-earth metal complexes in enantioselective catalysis. Topics in Organometallic Chemistry.

[30] Ma L.G., Jiao P., Zhang Q.H., Du D.-M., Xu J.X. (2007). Ligand and substrate π-stacking interaction contro-lled enantioselectivity in the asymmetric aziridination. Tetrahedron Asymmetry.

[31] Li Z.-Y., Chen Y., Zheng C.-Q., Yin Y., Wang L., Sun X.-Q. (2017). Highly enantioselective aldol reactions catalyzed by reusable upper rim-functionalized calix[4]arene-based l-proline organocatalyst in aqueous conditions. Tetrahedron.

[32] Vlasserou I., Sfetsa M., Gerokonstantis D.-T., Kokotos C.G., Moutevelis-Minakakis P. (2018). Combining prolinamides with 2-pyrrolidinone: Novel organocatalysts for the asymmetric aldol reaction. Tetrahedron.

[33] Chen G., Fu X., Li C., Wu C., Miao Q. (2012). Highly efficient direct a larger-scale aldol reactions catalyzed by a flexible prolinamide based-metal Lewis acid bifunctional catalyst in the presence of water. J. Organomet. Chem..

[34] Wang Y.Q., Chen Y.X., Xu J.X. (2024). π-Stacking-controlled dearomatic sulfur-shifted ene reaction of ketenes and polycyclic arylthiiranes: Access to areno[*d*]-ε-thiolactones. J. Org. Chem..

[35] Li B.N., Wang Y.K., Du D.-M., Xu J.X. (2007). Notable and obvious ketene substituent-dependent effect of temperature on the stereoselectivity in the Staudinger reaction. J. Org. Chem..

[36] Xu J.X. (2024). Recent advances in π-stacking interaction-controlled asymmetric synthesis. Molecules.

[37] Gu L., Yu M., Wu X., Zhang Y., Zhao G. (2006). 4,4′-Disubstituted L-prolines as highly enantioselective catalysts for direct aldol reactions. Adv. Synth. Catal..

[38] Tang Z., Jiang F., Yu L.T., Cui X., Gong L.Z., Mi A.Q., Jiang Y.Z., Wu Y.D. (2003). Novel small organic molecules for a highly enantioselective direct aldol reaction. J. Am. Chem. Soc..

[39] Kucherenko A.S., Kostenko A.A., Gerasimchuk V.V., Zlotin S.G. (2017). Stereospecific diaza-Cope rearrangement as an efficient tool for the synthesis of DPEDA pyridine analogs and related C_2_-symmetric organocatalysts. Org. Biomol. Chem..

[40] Edwards M.L., Ritter H.W., Stemerick D.M., Stewart K.T. (1983). Mannich bases of 4-phenyl-3-buten-2-one. A new class of antiherpes agent. J. Med. Chem..

[41] Cobb A.J.A., Shaw D.M., Longbottom D.A., Gold J.B., Ley S.V. (2005). Organocatalysis with proline derivatives: Improved catalysts for the asymmetric Mannich, nitro-Michael and aldol reactions. Org. Biomol. Chem..

[42] Guo G., Wu Y., Zhao X., Wang J., Zhang L., Cui Y. (2016). Polymerization of L-proline functionalized styrene and its catalytic performance as a supported organocatalyst for direct enantioselective aldol reaction. Tetrahedron Asymmetry.

[43] Da C.-S., Che L.-P., Guo Q.-P., Wu F.-C., Ma X., Jia Y.-N. (2009). 2,4-Dinitrophenol as an effective cocatalyst: Greatly improving the activities and enantioselectivities of primary amine organocatalysts for asymmetric aldol reactions. J. Org. Chem..

[44] Downey C.W., Johnson M.W. (2007). A tandem enol silane formation-Mukaiyama aldol reaction mediated by TMSOTf. Tetrahedron Lett..

[45] Li L., Gou S.H., Liu F. (2014). Highly stereoselective direct aldol reactions catalyzed by a bifunctional chiral diamine. Tetrahedron Asymmetry.

