# Supplementary material for: Y(OTf)3-Salazin-Catalyzed Asymmetric Aldol Condensation
Source: Molecules. 2024 Apr 25;29(9):1963. doi: 10.3390/molecules29091963 (PMC11085936; doi:10.3390/molecules29091963)
Supplement: Supplementary file 1 [file molecules-29-01963-s001.zip › molecules-2970964-supplementary.pdf]

Supporting Information

Y(OTf)$_3$-Salazin-Catalyzed Asymmetric Aldol Condensation

Chengzhuo Wang, Ning Chen, Zhanhui Yang and Jiaxi Xu*

State Key Laboratory of Chemical Resource Engineering, Department of Organic Chemistry, College of Chemistry, Beijing University of Chemical Technology, Beijing 100029, China * Correspondence: jxxu@mail.buct.edu.cn; Tel./Fax: +86-10-6443-5565

Contents

1. **Copies of NMR and HRMS spectra of products……………………………S2**
2. **Copies of HPLC profiles of products………………………………………..S21**

1. Copies of NMR and HRMS spectra of products

(*R*)-4-Hydroxy-4-(4-nitrophenyl)butan-2-one (3a)

^{1}H NMR (400 MHz, $CDCl_3$)

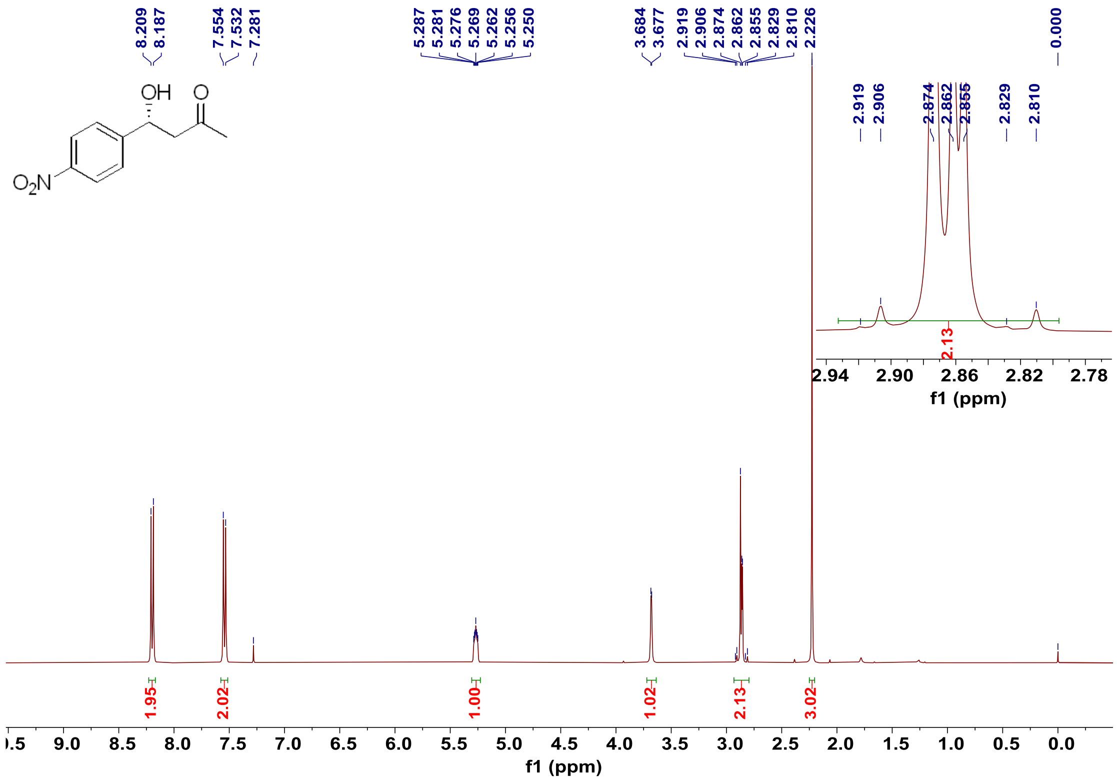

$^{13}C\{^1H\}$ NMR (101 MHz, $CDCl_3$

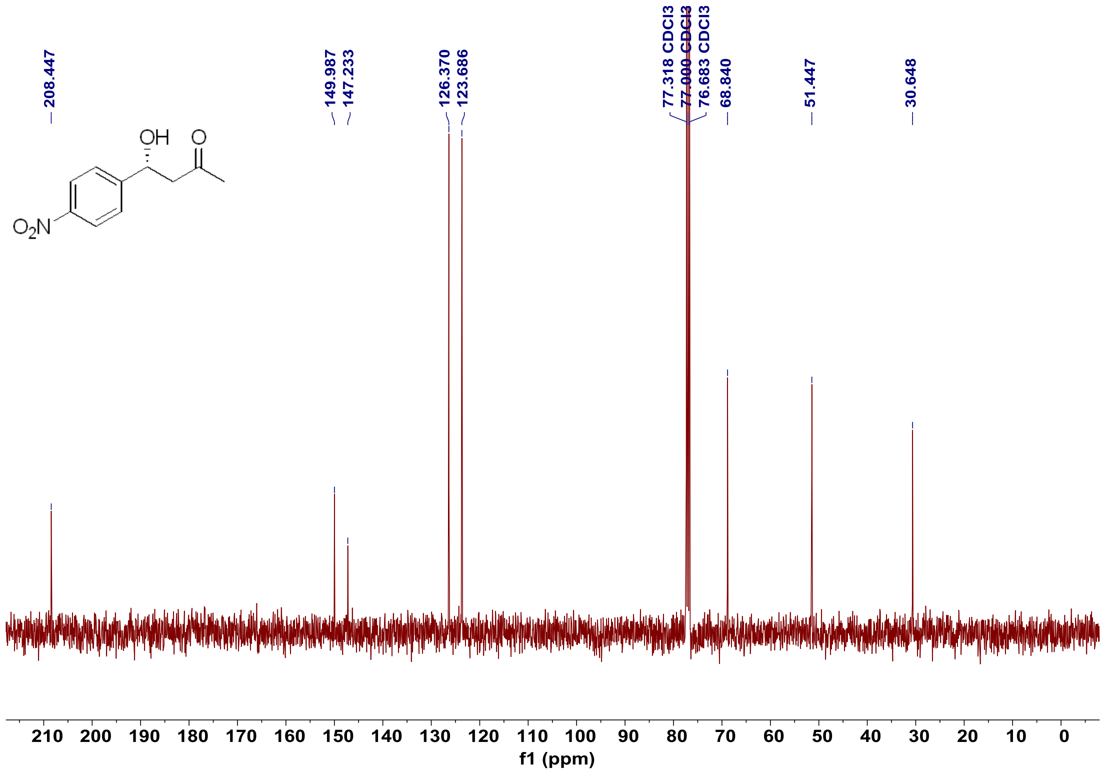

(*R*)-4-Hydroxy-4-(3-nitrophenyl)butan-2-one (3b)

^{1}H NMR (400 MHz, $CDCl_3$)

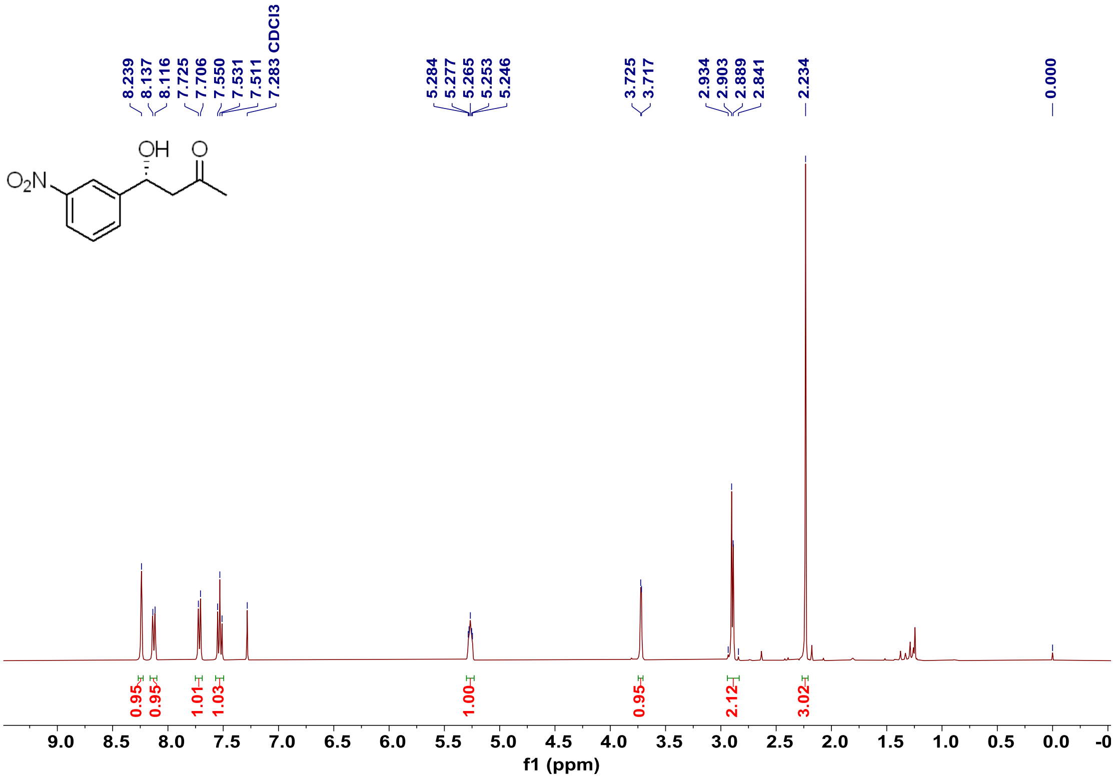

$^{13}C\{^{1}H\}$ NMR (101 MHz, $CDCl_3$)

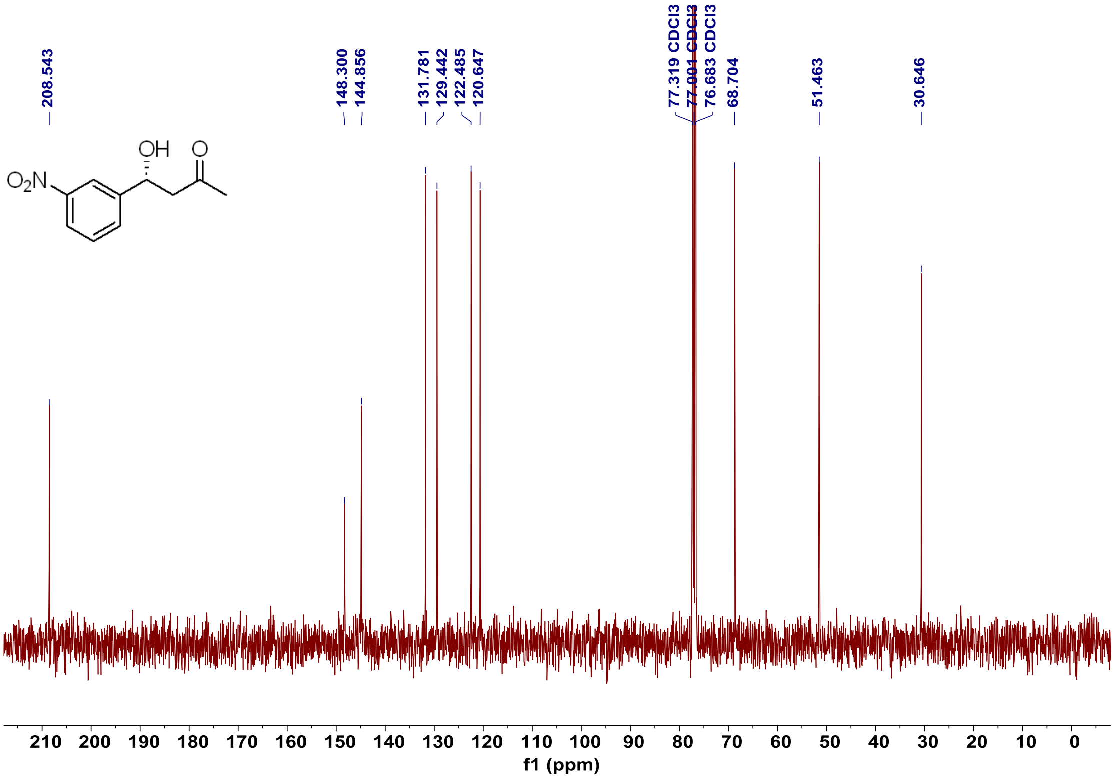

(*R*)-4-Hydroxy-4-(2-nitrophenyl)butan-2-one (3c)

^{1}H NMR (400 MHz, $CDCl_3$)

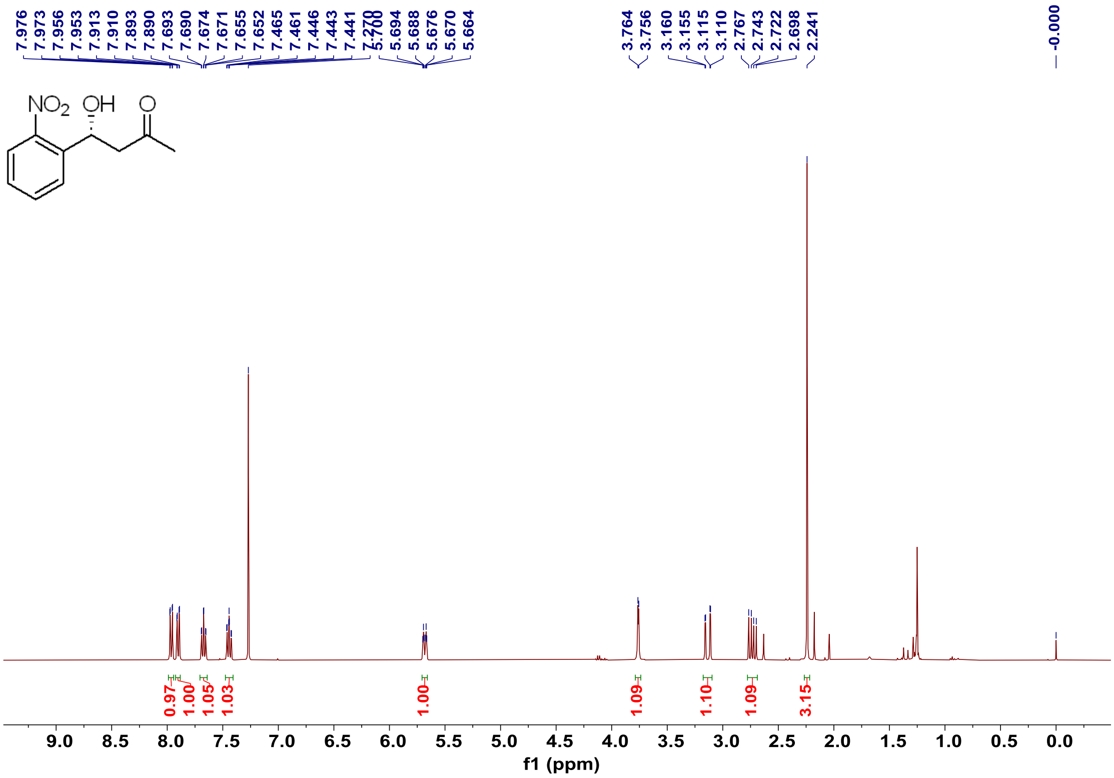

$^{13}C\{^1H\}$ NMR (101 MHz, $CDCl_3$)

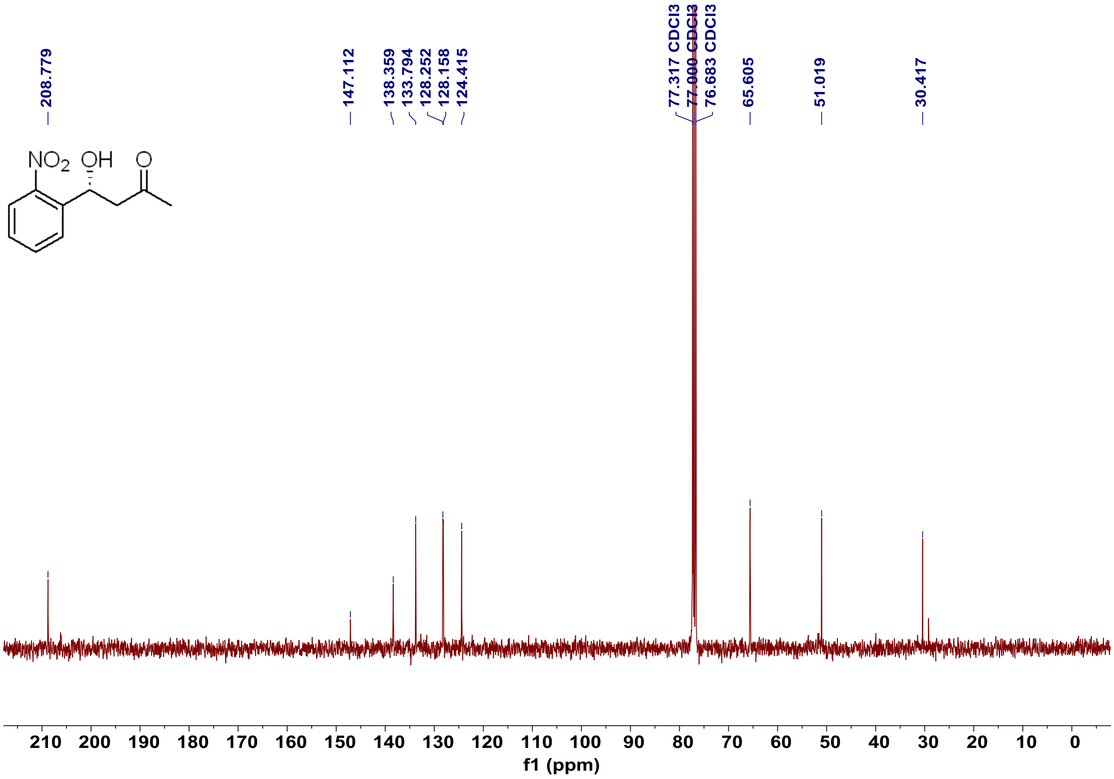

(*R*)-4-Hydroxy-4-(4-isocyanophenyl)butan-2-one (3d)

^{1}H NMR (400 MHz, $CDCl_3$)

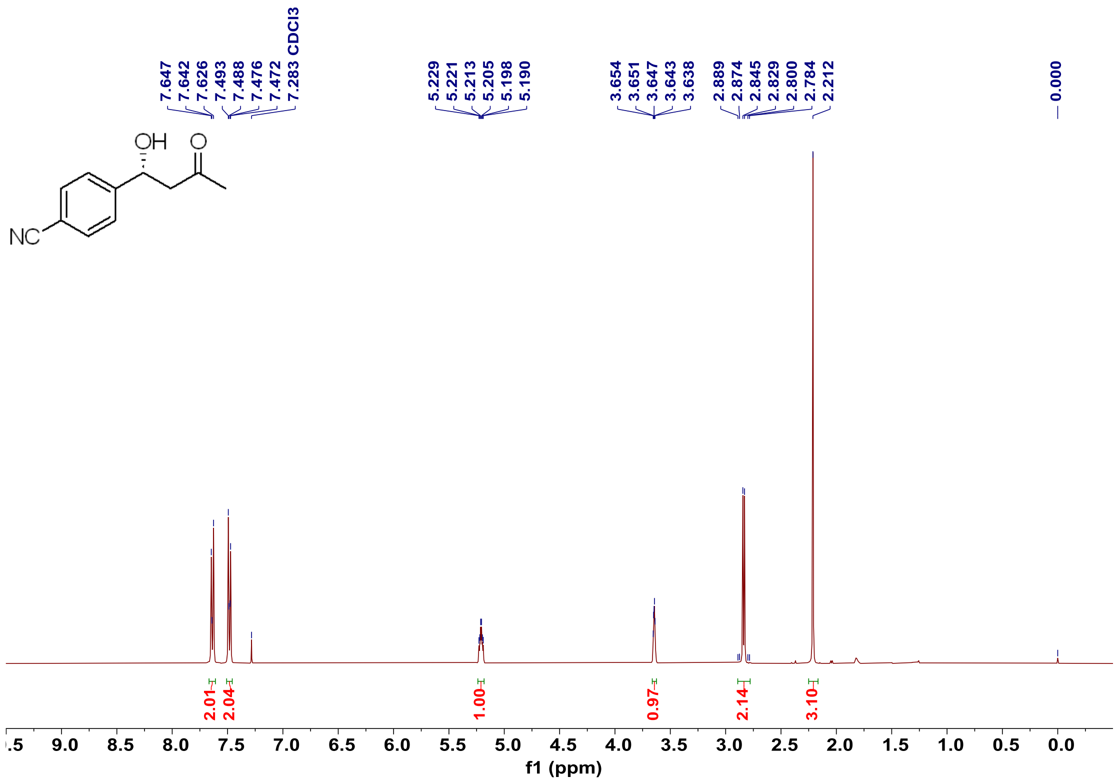

$^{13}C\{^{1}H\}$ NMR (101 MHz, $CDCl_3$)

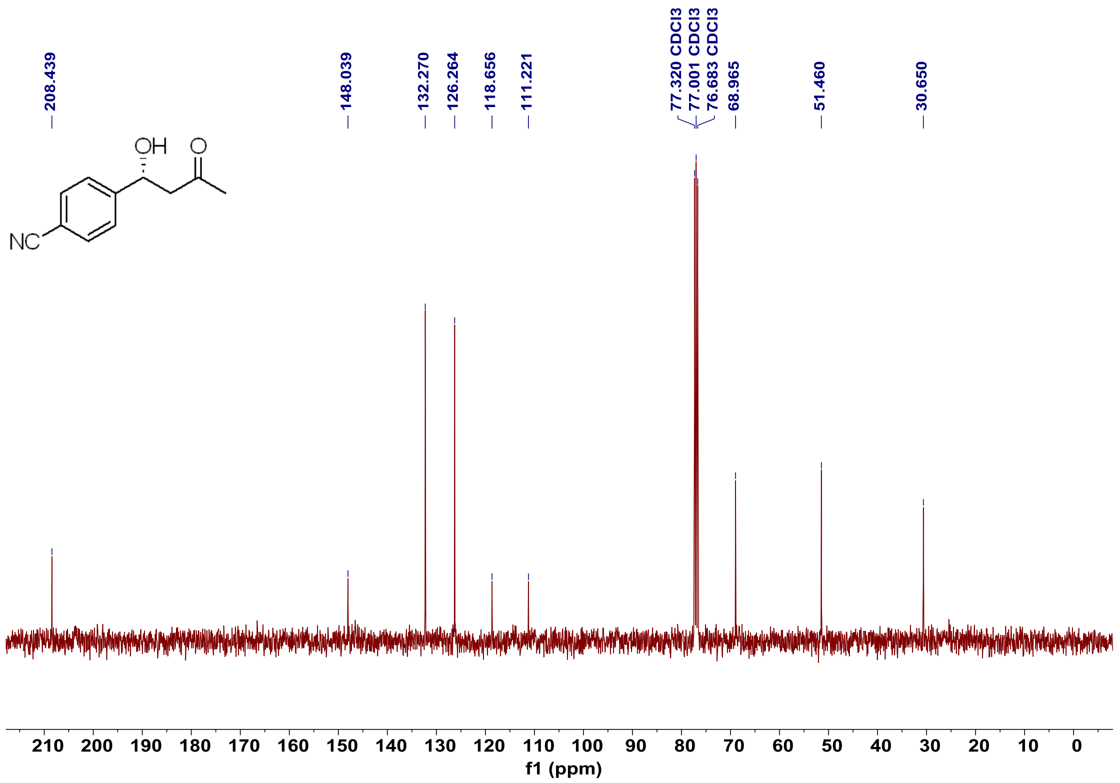

(*R*)-4-(4-Chlorophenyl)-4-hydroxybutan-2-one (3e)

^{1}H NMR (400 MHz, $CDCl_3$)

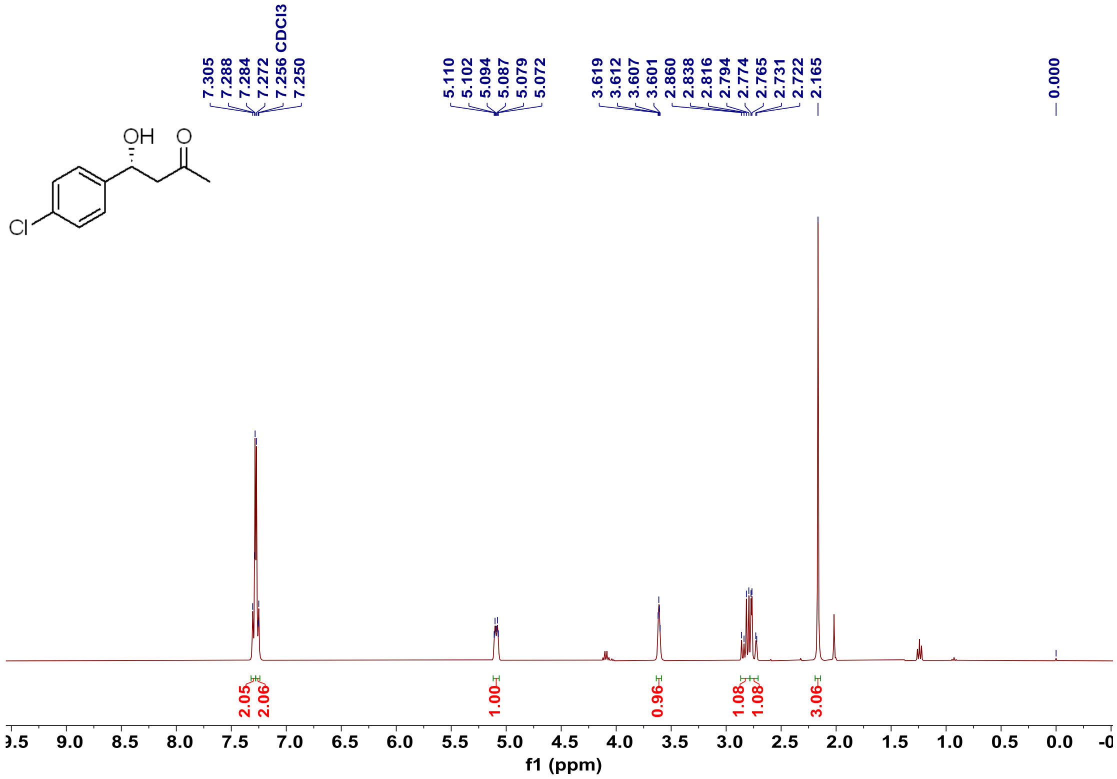

^{13}C{^{1}H} NMR (101 MHz, $CDCl_3$)

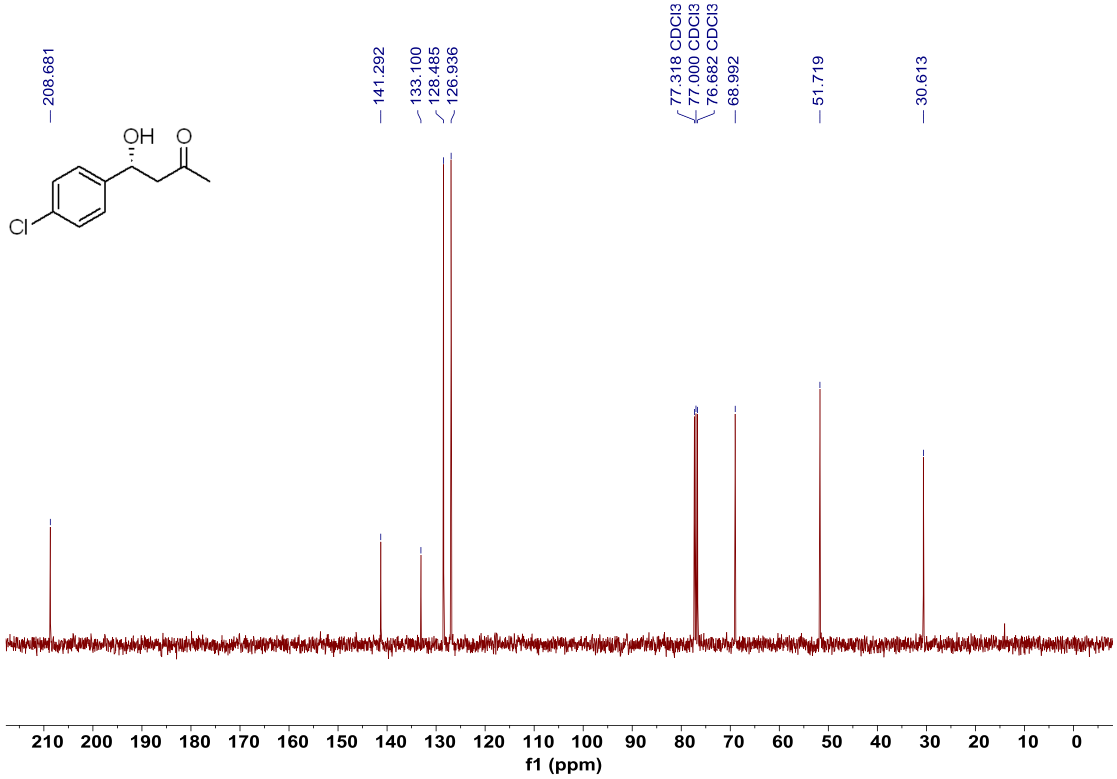

(*R*)-4-(4-Chlorophenyl)-4-hydroxybutan-2-one (3f)

^{1}H NMR (400 MHz, $CDCl_3$)

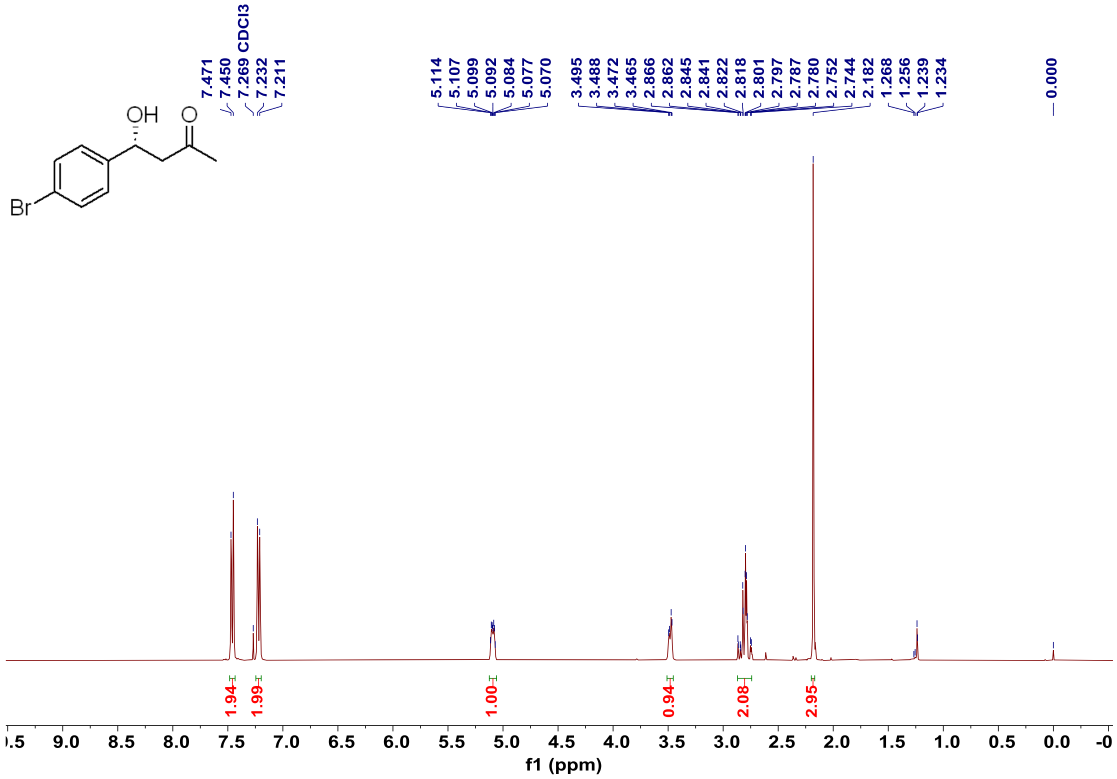

$^{13}C\{^{1}H\}$ NMR (101 MHz, $CDCl_3$)

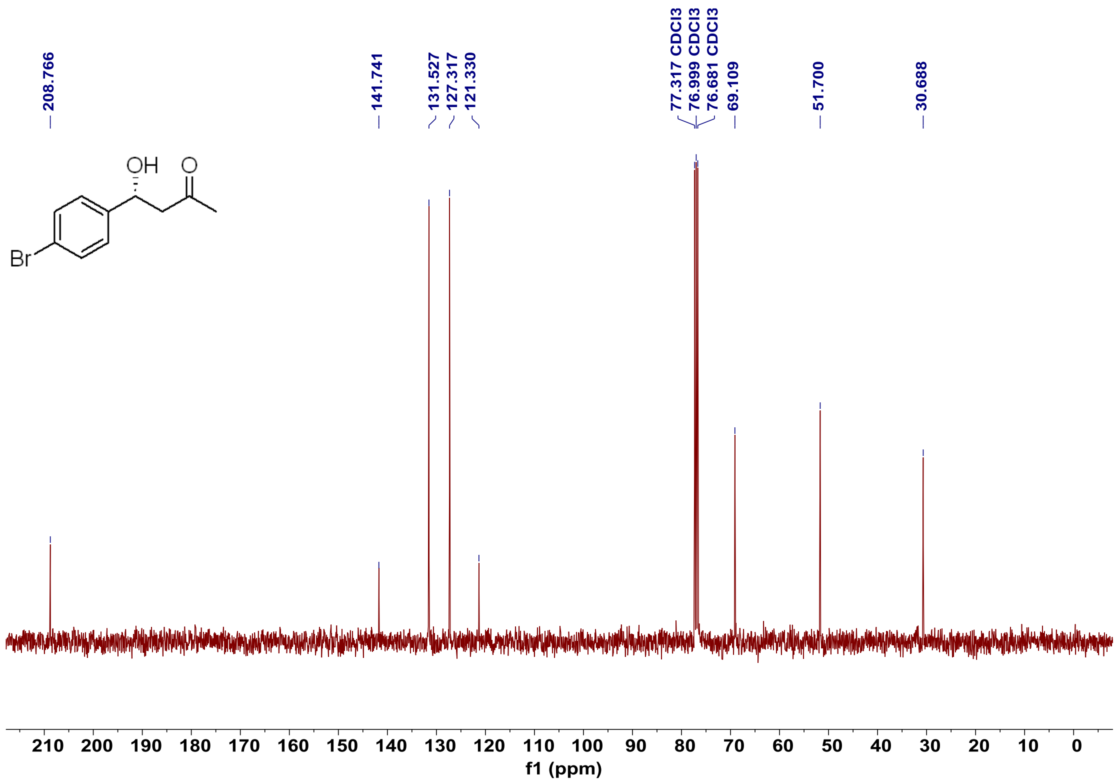

(*R*)-4-Hydroxy-4-(naphthalen-1-yl)butan-2-one (3j)

^{1}H NMR (400 MHz, $CDCl_3$)

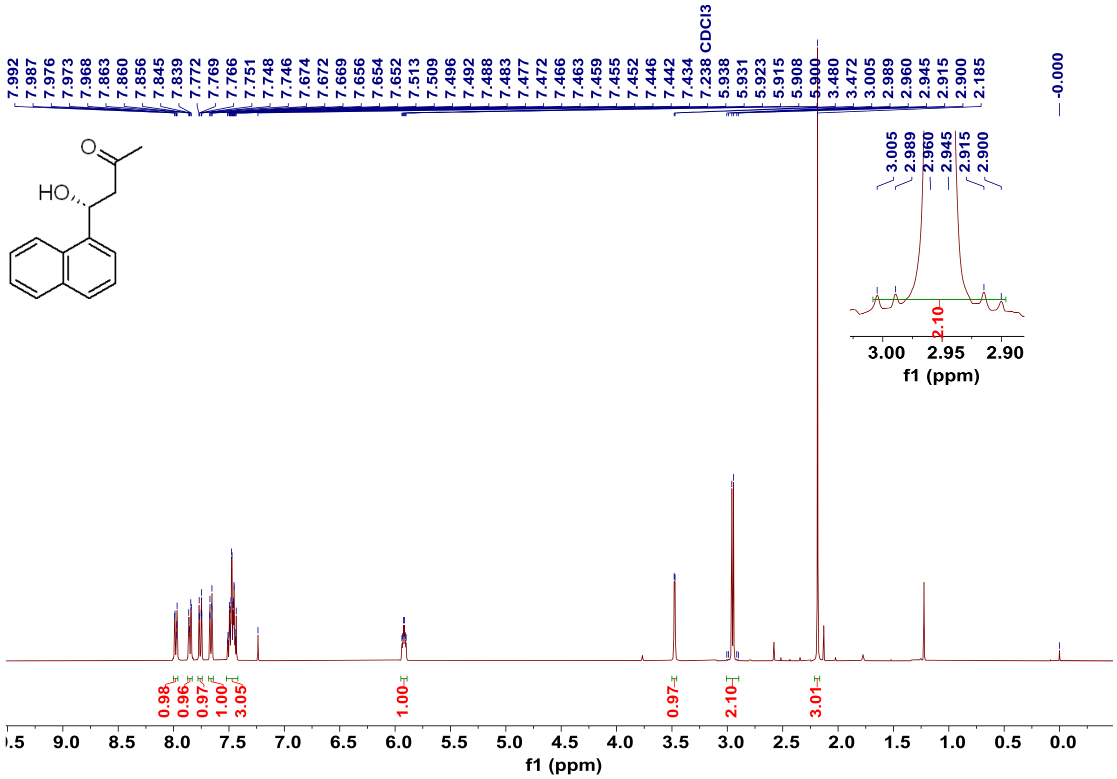

^{13}C{^{1}H} NMR (101 MHz, $CDCl_3$)

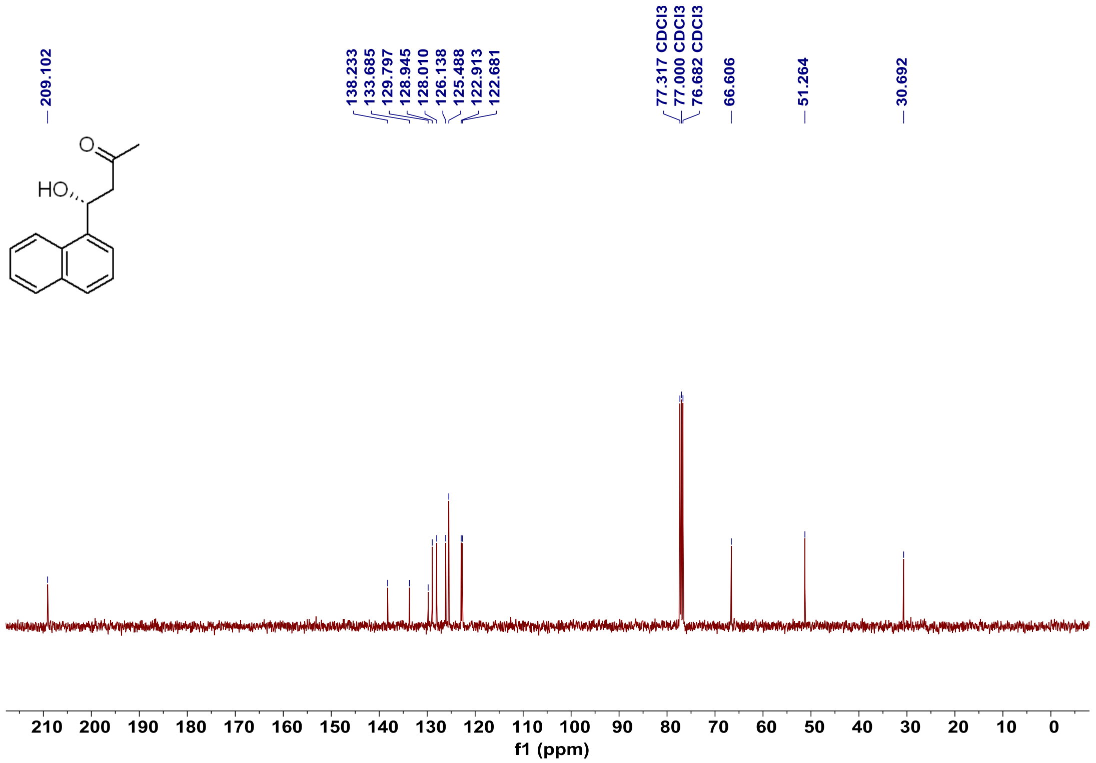

(R)-4-hydroxy-4-(naphthalen-2-yl)butan-2-one (3k)

^{1}H NMR (400 MHz, $CDCl_3$)

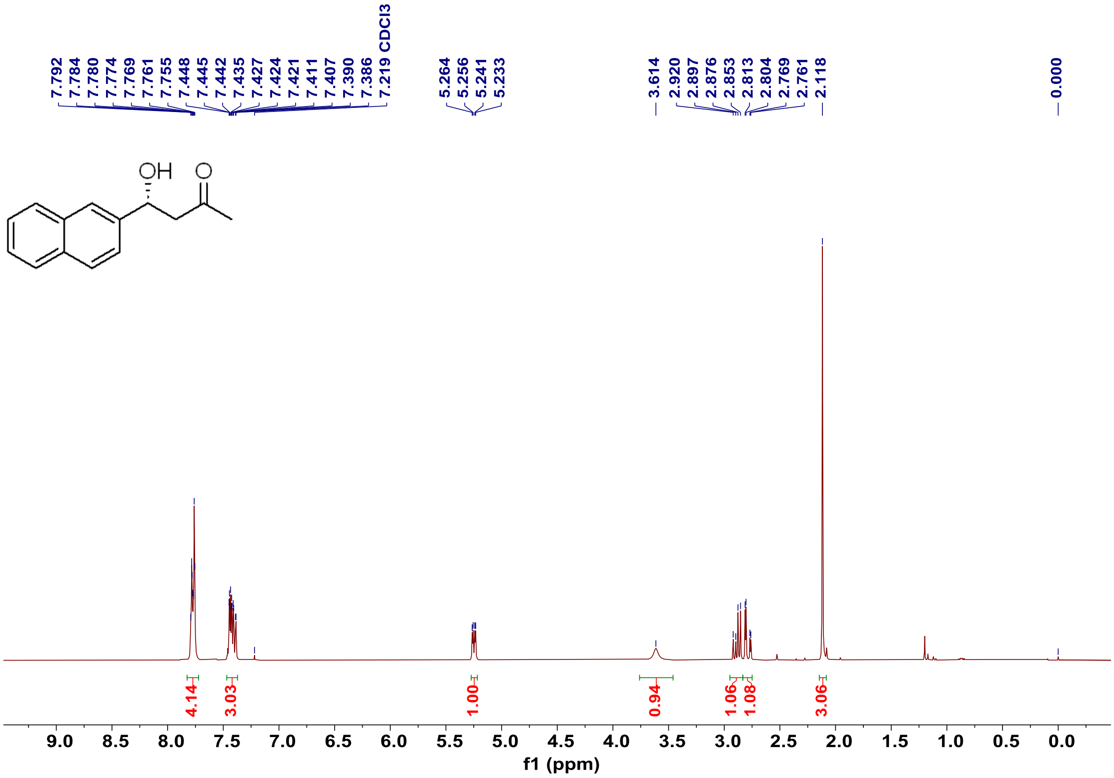

$^{13}C\{^{1}H\}$ NMR (101 MHz, $CDCl_3$)

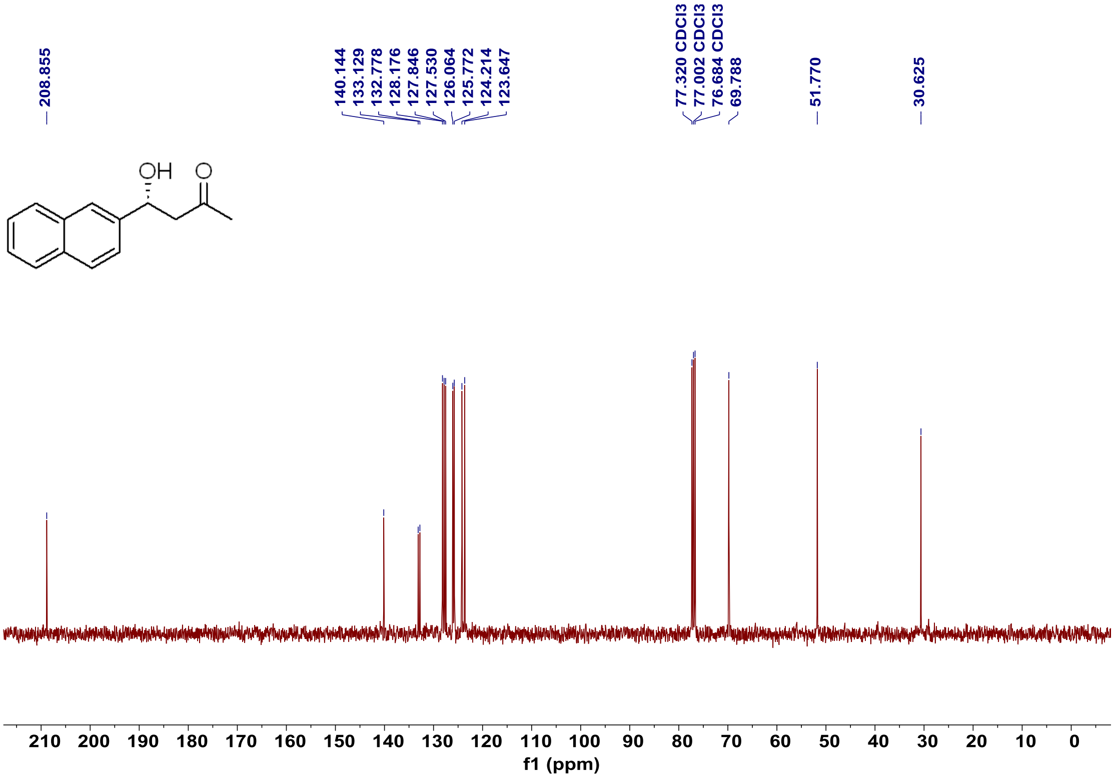

(*R*)-4-Hydroxy-4-(quinolin-4-yl)butan-2-one (3l)

^{1}H NMR (400 MHz, $CDCl_3$)

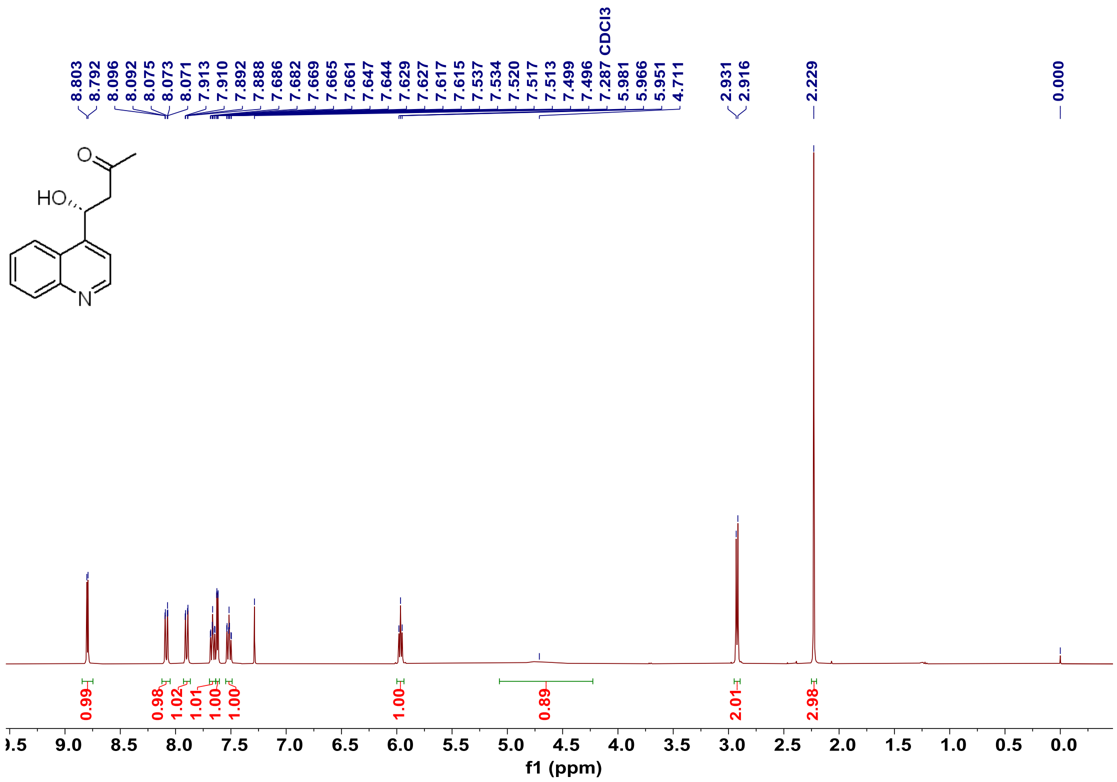

$^{13}C\{^{1}H\}$ NMR (101 MHz, $CDCl_3$)

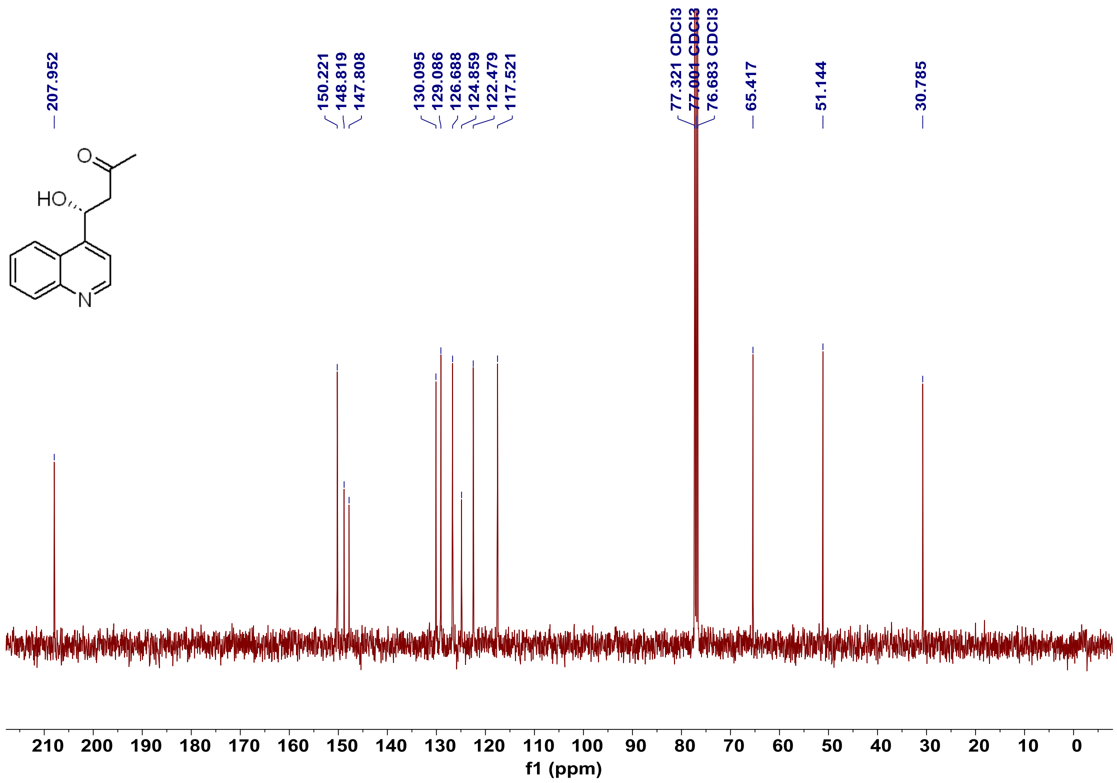

(*R*)-4-Hydroxy-4-(4-(methylsulfonyl)phenyl)butan-2-one (3m)

^{1}H NMR (400 MHz, $CDCl_3$)

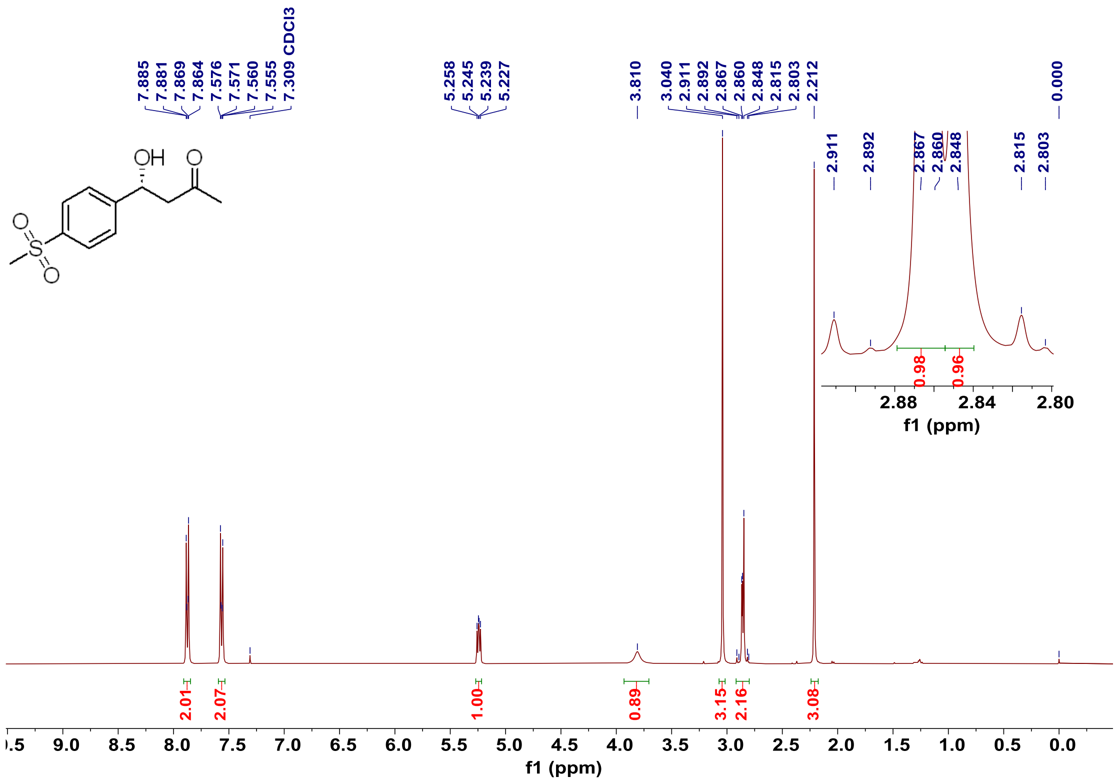

^{13}C{^{1}H} NMR (101 MHz, $CDCl_3$)

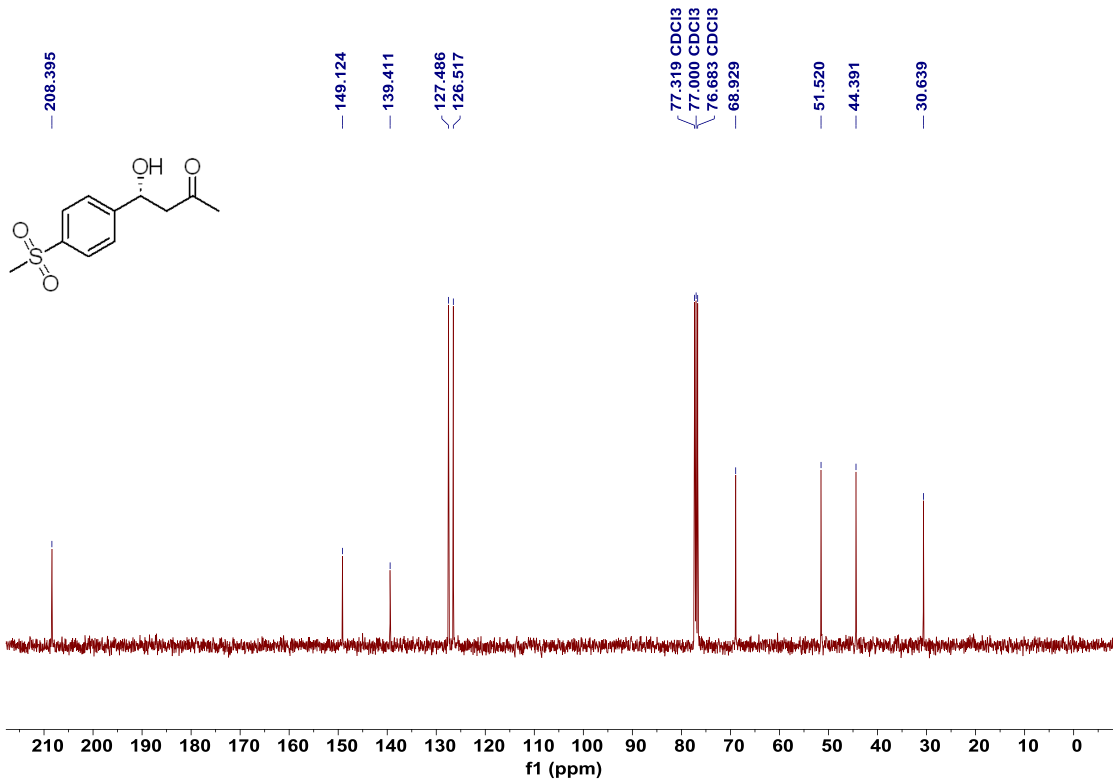

HRMS (ESI): *m*/*z* calcd for $C_{11}H_{14}NaO_4S^+$ $[M+Na]^+$: 265.0505, found: 265.0513.

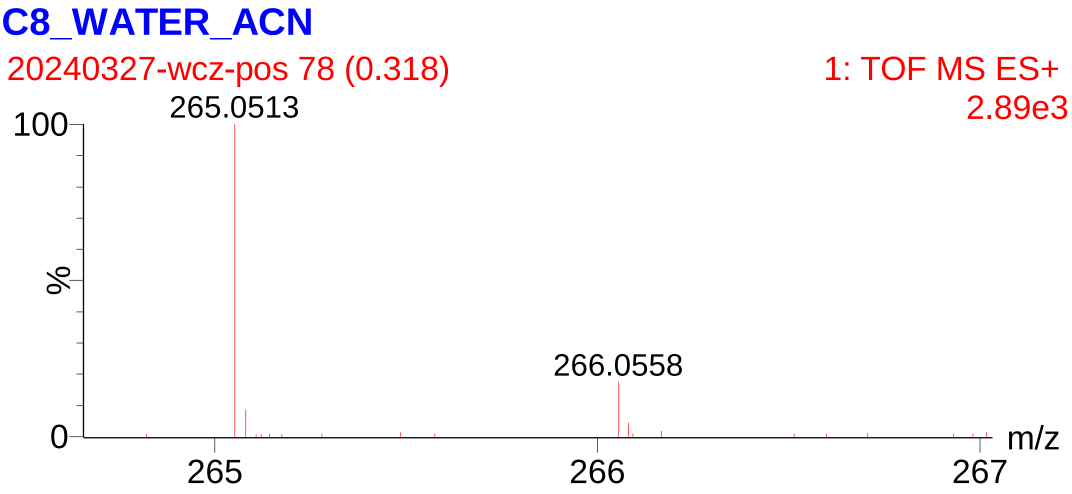

(*E*)-4-(4-(Methylsulfonyl)phenyl)but-3-en-2-one (4m)

^{1}H NMR (400 MHz, $CDCl_3$)

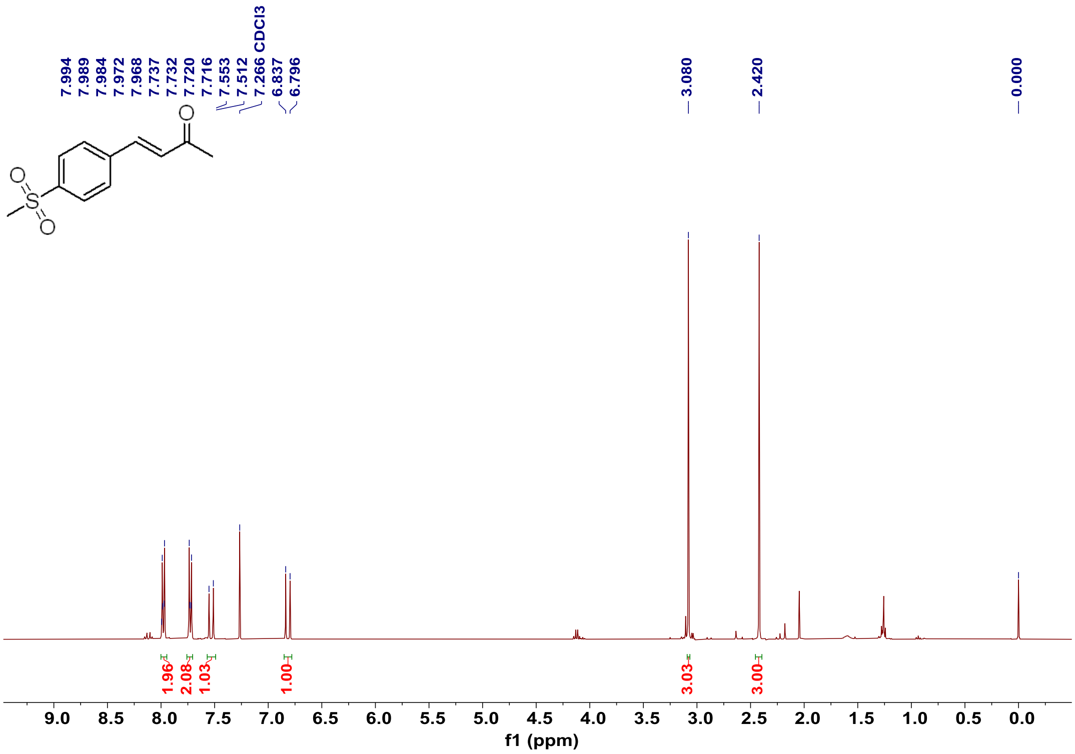

^{13}C{^{1}H} NMR (101 MHz, $CDCl_3$)

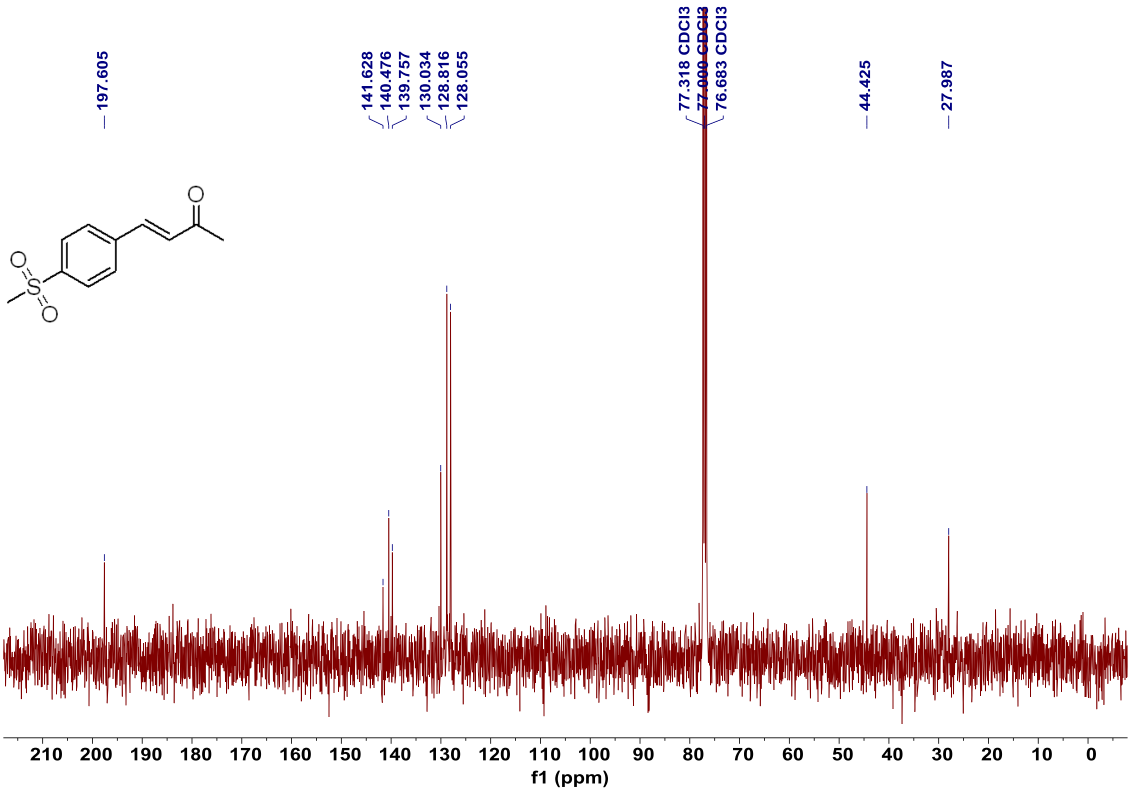

(*R*)-4-Hydroxy-4-(4-(4,4,5,5-tetramethyl-1,3,2-dioxaborolan-2-yl)phenyl)butan-2-one (3n)

^{1}H NMR (400 MHz, $CDCl_3$)

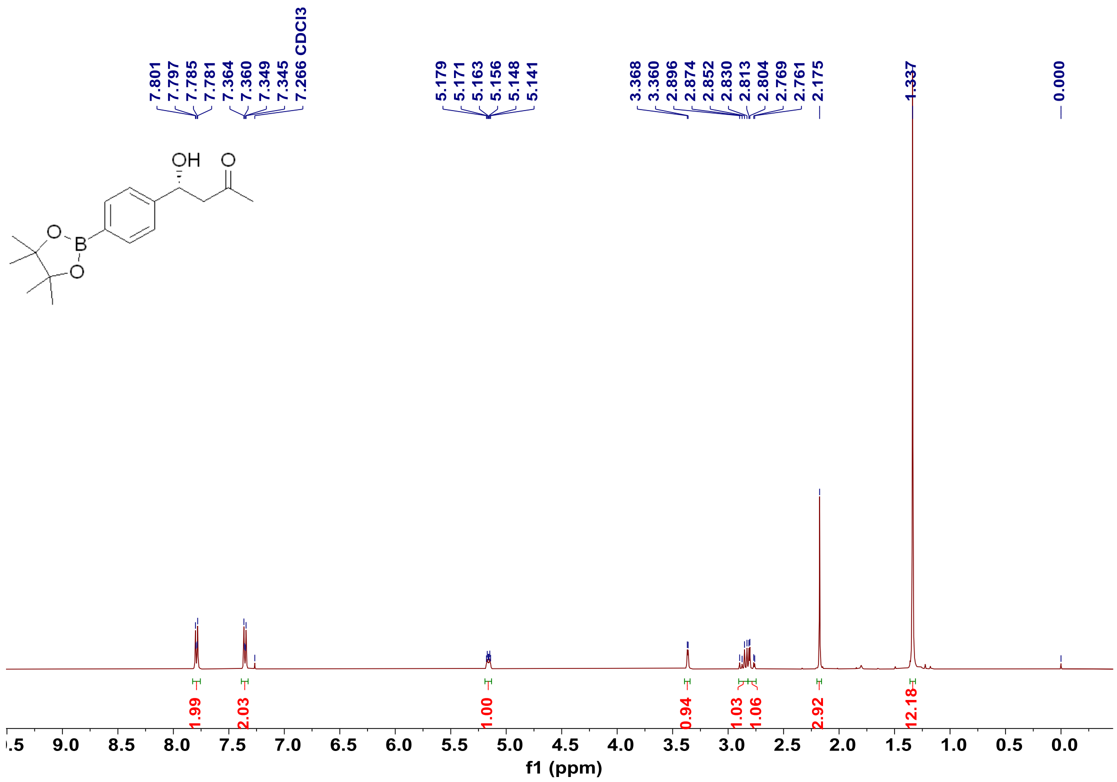

$^{13}C\{^{1}H\}$ NMR (101 MHz, $CDCl_3$)

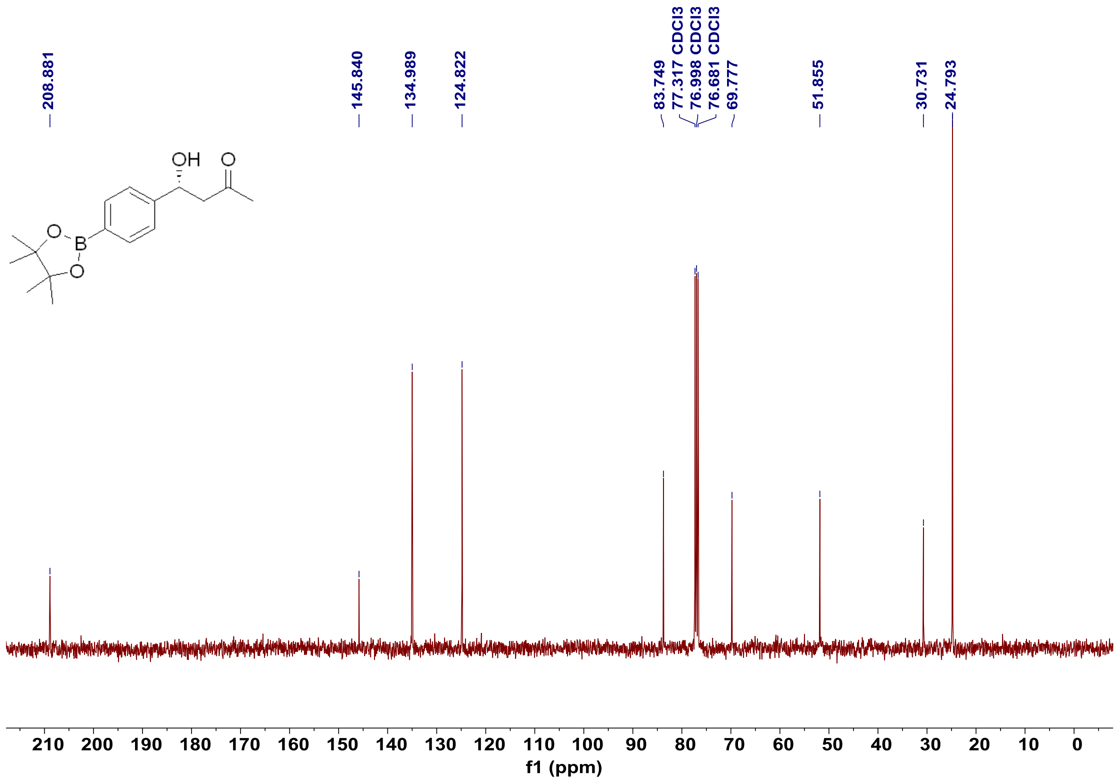

HRMS (ESI): *m/z* calcd for $C_{16}H_{23}BNaO_4^+$ [M+Na]$^+$: 313.1582, found: 313.1585.

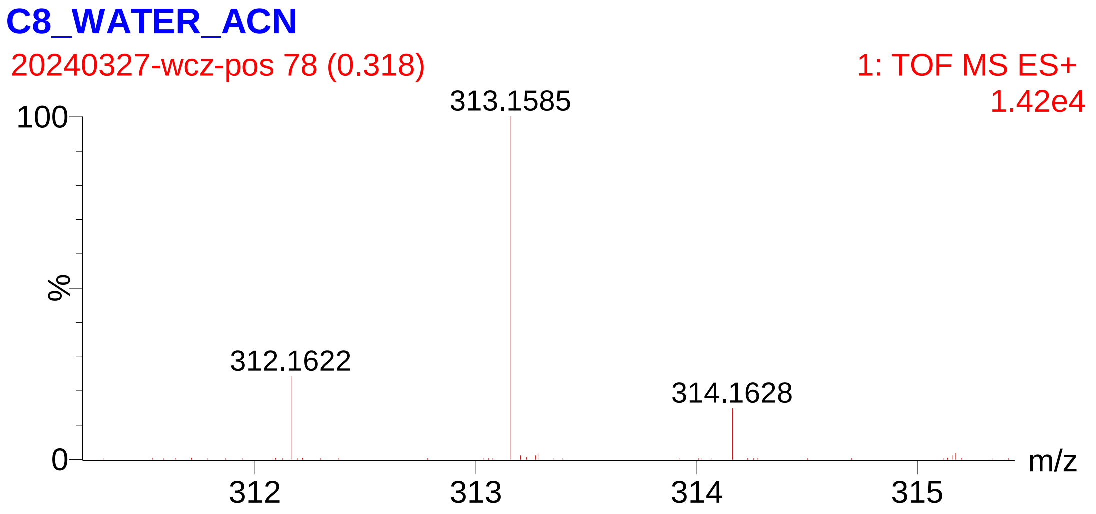

(*S*)-2-((*R*/*S*)-Hydroxy(4-nitrophenyl)methyl)cyclobutan-1-one (5a)

anti:*syn* = 57:43, ee of *anti* 83%.

^{1}H NMR (400 MHz, $CDCl_3$)

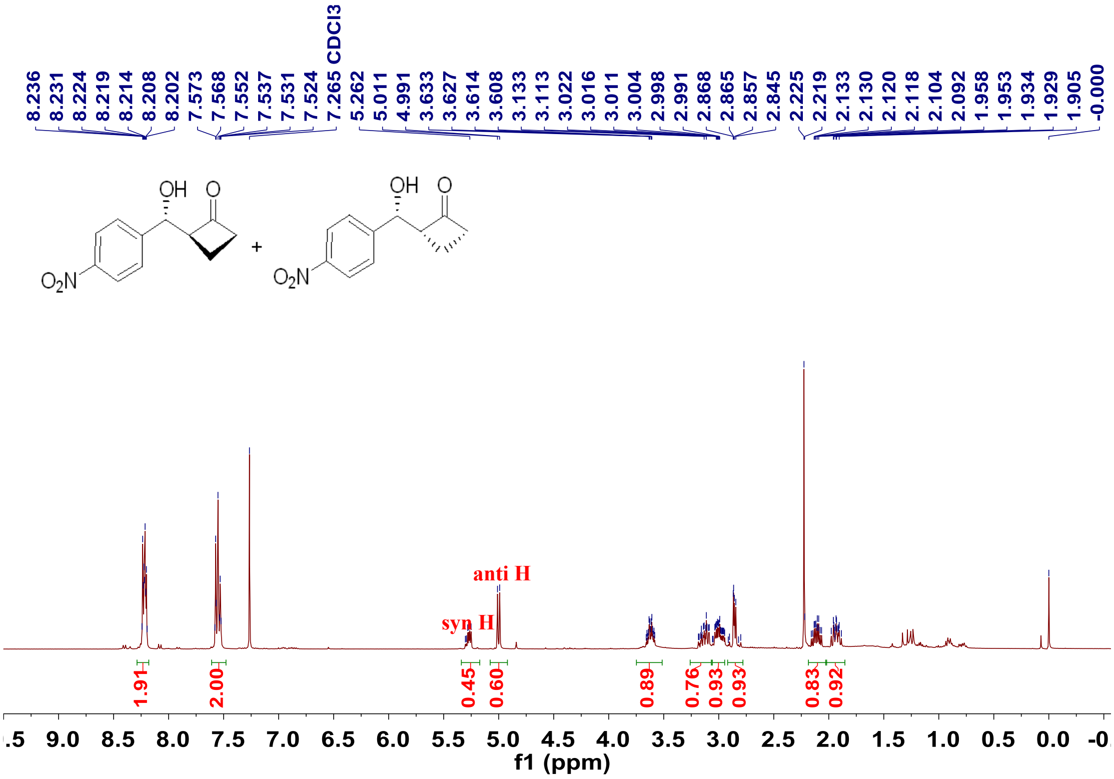

^{13}C{^{1}H} NMR (101 MHz, $CDCl_3$)

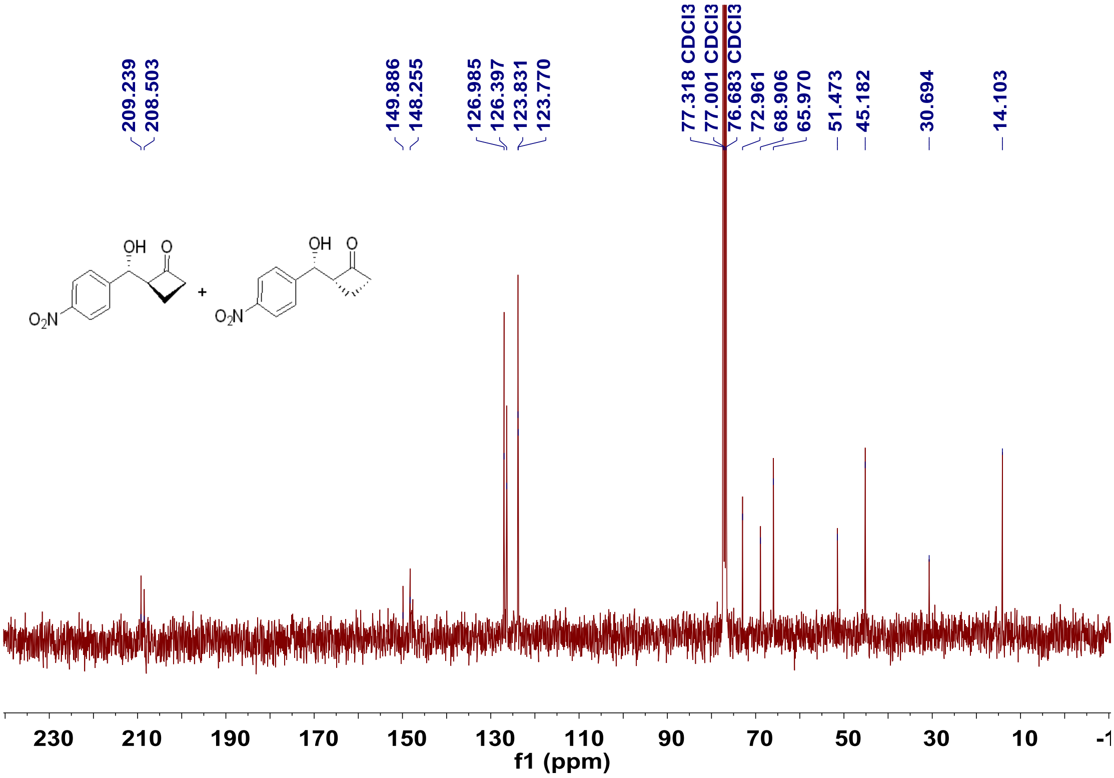

(*S*)-2-((*R*)-Hydroxy(4-nitrophenyl)methyl)cyclopentan-1-one (5b)

anti:*syn* = 83:17, ee of *anti* 96%

^{1}H NMR (400 MHz, $CDCl_3$)

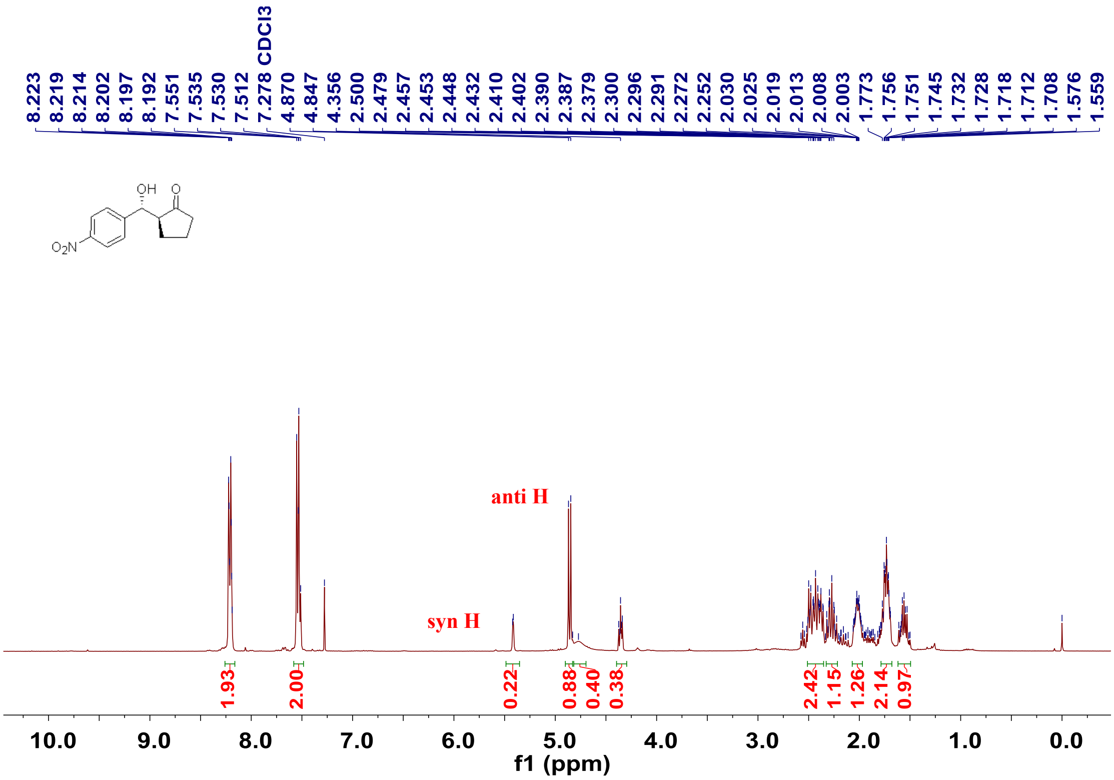

^{13}C{^{1}H} NMR (101 MHz, $CDCl_3$)

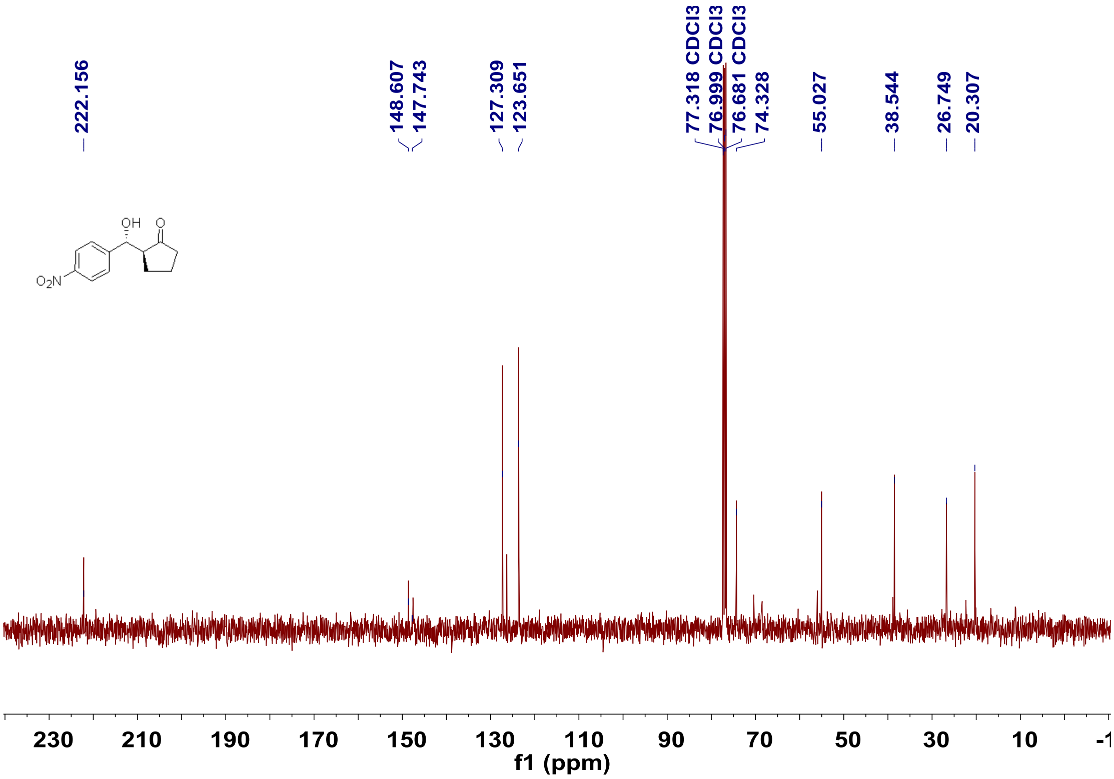

(*S*)-2-((*R*)-Hydroxy(4-nitrophenyl)methyl)cyclohexan-1-one (5c)

anti:*syn* = 94:6, ee of *anti* 90%

^{1}H NMR (400 MHz, $CDCl_3$)

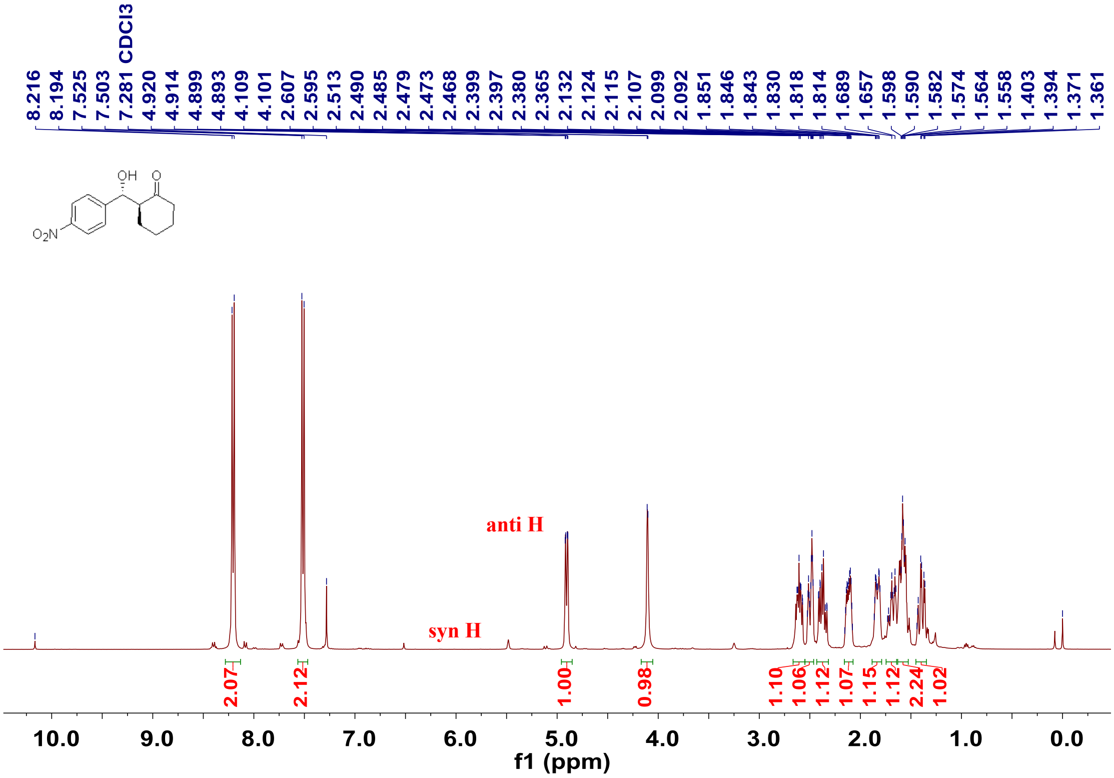

^{13}C{^{1}H} NMR (101 MHz, $CDCl_3$)

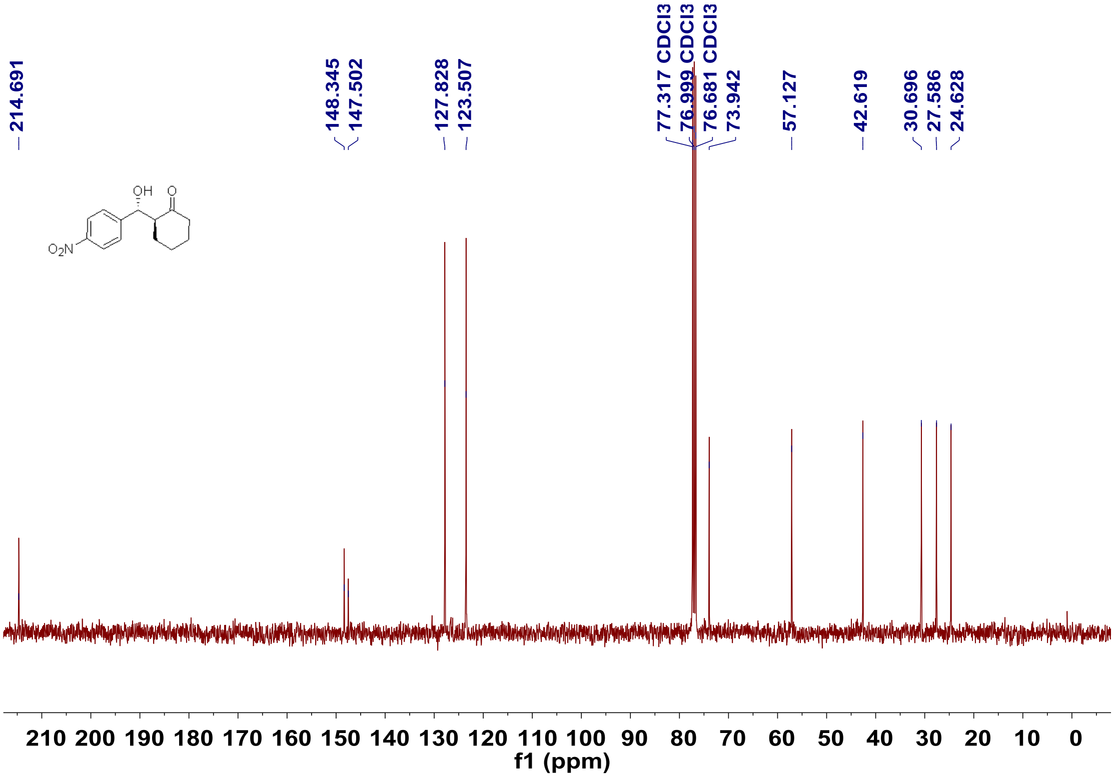

(1*R*,2*S*)-Hydroxy-2-methyl-1-(4-nitrophenyl)pentan-3-one (5d)

anti:*syn* = 86:14, ee of *anti* 98%.

^{1}H NMR (400 MHz, $CDCl_3$)

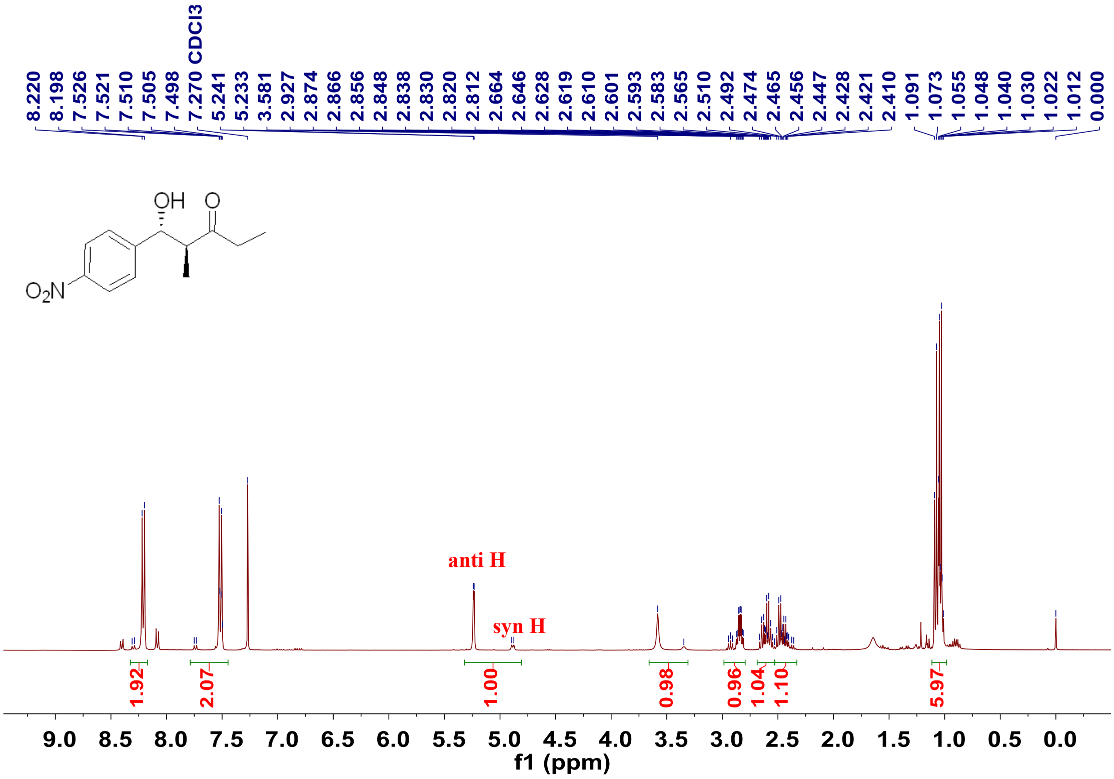

^{13}C{^{1}H} NMR (101 MHz, $CDCl_3$)

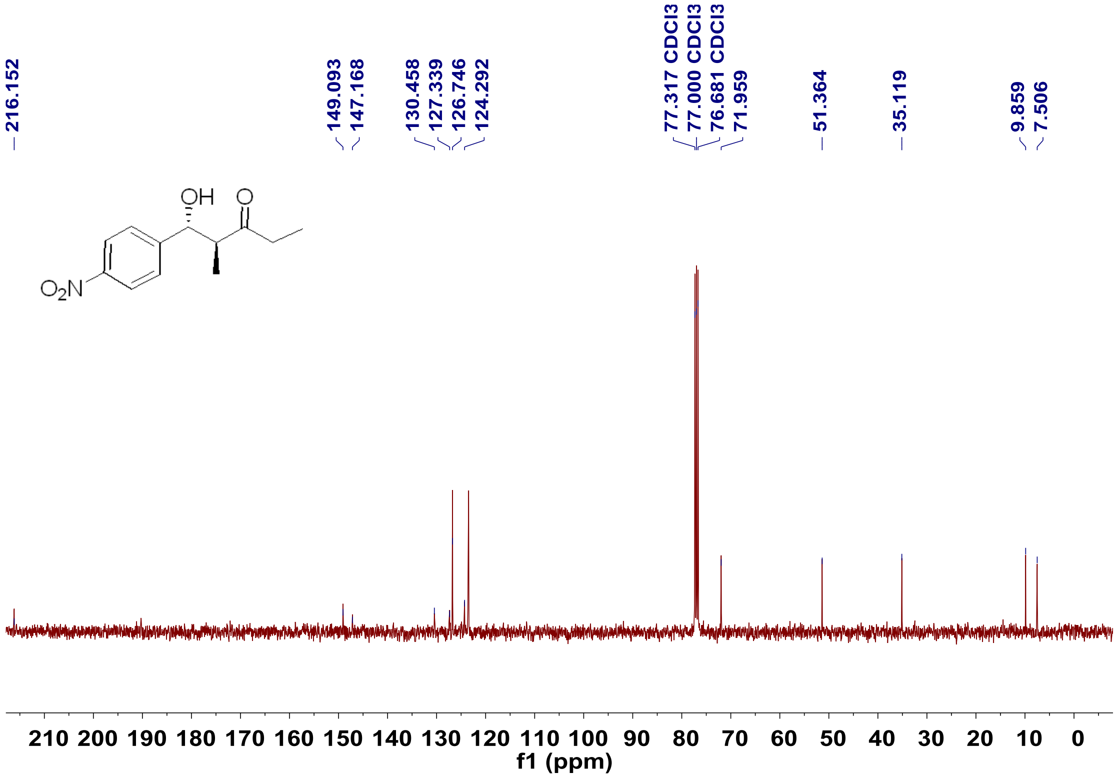

(*R*)-3-Hydroxy-3-(4-nitrophenyl)-1-phenylpropan-1-one (5e)

^{1}H NMR (400 MHz, $CDCl_3$)

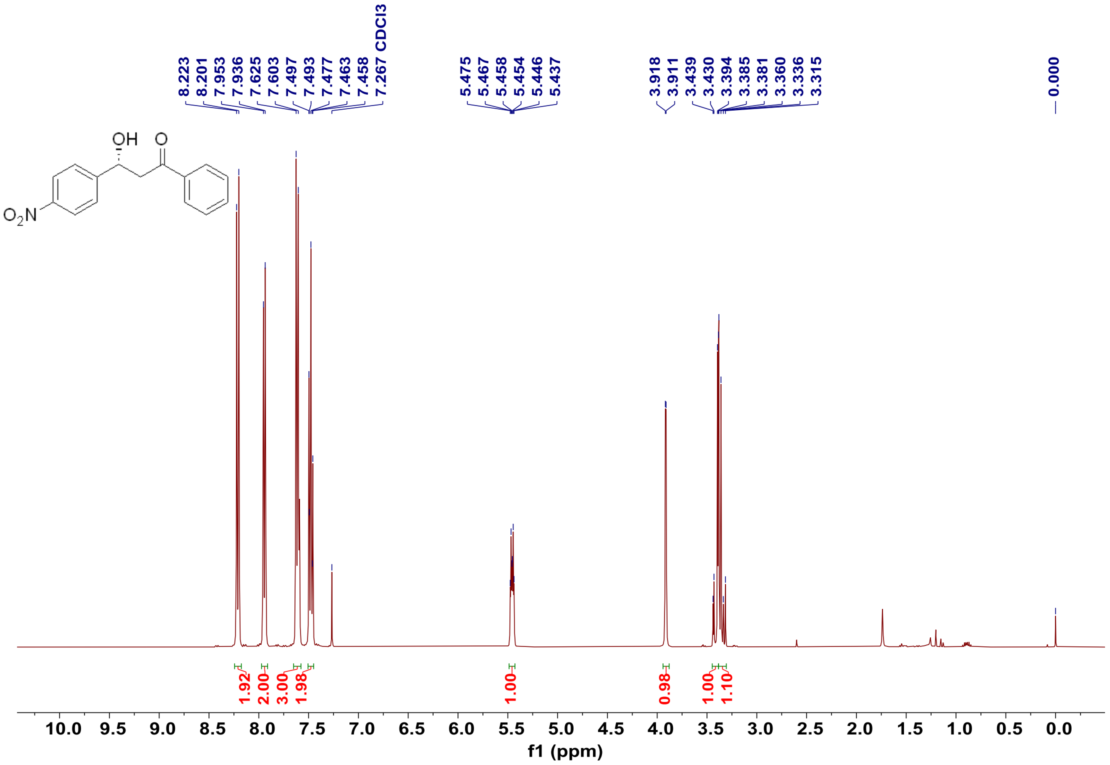

^{13}C{^{1}H} NMR (101 MHz, $CDCl_3$)

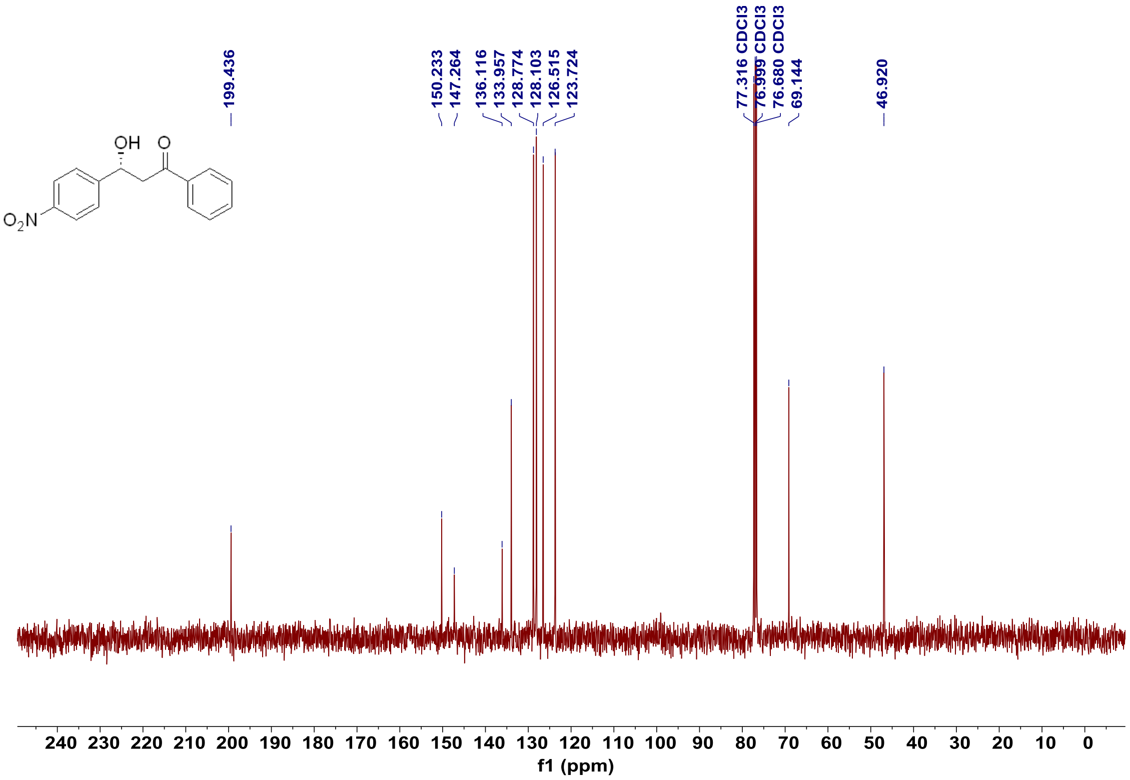

Copies of HPLC profiles for the determination of enantiomeric excesses of aldol adducts

HPLC profiles for aldol adduct **3a**

AS-H, *i*-PrOH/Hex 25/75, flow rate = 1.0 mL/min

Racemic product

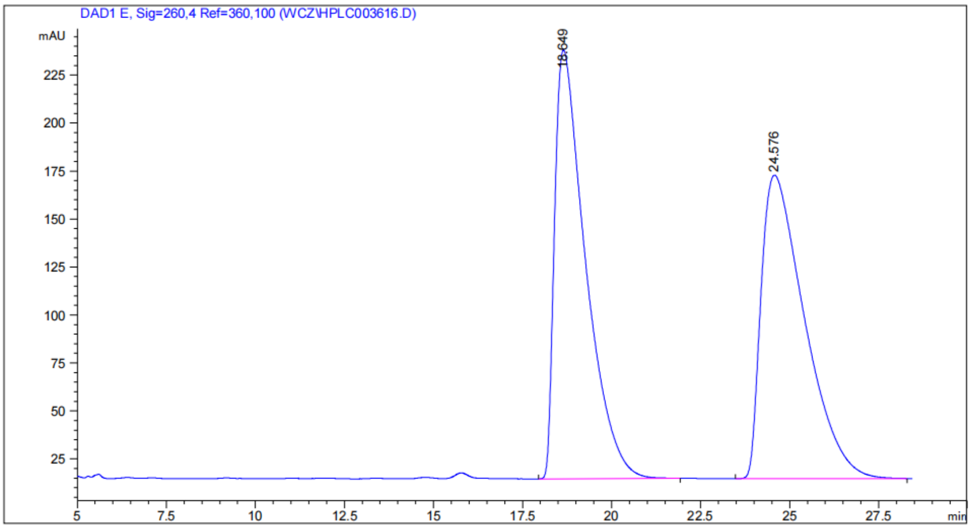

Peak	Ret.Time [min]	Type	Width [min]	Area[Mau*s]	Height [mAU]	Area%
1	18.649	BB	0.8901	1.34473e4	223.45892	50.1105
2	24.576	BB	1.2818	1.33880e4	158.11673	49.8895

Asymmetric catalytic product

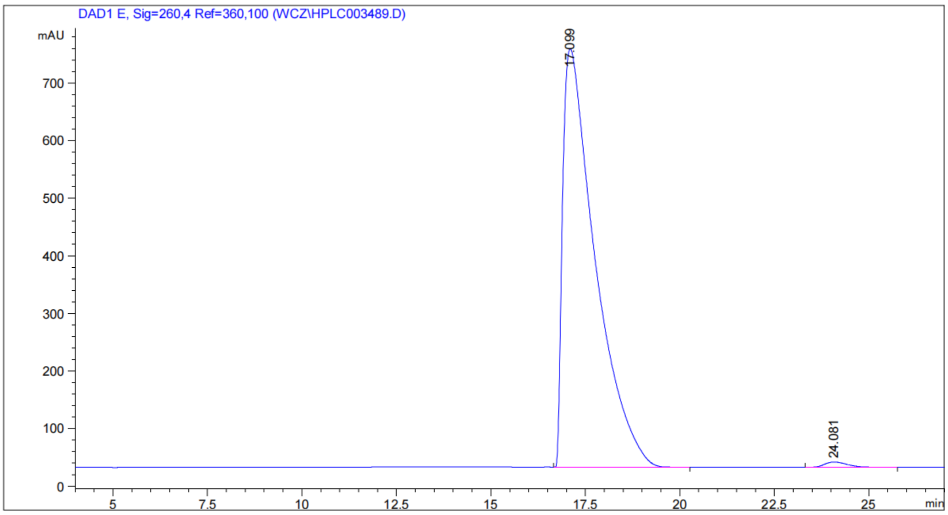

Peak	Ret.Time [min]	Type	Width [min]	Area[Mau*s]	Height [mAU]	Area%
1	17.099	BB	0.8669	4.32646e4	726.36804	99.1429
2	24.081	BB	0.6548	374.04776	9.03851	0.8571

HPLC profiles for aldol adduct **3b**

AD-H, *i*-PrOH/Hex 10/90, flow rate = 1.0 mL/min

Racemic product

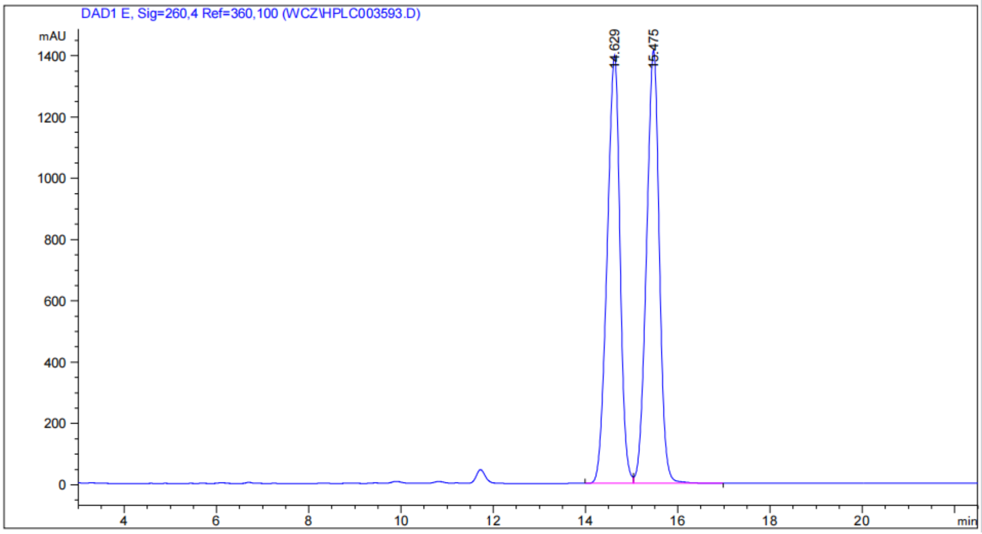

Peak	Ret.Time [min]	Type	Width [min]	Area[Mau*s]	Height [mAU]	Area%
1	14.629	BV	0.2999	2.70145e4	1395.19458	49.7618
2	15.475	VB	0.2973	2.72731e4	1412.34058	50.2382

Asymmetric catalytic product

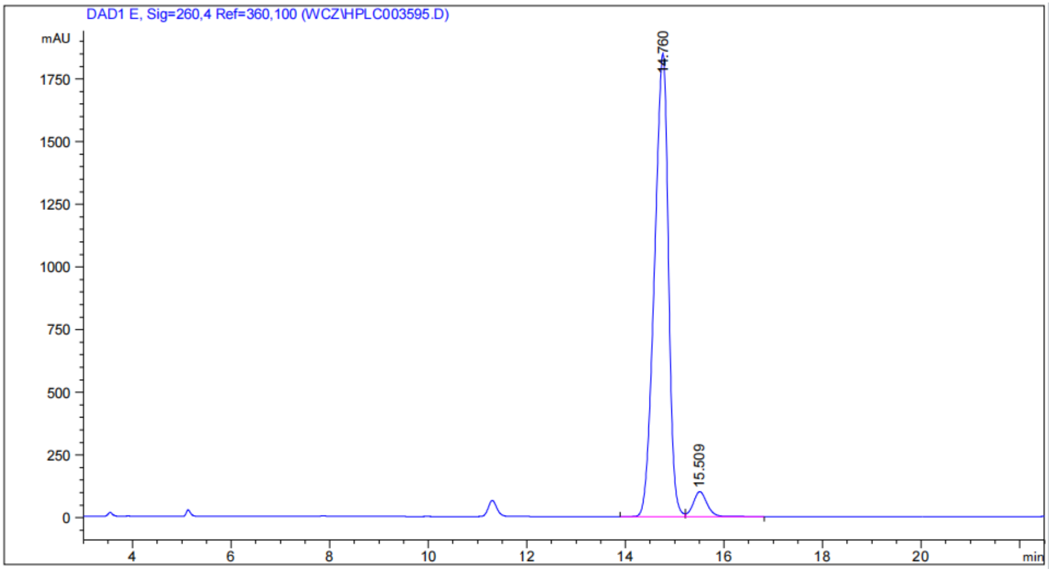

Peak	Ret.Time [min]	Type	Width [min]	Area[Mau*s]	Height [mAU]	Area%
1	14.760	BV	0.3025	3.61705e4	1846.51892	94.9498
2	15.509	VB	0.2985	1923.85486	99.10278	5.0502

HPLC profiles for aldol adduct **3c**

AD-H, *i*-PrOH/Hex 5/95, flow rate = 1.0 mL/min

Racemic product

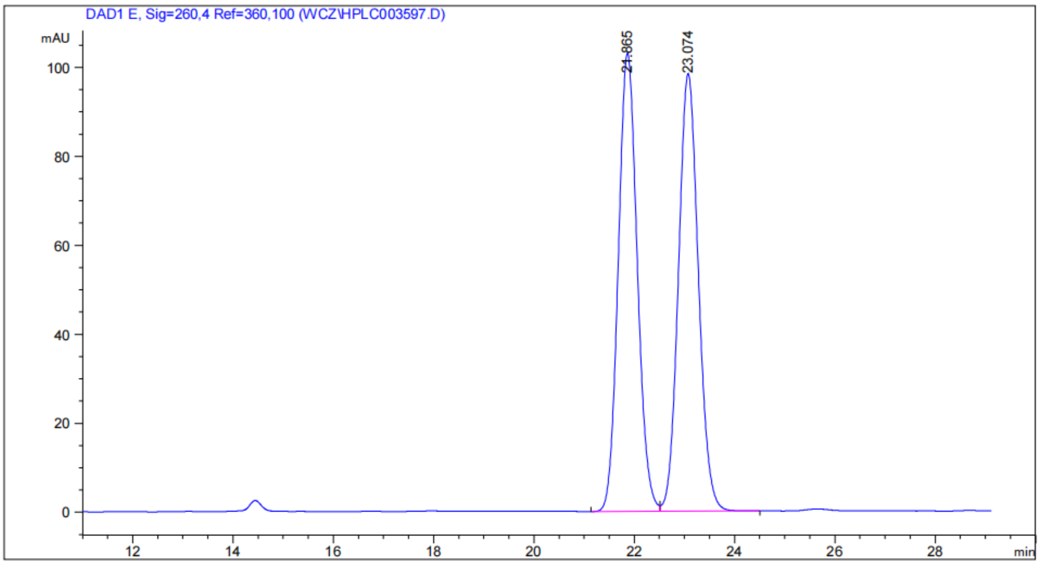

Peak	Ret.Time [min]	Type	Width [min]	Area[Mau*s]	Height [mAU]	Area%
1	21.865	BV	0.4066	2706.45996	102.98327	49.9141
2	23.074	VB	0.4282	2715.77759	98.34523	50.0859

Asymmetric catalytic product

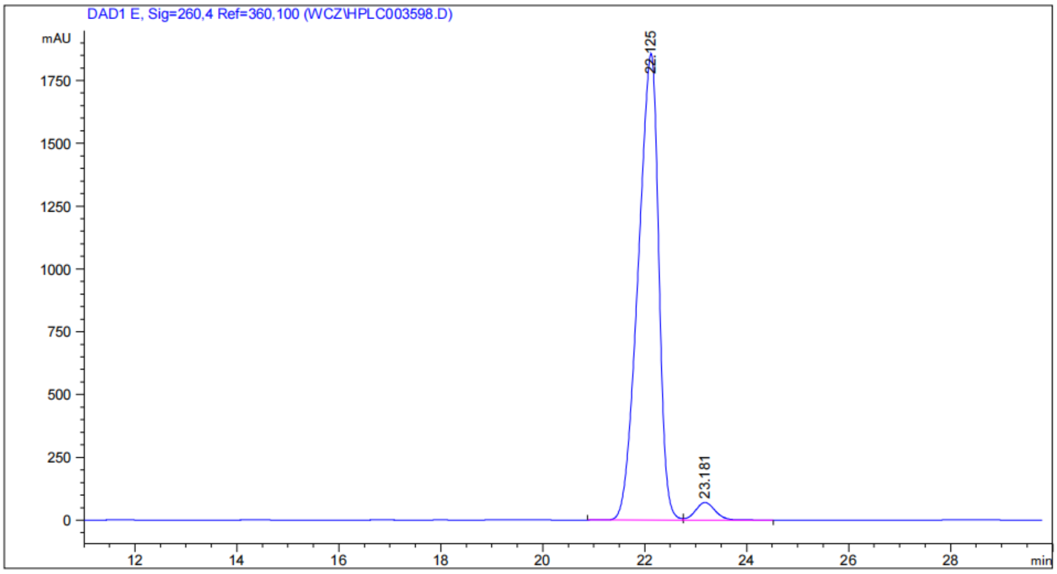

Peak	Ret.Time [min]	Type	Width [min]	Area[Mau*s]	Height [mAU]	Area%
1	22.125	BV	0.4332	5.17262e4	1855.85608	96.5365
2	23.181	VB	0.4114	1855.82910	69.96798	3.4635

HPLC profiles for aldol adduct **3d**

AS-H, *i*-PrOH/Hex 30/70, flow rate = 1.0 mL/min

Racemic product

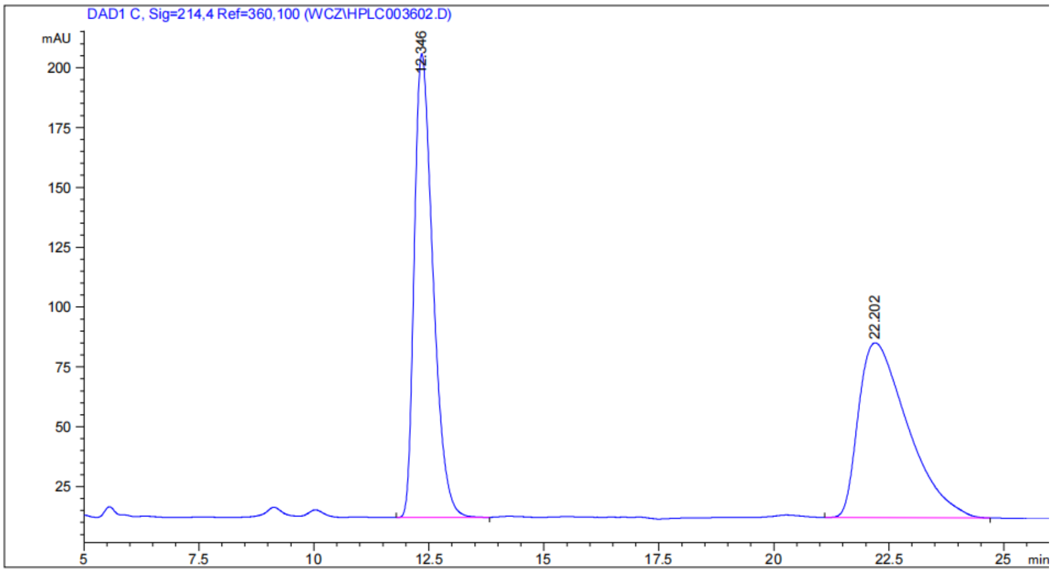

Peak	Ret.Time [min]	Type	Width [min]	Area[Mau*s]	Height [mAU]	Area%
1	12.346	BB	0.4358	5477.55176	193.75621	50.1635
2	22.202	BB	1.1516	5441.83789	73.00826	49.8365

Asymmetric catalytic product

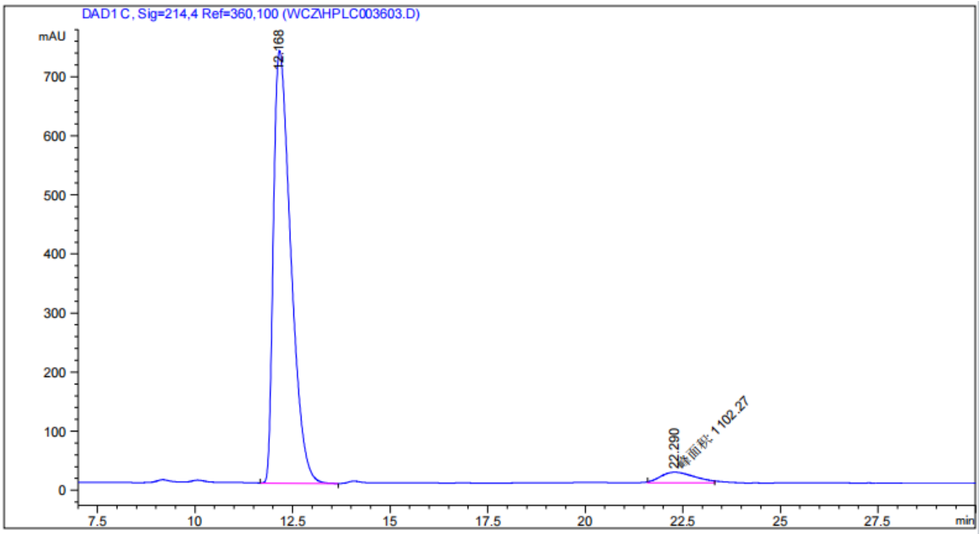

Peak	Ret.Time [min]	Type	Width [min]	Area[Mau*s]	Height [mAU]	Area%
1	12.168	BB	0.4806	2.26214e4	731.97998	95.3537
2	22.290	MM	1.0201	1102.26563	18.00859	4.6463

HPLC profiles for aldol adduct **3e**

AS-H, *i*-PrOH/Hex 15/85, flow rate = 1.0 mL/min

Racemic product

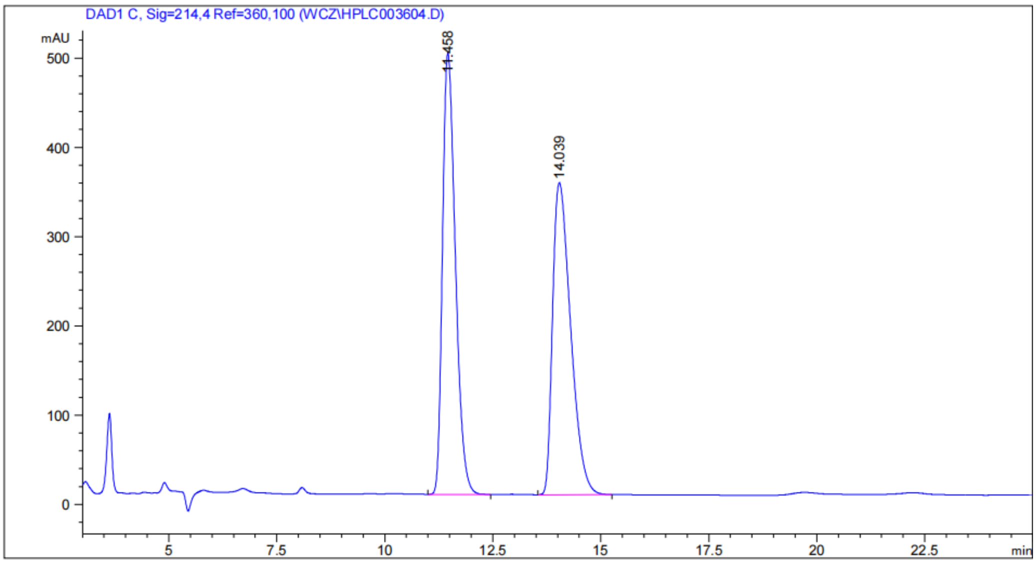

Peak	Ret.Time [min]	Type	Width [min]	Area[Mau*s]	Height [mAU]	Area%
1	11.458	BB	0.3223	1.02650e4	494.34796	50.2015
2	14.039	BB	0.4520	1.01826e4	349.38925	49.7985

Asymmetric catalytic product

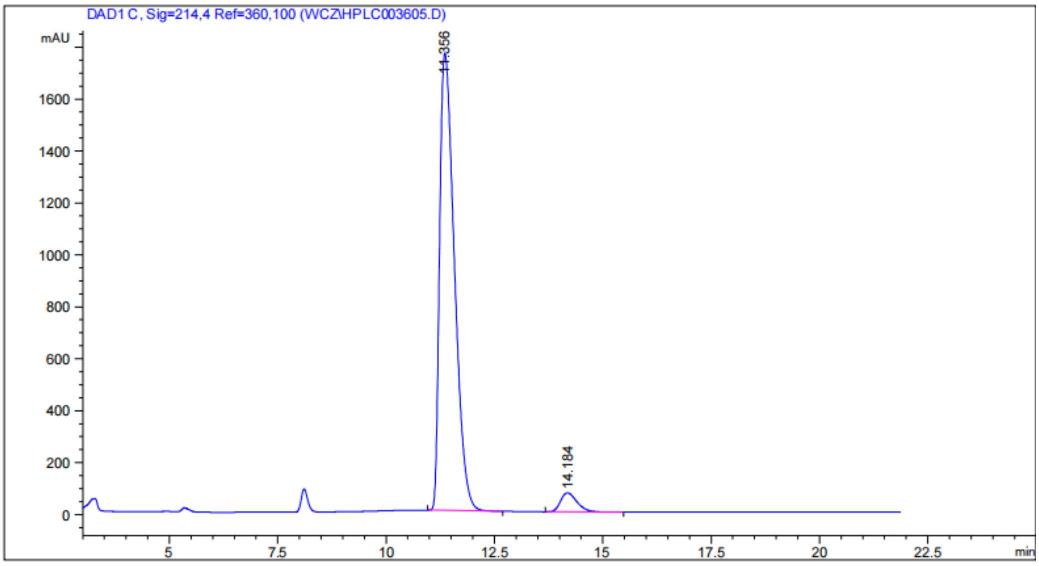

Peak	Ret.Time [min]	Type	Width [min]	Area[Mau*s]	Height [mAU]	Area%
1	11.356	BB	0.3698	4.16144e4	1760.16138	95.5188
2	14.184	BB	0.4105	1952.30200	73.84138	4.4812

HPLC profiles for aldol adduct **3f**

AS-H, *i*-PrOH/Hex 20/80, flow rate = 1.0 mL/min

Racemic product

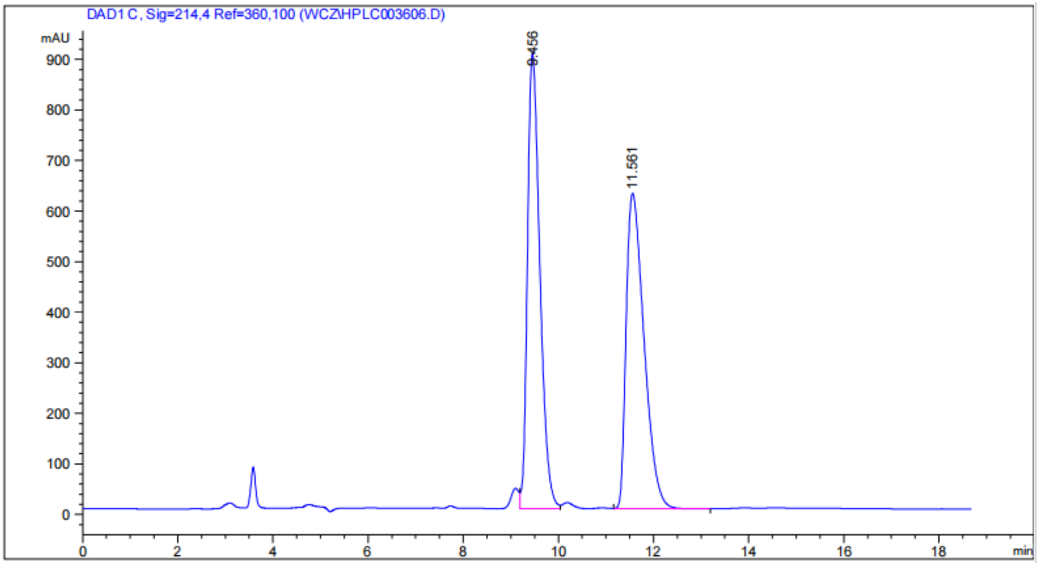

Peak	Ret.Time [min]	Type	Width [min]	Area[Mau*s]	Height [mAU]	Area%
1	9.456	VV	0.2790	1.62858e4	900.60876	50.1054
2	11.561	VB	0.4054	1.62173e4	623.52692	49.8946

Asymmetric catalytic product

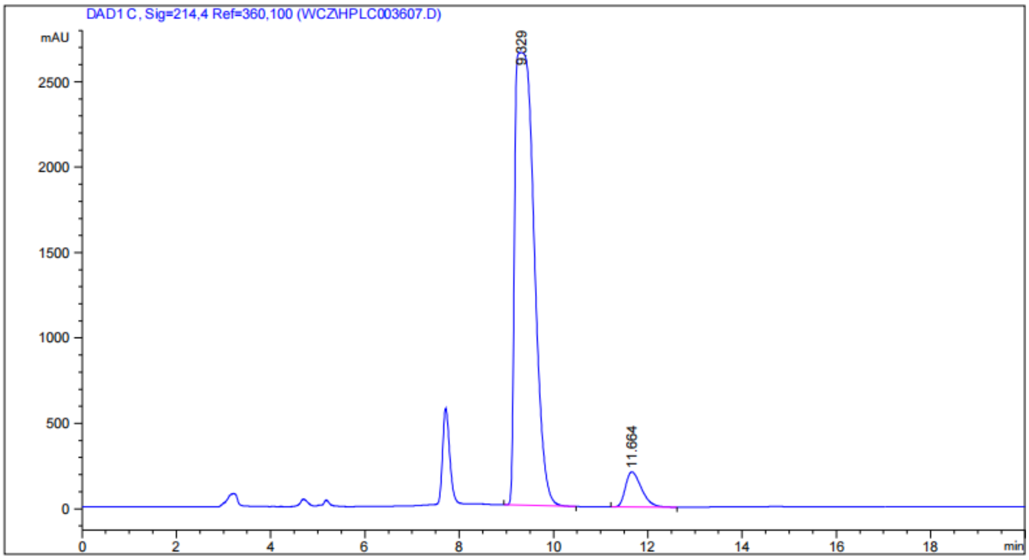

Peak	Ret.Time [min]	Type	Width [min]	Area[Mau*s]	Height [mAU]	Area%
1	9.329	BB	0.4507	7.50985e4	2649.60815	93.9306
2	11.664	BB	0.3654	4852.51123	205.55980	6.0694

HPLC profiles for aldol adduct **3j**

AS-H, *i*-PrOH/Hex 15/85, flow rate = 1.0 mL/min

Racemic product

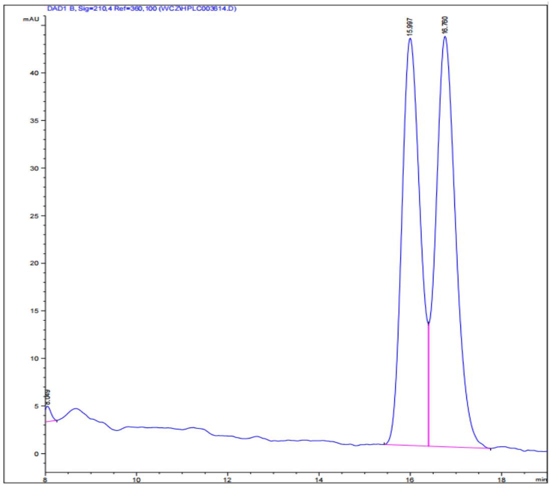

Peak	Ret.Time [min]	Type	Width [min]	Area[Mau*s]	Height [mAU]	Area%
1	15.997	BV	0.4322	1189.07642	42.78967	47.5846
2	16.760	VB	0.4585	1309.79163	43.10918	52.4154

Asymmetric catalytic product

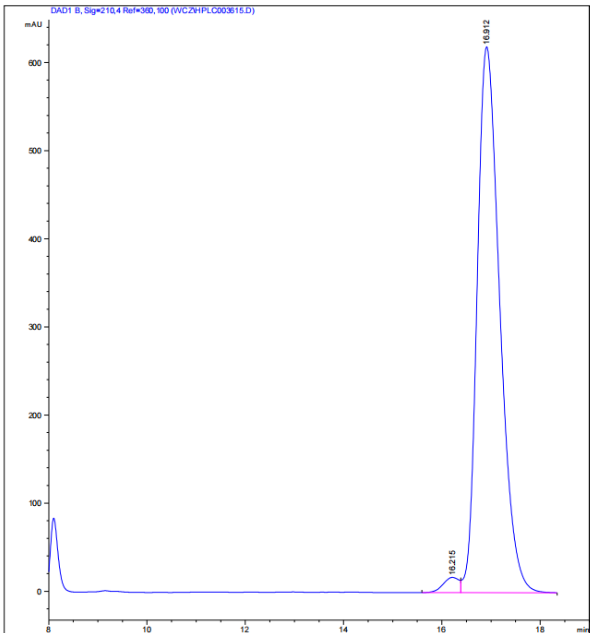

Peak	Ret.Time [min]	Type	Width [min]	Area[Mau*s]	Height [mAU]	Area%
1	16.215	BV	0.3413	383.98489	17.27590	1.9076
2	16.912	VBA	0.4940	1.97452e4	619.33215	98.0924

HPLC profiles for aldol adduct **3k**

AS-H, *i*-PrOH/Hex 15/85, flow rate = 1.0 mL/min

Racemic product

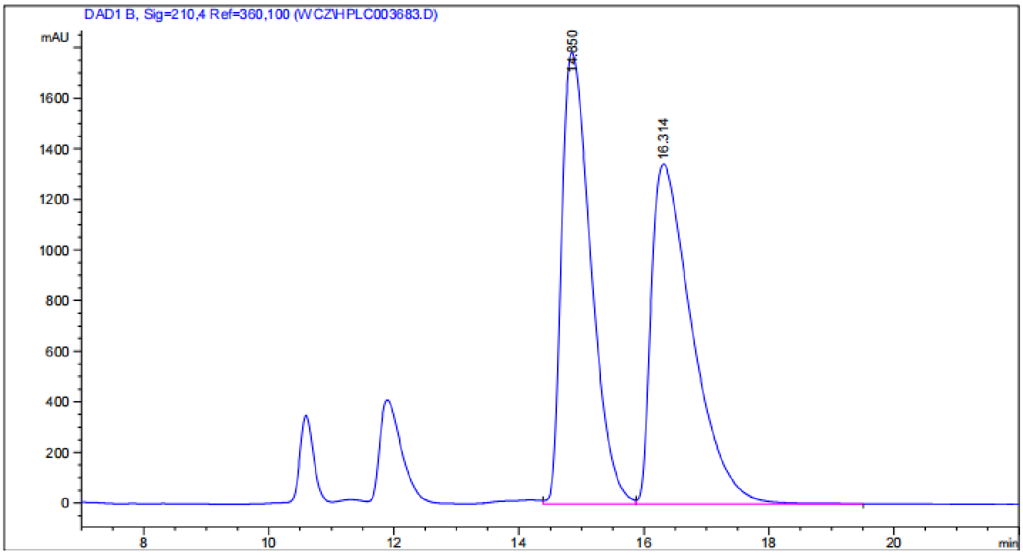

Peak	Ret.Time [min]	Type	Width [min]	Area[Mau*s]	Height [mAU]	Area%
#	[min]		[min]	[mAU*s]	[mAU]	%
1	14.850	VV	0.5091	5.82311e4	1782.80029	48.8932
2	16.314	VB	0.6909	6.08674e4	1342.58069	51.1068

Asymmetric catalytic product

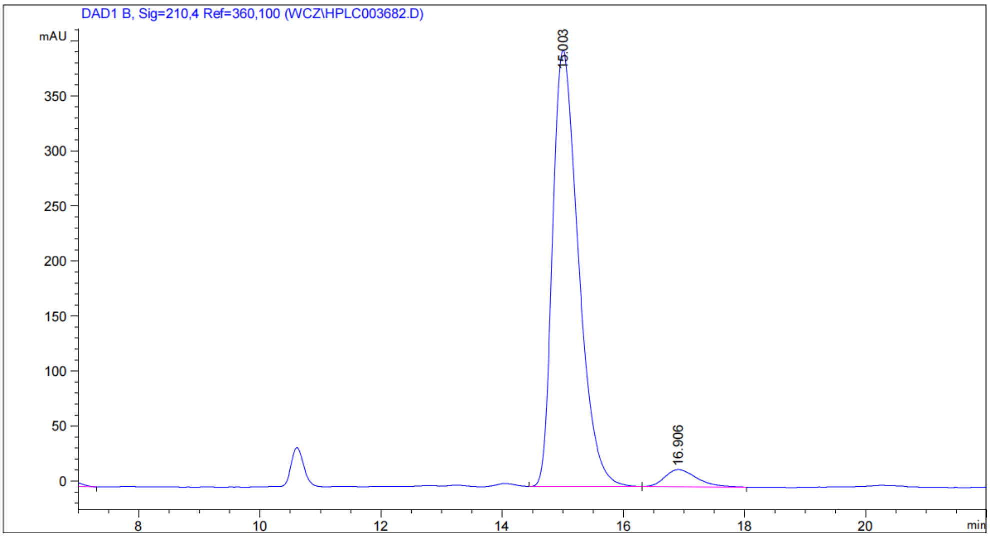

Peak	Ret.Time [min]	Type	Width [min]	Area[Mau*s]	Height [mAU]	Area%
1	15.003	BB	0.4548	1.17161e4	396.42722	95.3548
2	16.906	BB	0.5511	570.74664	15.59202	4.6452

HPLC profiles for aldol adduct **3l**

OD-H, *i*-PrOH/Hex 20/80, flow rate = 1.0 mL/min

Racemic product

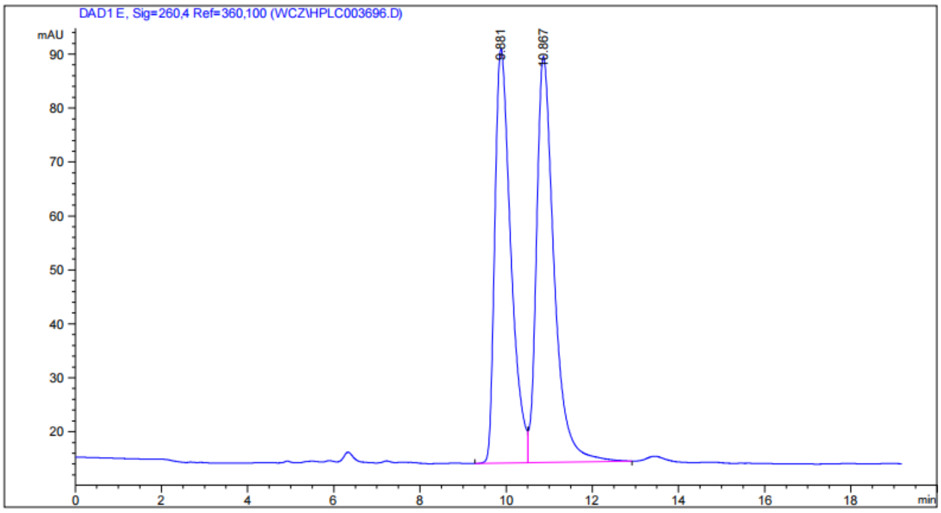

Peak	Ret.Time [min]	Type	Width [min]	Area[Mau*s]	Height [mAU]	Area%
1	9.881	BV	0.3946	1979.20239	76.82684	48.1040
2	10.867	VB	0.4289	2135.21973	75.29314	51.8960

Asymmetric catalytic product

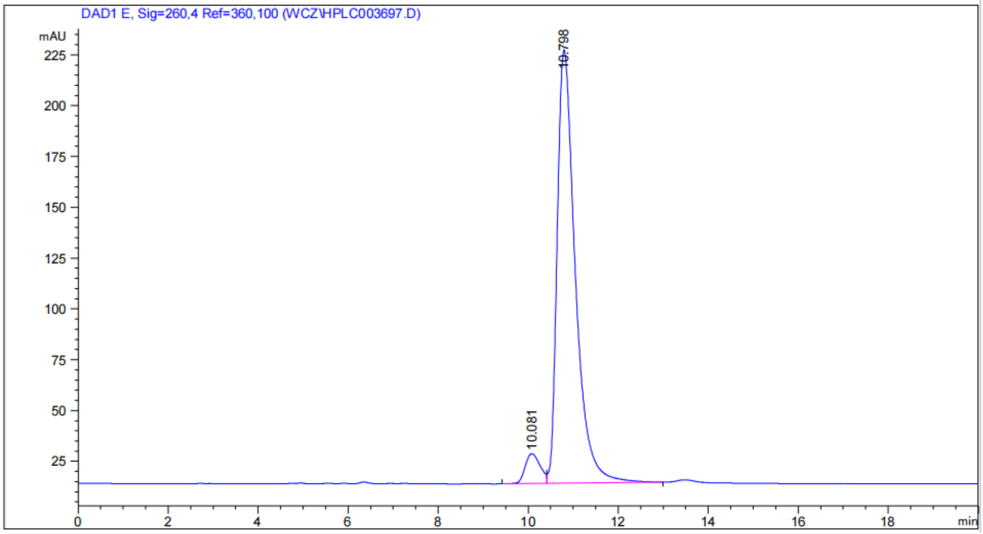

Peak	Ret.Time [min]	Type	Width [min]	Area[Mau*s]	Height [mAU]	Area%
1	10.081	BV	0.3603	340.70801	14.70577	5.4165
2	10.798	VB	0.4237	5949.44580	213.11494	94.5835

HPLC profiles for aldol adduct **3m**

AS-H, *i*-PrOH/Hex 40/60, flow rate = 1.0 mL/min

Racemic product

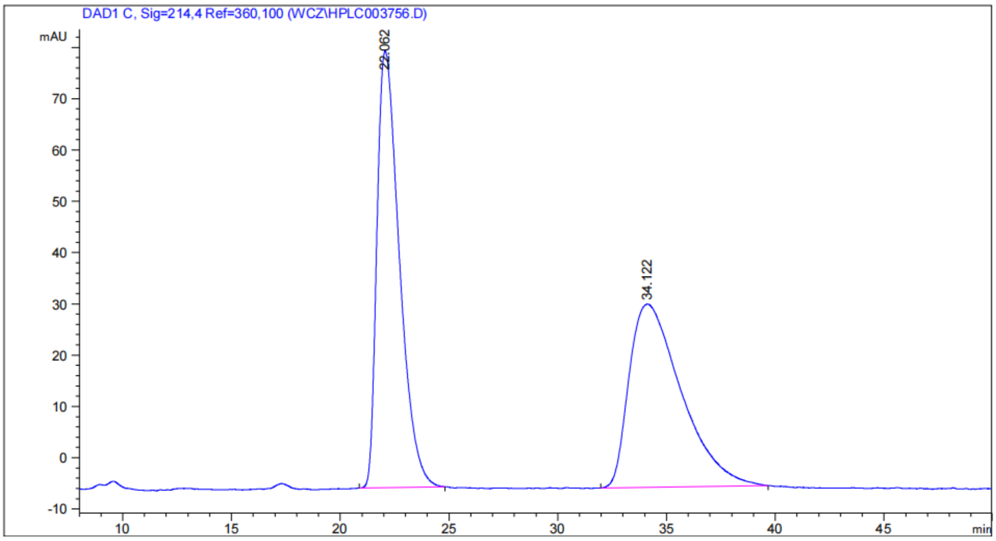

Peak	Ret.Time [min]	Type	Width [min]	Area[Mau*s]	Height [mAU]	Area%
1	22.062	BB	1.0875	6099.33936	85.16624	50.5693
2	34.122	BB	2.2783	5961.99902	35.73761	49.4307

Asymmetric catalytic product

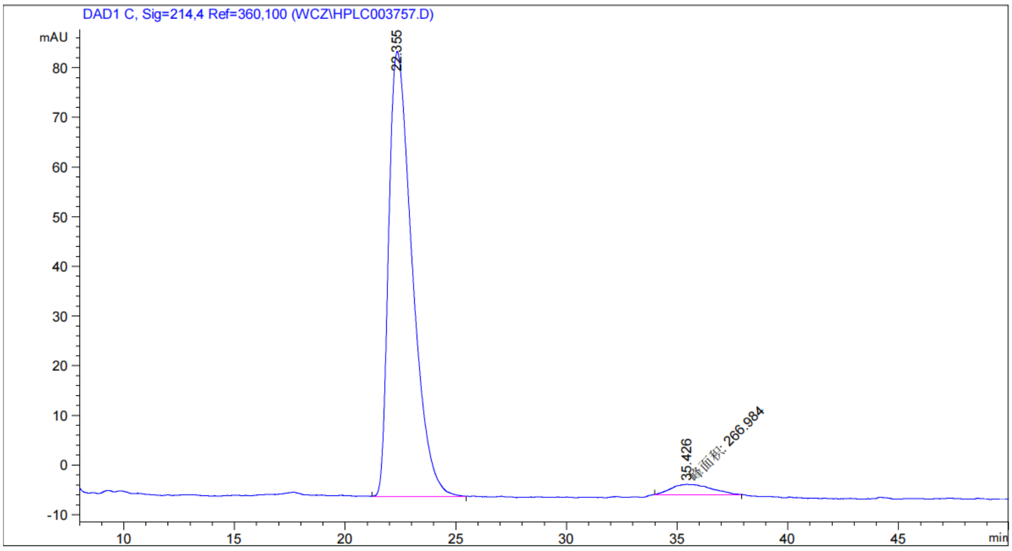

Peak	Ret.Time [min]	Type	Width [min]	Area[Mau*s]	Height [mAU]	Area%
1	22.355	BB	1.1083	6585.91553	89.52574	96.1041
2	35.426	MM	2.1153	266.98392	2.10360	3.8959

HPLC profiles for aldol adduct **3n**

AD-H, *i*-PrOH/Hex 10/90, flow rate = 1.0 mL/min

Racemic product

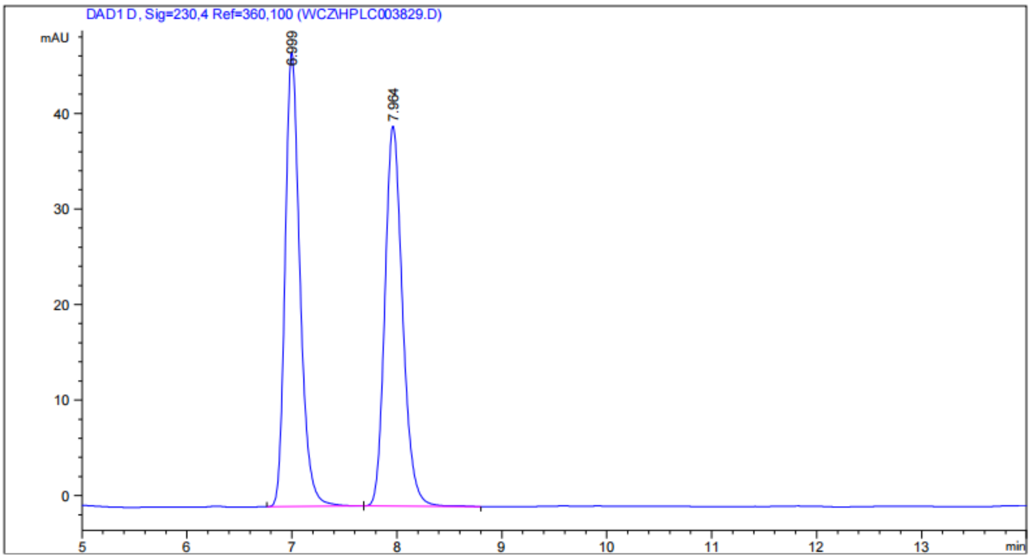

Peak	Ret.Time [min]	Type	Width [min]	Area[Mau*s]	Height [mAU]	Area%
1	6.999	BB	0.1505	466.01334	47.47792	50.0646
2	7.964	BB	0.1803	464.81012	39.77437	49.9354

Asymmetric catalytic product

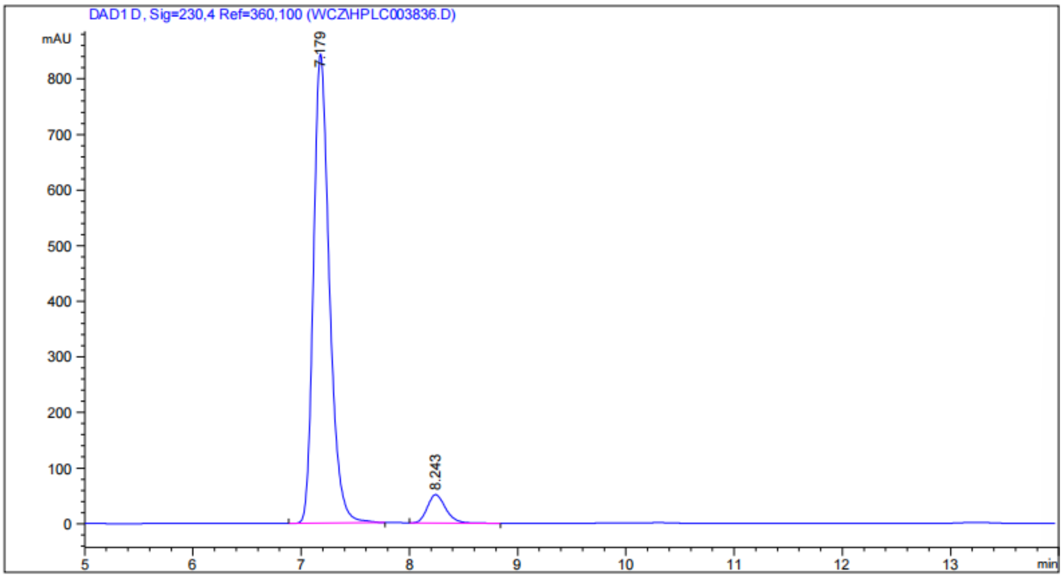

Peak	Ret.Time [min]	Type	Width [min]	Area[Mau*s]	Height [mAU]	Area%
1	7.179	BB	0.1571	8608.34180	843.13580	93.4442
2	8.243	BB	0.1819	603.93488	51.08908	6.5558

HPLC data for aldol adduct **5a**

AS-H, *i*-PrOH/Hex 10/90, flow rate = 1.0 mL/min

Racemic product

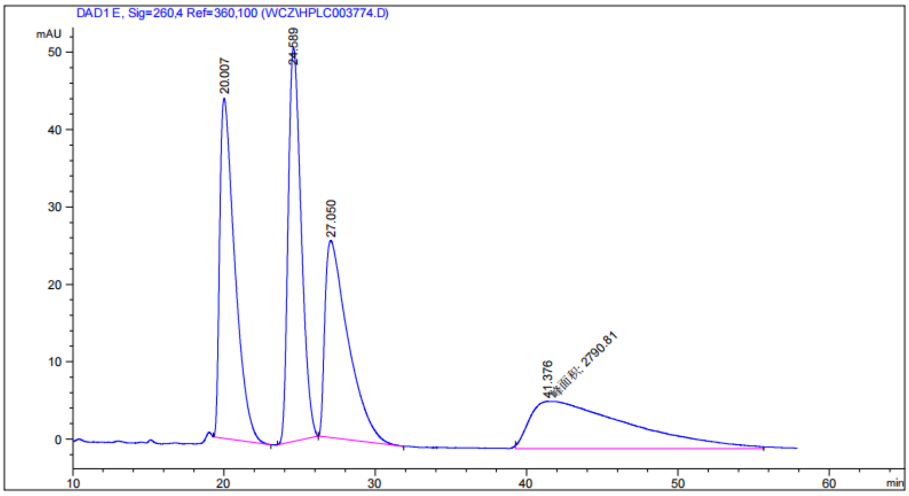

Peak	Ret.Time [min]	Type	Width [min]	Area[Mau*s]	Height [mAU]	Area%
1	20.007	BB	1.0250	3055.15454	43.95235	26.3365
2	24.589	BB	0.9270	3043.92041	50.95270	26.2396
3	27.050	BB	1.4941	2710.58374	25.46068	23.3661
4	41.376	MM	7.6034	2790.81372	6.11749	24.0578

Asymmetric catalytic product

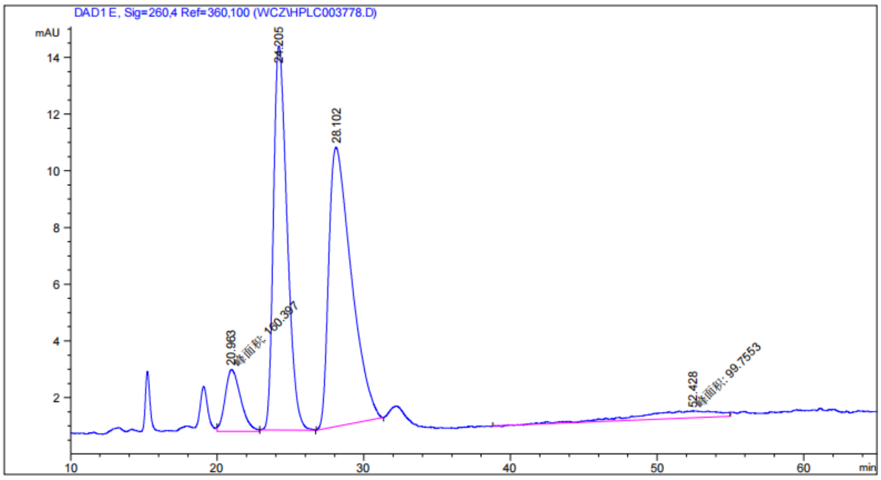

Peak	Ret.Time [min]	Type	Width [min]	Area[Mau*s]	Height [mAU]	Area%
1	20.963	MM	1.2242	160.39726	2.18363	7.3259
2	24.205	BB	0.9913	875.24530	13.55556	39.9757
3	28.102	BB	1.4597	1054.04614	9.85575	48.1422
4	52.428	MM	6.8675	99.75529	2.42095e-1	4.5562

HPLC data for aldol adduct **5b**

AS-H, *i*-PrOH/Hex 10/90, flow rate = 1.0 mL/min

Racemic product

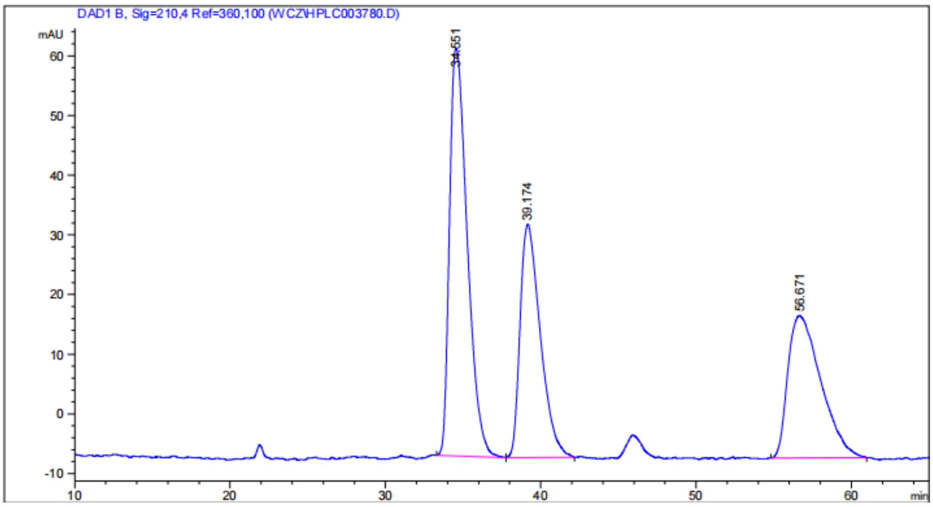

Peak	Ret.Time [min]	Type	Width [min]	Area[Mau*s]	Height [mAU]	Area%
1	34.551	BB	1.2314	5425.05859	68.24712	43.4870
2	39.174	BB	1.3514	3530.73657	39.10375	28.3022
3	56.671	BB	1.8111	3519.33032	23.85674	28.2108

Asymmetric catalytic product

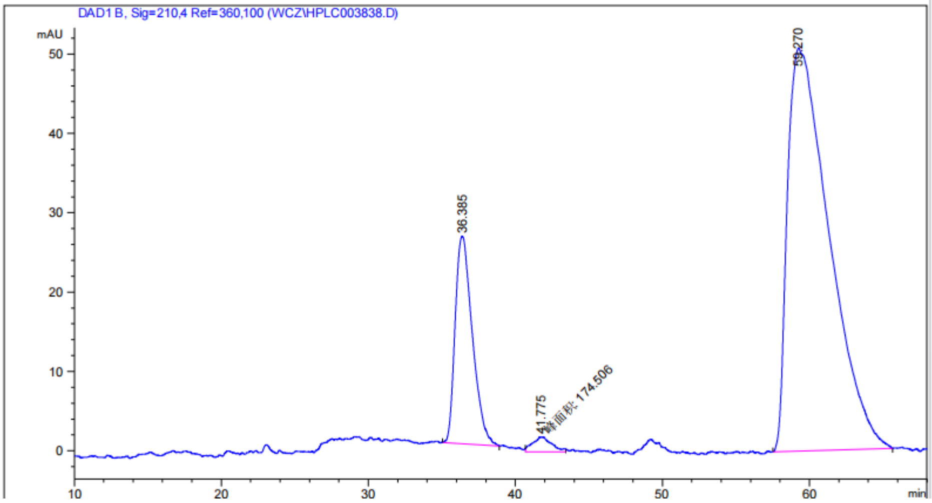

Peak	Ret.Time [min]	Type	Width [min]	Area[Mau*s]	Height [mAU]	Area%
1	36.385	BB	1.1769	2126.15381	26.14150	17.2603
2	41.775	MM	1.5256	174.50610	1.90643	1.4167
3	59.270	BB	2.4061	1.00175e4	50.81232	81.3230

HPLC profiles for aldol adduct **5c**

AS-H, *i*-PrOH/Hex 15/85, flow rate = 1.0 mL/min

Racemic product

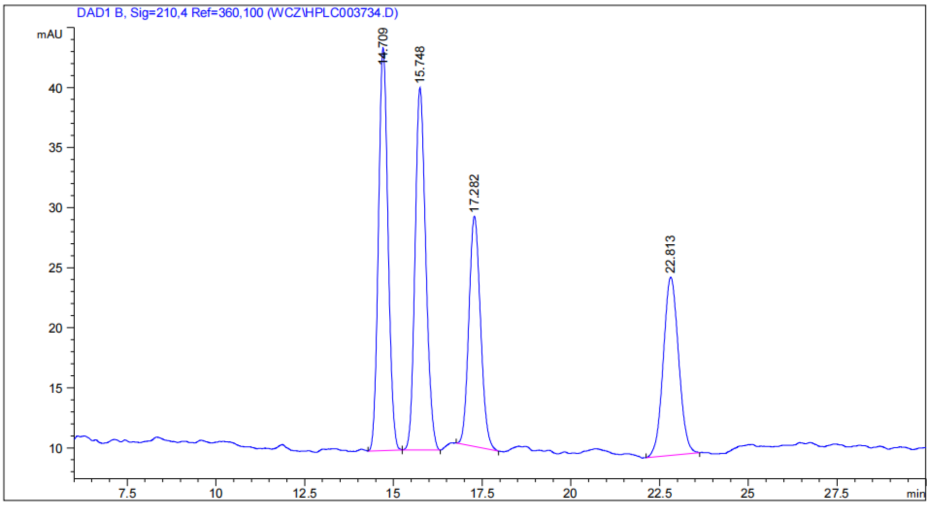

Peak	Ret.Time [min]	Type	Width [min]	Area[Mau*s]	Height [mAU]	Area%
1	14.709	BB	0.2928	628.89124	33.53823	29.3203
2	15.748	BB	0.3267	632.54614	30.15775	29.4907
3	17.282	BB	0.3569	438.76962	19.17785	20.4564
4	22.813	BB	0.4679	444.69247	14.82708	20.7326

Asymmetric catalytic product

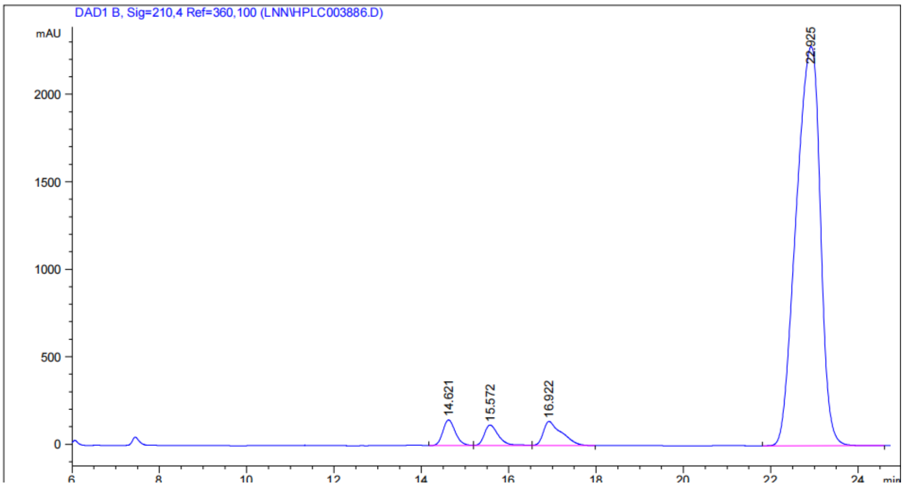

Peak	Ret.Time [min]	Type	Width [min]	Area[Mau*s]	Height [mAU]	Area%
1	14.621	VV	0.3077	2945.36646	147.05167	3.0252
2	15.572	VV	0.3461	2675.70361	117.29309	2.7483
3	16.922	VB	0.4015	3989.55664	137.91690	4.0977
4	22.925	BB	0.6328	8.77491e4	2281.23828	90.1287

HPLC profiles for aldol adduct **(5d)**

OJ-H, *i*-PrOH/Hex 10/90, flow rate = 1.0 mL/min

Racemic *anti*-**5d** and racemic *syn*-**5d**:

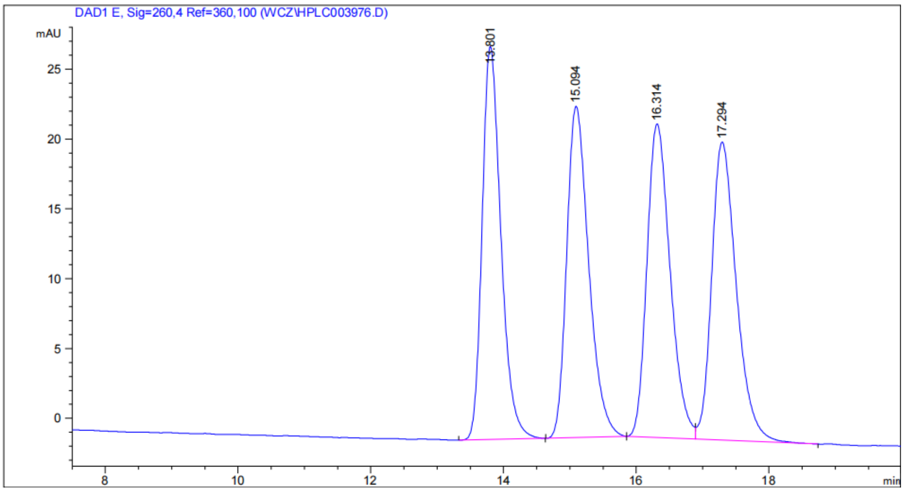

Peak	Ret.Time [min]	Type	Width [min]	Area[Mau*s]	Height [mAU]	Area%
1	13.801	BB	0.3014	553.18799	28.13572	25.1730
2	15.094	BB	0.3529	546.69916	23.71664	24.8778
3	16.314	BV	0.3736	545.82788	22.45365	24.8381
4	17.294	VB	0.3936	551.82733	21.34453	25.1111

Racemic *anti*-**5d** with a small amount of *syn*-**5d**:

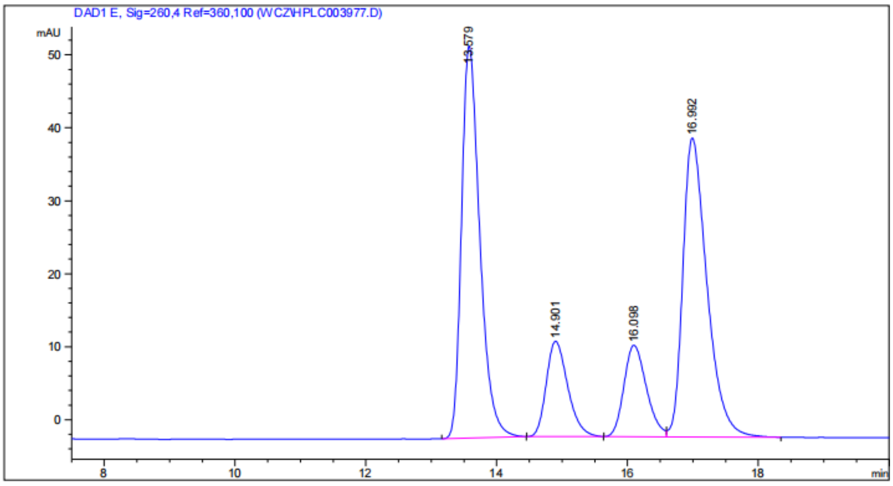

Peak	Ret.Time [min]	Type	Width [min]	Area[Mau*s]	Height [mAU]	Area%
1	13.579	BB	0.2965	1042.11133	53.68844	38.7866
2	14.901	BB	0.3527	298.44476	13.05601	11.1079
3	16.098	BV	0.3686	299.20520	12.53092	11.1362
4	16.992	VB	0.3902	1047.01843	40.96402	38.9693

Racemic *syn*-**5d**:

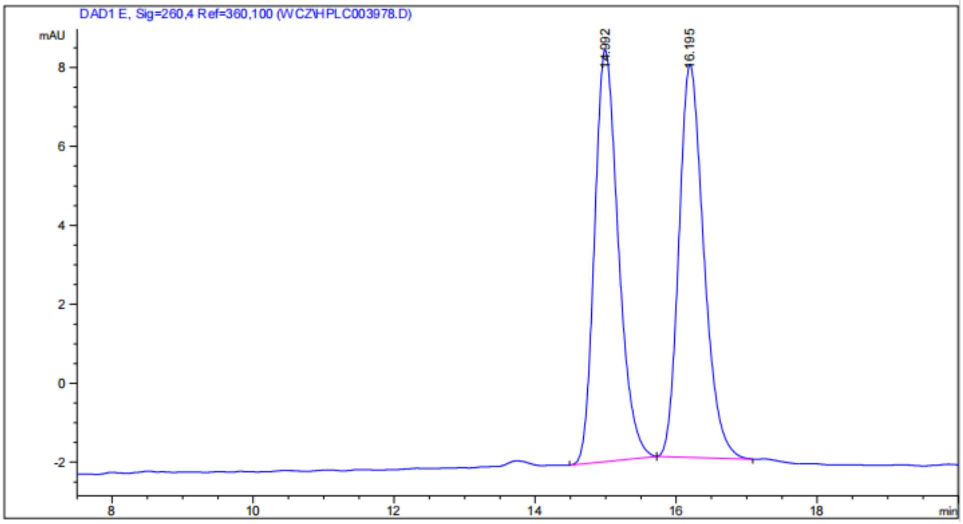

Peak	Ret.Time [min]	Type	Width [min]	Area[Mau*s]	Height [mAU]	Area%
1	14.992	BB	0.3594	244.35245	10.42561	50.0852
2	16.195	BB	0.3754	243.52147	9.95371	49.9148

Asymmetric catalytic product

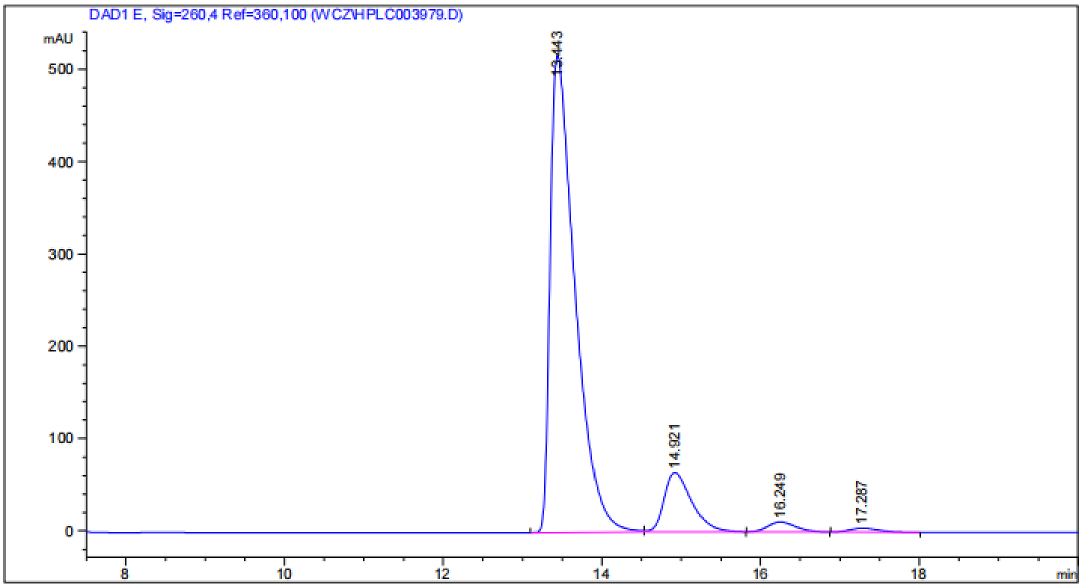

Peak	Ret.Time [min]	Type	Width [min]	Area[Mau*s]	Height [mAU]	Area%
1	13.443	BV	0.3249	1.12988e4	517.08978	85.3837
2	14.921	VB	0.3686	1565.74036	64.64348	11.8321
3	16.249	BB	0.3818	260.27567	10.70069	1.9669
4	17.287	BB	0.3872	108.15789	4.33398	0.8173

HPLC profiles for aldol adduct **(5e)**

OJ-H, *i*-PrOH/Hex 10/90, flow rate = 1.0 mL/min

Racemic product

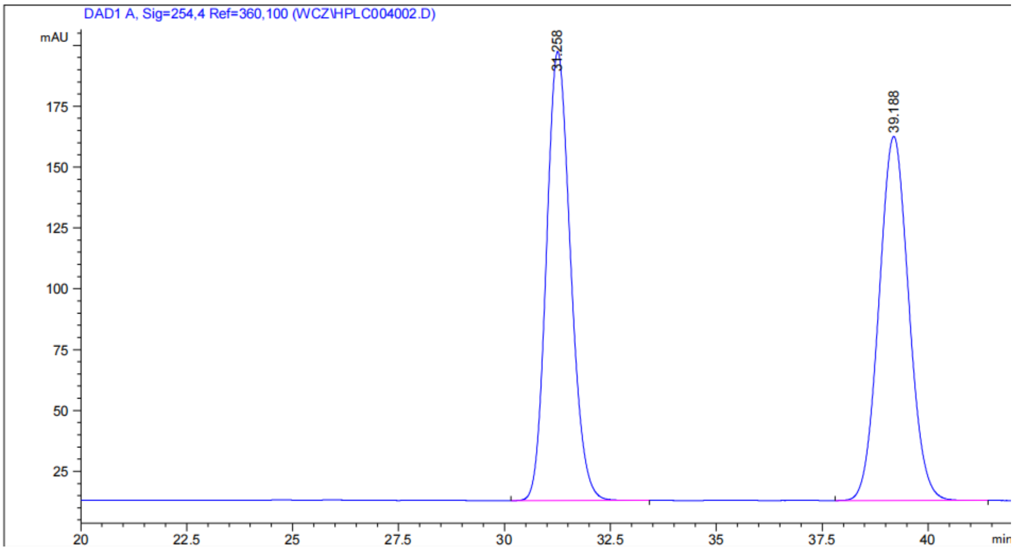

Peak	Ret.Time [min]	Type	Width [min]	Area[Mau*s]	Height [mAU]	Area%
1	31.258	BB	0.6162	7306.41553	184.43570	50.0056
2	39.188	BB	0.7657	7304.77148	149.60014	49.9944

Asymmetric catalytic product

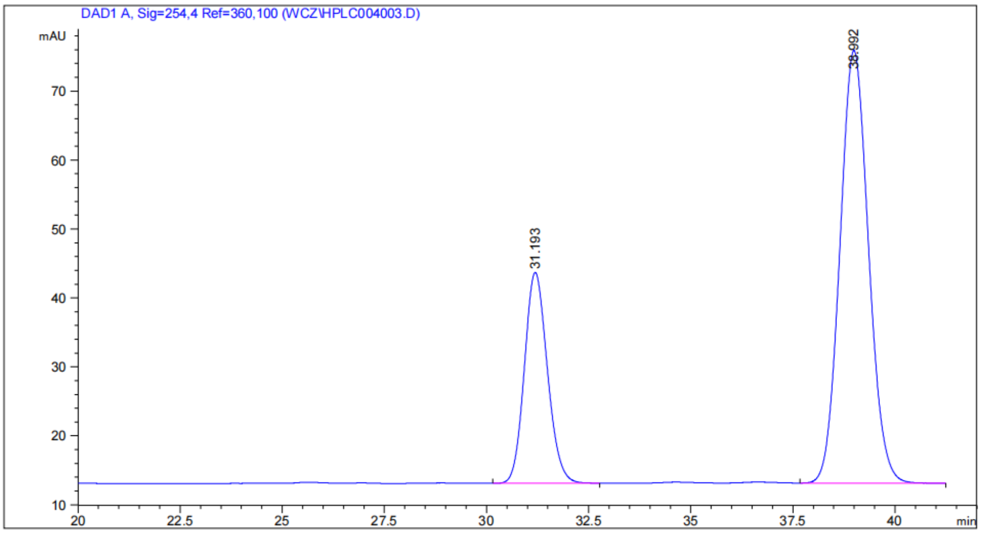

Peak	Ret.Time [min]	Type	Width [min]	Area[Mau*s]	Height [mAU]	Area%
1	31.193	BB	0.6071	1198.12854	30.57926	28.2643
2	38.992	BBA	0.7553	3040.88281	62.76026	71.7357
